# Huiyang Shengji decoction promotes wound healing in diabetic mice by activating the EGFR/PI3K/ATK pathway

**DOI:** 10.1186/s13020-021-00497-0

**Published:** 2021-11-02

**Authors:** Qingwu Liu, Jinchao Zhang, Xuyang Han, Jia Chen, Yating Zhai, Yan Lin, Huike Ma, Fang Feng, Xiujuan He, Ping Li

**Affiliations:** 1 Beijing Institute of Traditional Chinese Medicine, Beijing Hospital of Traditional Chinese Medicine, Capital Medical University, Beijing, China; 2 Department of Dermatology, China-Japan Friendship Hospital, Beijing, China; 3 Institute of Acupuncture and Moxibustion, China Academy of Chinese Medical Sciences, Beijing, China; 4 School of Clinical Medicine, Beijing University of Chinese Medicine, Beijing, China

**Keywords:** Wound healing, Traditional Chinese medicine, Huiyang Shengji decoction (HYSJD)

## Abstract

**Background:**

Common chronic wounds include diabetic ulcers, venous ulcers, and pressure ulcers. The traditional Chinese medicine Huiyang Shengji decoction (HYSJD) has been shown to promote the healing of diabetic chronic wounds, however, its pharmacological mechanism is still unclear.

**Purpose:**

This study aimed to determine the mechanism of HYSJD in promoting the healing of diabetic chronic skin ulcers.

**Methods:**

Ultra-performance liquid chromatography was combined with tandem mass spectrometry (UPLC-MS/MS) to analyze the main components of HYSJD and the absorbed components in mouse serum at 30 min after oral administration of HYSJD. db/db mouse models for chronic skin ulcers were constructed by full-thickness skin resection. Wound tissues at day 7 post wound formation were used to perform microarray analysis of growth factors and chemokine expression. GO and KEGG enrichment analysis was performed on differentially expressed proteins. ELISA assays were used to measure differential expressed cytokines in the serum and Western blot analysis was used to determine the expression levels of related pathway proteins in the skin wounds.

**Results:**

UPLC-MS/MS analysis showed that the main chemical components of HYSJD were flavonoids, terpenes, alkaloids, phenylpropanoids, and carbohydrates. At 30 min after oral administration of HYSJD, five absorbed components were detected in the serum, these included formononetin, calycosin, hypaconitine, calycosin-7-glucoside, and sinapic acid. HYSJD was found to increase the wound healing rate in chronic skin ulcers in db/db mice at days 3, 7, and 14 post wound formation, and promote the proliferation of epidermal cells. Two proteins that were differentially expressed between the different groups, i.e., IGF-1 and EGFR, were further validated. Serum ELISA assays showed that serum EGFR in the HYSJD treatment group was significantly increased. KEGG pathway analysis suggested that the PI3K/AKT pathway involved in HYSJD promoting the proliferation of epidermal cells in chronic wounds in db/db mice. Experimental verification showed that HYSJD activated the PI3K/AKT signaling pathway in mouse wound skin.

**Conclusion:**

HYSJD promotes the proliferation of epidermal cells in chronic diabetic wounds by increasing EGFR expression in the wounds and activating the PI3K/AKT signaling pathway. Our study provides an experimental basis for the pharmacological mechanism of HYSJD.

**Supplementary Information:**

The online version contains supplementary material available at 10.1186/s13020-021-00497-0.

## Introduction

Chronic wounds refer to wounds that cannot achieve anatomical and functional integrity through a normal, orderly, and timely repair process [1]. Common chronic wounds include diabetic ulcers, venous ulcers, and pressure sores. The prevalence of diabetes in China has soared from 0.67% in 1980 to 10.4% in 2013 [2], and diabetic ulcers have been identified as the most common complication in diabetic patients [3]. Repeated necrotic tissue debridement is a commonly used clinical treatment for diabetic ulcers, however, about 14–20% of diabetic ulcer patients still must undergo limb amputations [4], resulting in disability or even death [5].

Wound healing is a complex and dynamic biological process, involving the coordination of a variety of cells, and requires the participation and regulation of a variety of cytokines [6]. Cytokines are a class of small molecular proteins with biological activity produced by a variety of immune and non-immune cells. These include chemokines, interleukins, interferons, and growth factors. They form a complex interaction network with potential autocrine, paracrine, and endocrine functions. Through cell surface receptor binding, a series of complex signal transduction pathways are induced to regulate the growth, differentiation, and metabolism of specific cell populations [7, 8]. The lack of cytokine signals required for the normal wound repair process leads to reduced angiogenesis, granulation tissue formation, and epithelialization process, which are factors leading to poor healing of diabetic wounds [9]. Growth factors (GFs), including platelet-derived GF (PDGF), vascular endothelial GF (VEGF), Epidermal GF (EGF), fibroblast GF (FGF), transforming GF (TGF), keratinocyte GF (KGF), insulin-like GF (IGF), and other cytokines and their receptor expression contribute to the re-epithelialization of diabetic wounds. Angiogenesis and extracellular matrix synthesis are very important for the healing process of diabetic ulcer wounds [10–12]. Among them, the topical application of PDGF, VEGF, EGF, FGF, and TGF-β1 has been used in clinical trials for the treatment of diabetic ulcers [13].

Due to the high diffusion rate of cytokines and short half-life in the body [14], topical application of cytokines leads to the rapid spread and drying of the open wound [15]. Diabetic ulcers are often complicated with localized ulcers, microbial infections [16], elevated protease levels, reduced synthesis, and function of growth factors [17]. However, the cost of cytokine treatment is relatively expensive, which adds to the economic burden for patients with diabetic ulcers. Hence, regulating the expression of endogenous cytokines may be an alternative treatment strategy for treating diabetic ulcers [18].

HYSJD is a traditional Chinese medicine prescription used by Beijing Hospital of Traditional Chinese Medicine for the treatment of chronic skin ulcers. It is composed of *Astragli Radix*, *Atractylodis Macrocephalae Rhizoma*, *Atractylodis Rhizoma*, *Poria*, *Cinnamomi Cortex*, *Aconiti Lateralis Radix Praeparata, Cervi Cornu Degelatinatum, Chaenomelis Fructus, Sinapis Semen, Rehmanniae Radix Praeparata, Angelicae Dahuricae Radix,* and *Glehniae Radix* [19]. It can reduce wound pain, reduce wound secretions, and alleviate type 2 diabetes. HYSJD has been proven to promote wound healing for decades [20, 21]. However, the pharmacological mechanism of HYSJD for the treatment of diabetic chronic wounds is still unclear.

To understand the mechanism, we used db/db diabetic mice to establish a diabetic chronic wound model. By observing the effect of HYSJD on the expression of cytokines in the wounds of db/db diabetic mice, we investigated the mechanism by which HYSJD promotes the healing of diabetic ulcers.

## Materials and methods

### Preparation of HYSJD

HYSJD (*Astragli Radix* 30 g, *Atractylodis Macrocephalae Rhizoma* 10 g, *Atractylodis Rhizoma* 10 g, *Poria* 10 g, *Cinnamomi Cortex* 10 g, *Aconiti Lateralis Radix Praeparata* 10 g, *Cervi Cornu Degelatinatum* 10 g, *Chaenomelis Fructus* 10 g, *Sinapis Semen* 10 g, *Rehmanniae Radix Praeparata* 10 g, *Angelicae Dahuricae Radix* 10 g, and *Glehniae Radix* 15 g) was obtained from the Capital Medical University, Chinese Pharmacy of Beijing Hospital of Traditional Chinese Medicine (Beijing, China). 155 g of HYSJD was soaked for 1 h and then extracted twice with water to make the HYSJD concoction. The extract was filtered and concentrated to 78 mL, with a mass concentration of 1.993 g/mL. The HYSJD solution was then vacuum freeze-dried to obtain a freeze-dried powder preparation.

### Ultra performance liquid chromatography-tandem mass spectrometry (UPLC-MS/MS)

#### Analysis of HYSJD drug components

2 mL of HYSJD solution in a 15 mL centrifuge tube was diluted with five volumes of 70% methanol (V/V). The mixture was sonicated for 30 min, mixed well, and then 1 mL was transferred to a centrifuge tube. The sample was then centrifuged at 12,000 rpm for 10 min. The supernatant was placed in a liquid phase vial and then chromatographic separation was performed using a Waters' I class ultra-high performance liquid chromatography. Reversed-phase chromatography was used to analyze the decoction sample. The conditions for the gradient system was as follows: Chromatographic column: waters UPLC HSS T3 (1.8 μm 2.1 mm * 100 mm); mobile phase: A (acetonitrile) and B (water, 0.1% formic acid); elution program: see Table 1, flow rate: 0.4 mL/min; injection volume was 5.0 µL; column temperature: 45 °C.

**Table 1:** Gradient elution protocol

Time (min)	A (v%)	B (v%)
0	1	99
8	80	20
8.5	100	0
9.5	100	0
10	1	99
13	1	99

Acetonitrile (A); 0.1% formic acid (B)

**Fig. 1:**
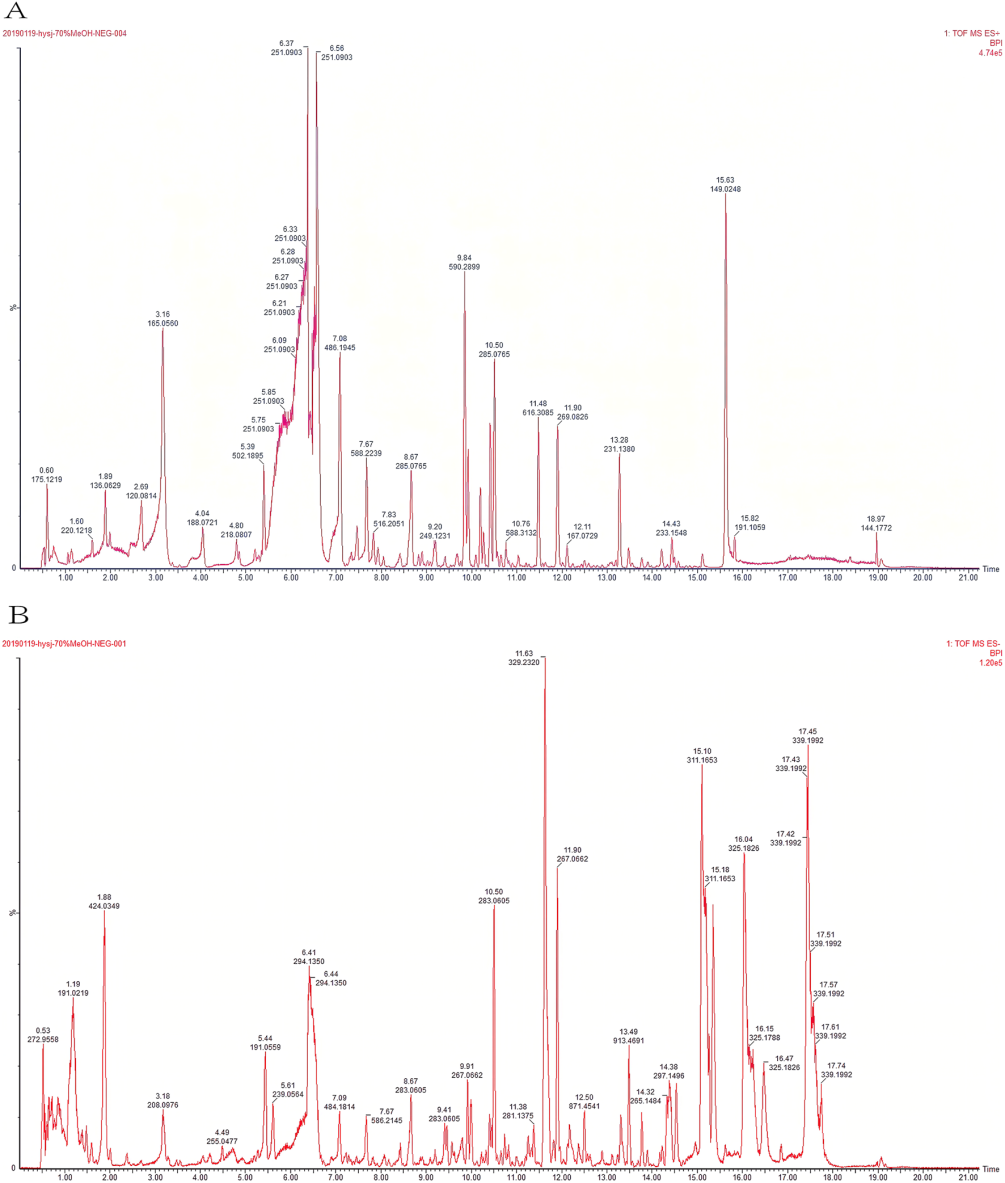

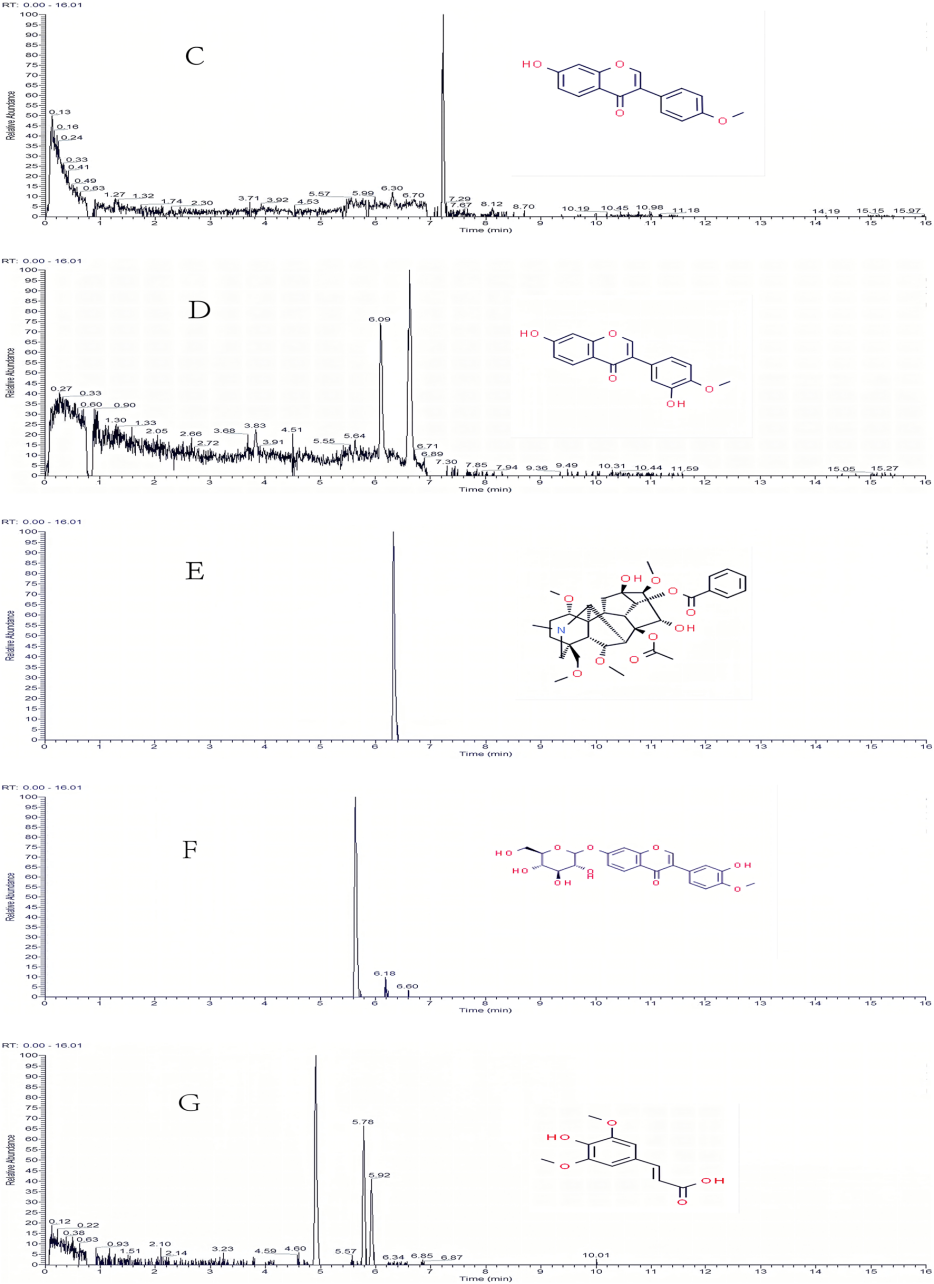
Chromatograms of HYSJD and the five main bioactive compounds in serum at 30 min after oral administration of HYSJD. **A** Total ion current of HYSJD (positive ions). **B** Total ion current of HYSJD (negative ions). **C** formononetin, **D** calycosin, **E** hypaconitine, **F** calycosin-7-glucoside, **G** sinapic acid

Mass spectrometry was performed using a quadrupole time-of-flight tandem mass spectrometer equipped with an electrospray ion source. Scanning mode: ESI+ESI−mode, capillary voltage: 3.0 kV and 2.5 kV, cone voltage 40 V, ion source temperature: 100 °C, desolvator temperature: 350 °C, cone gas flow rate: 50 L/h. The flow rate of the desolventizer was 800 L/h, the data collection rate: 0.2 s/scan, and the mass scan range: m/z 50–1500. Compound identification adopted the MSE scanning mode (data-dependent scanning mode), and the collision energy of the secondary mass spectrum was 10–60 v. The lock-mass technology was used to determine the accurate mass, 2 mg/L leucine-enkephalin (LE, ESI^+^: m/z 556.2771, ESI^−^: m/z 554.2615) solution was the lock-mass solution. The flow rate was 10 μL/min, and the switching frequency was 20 s/time. Masslynx 4.1 software was used for data collection and the UNIFI software for compound identification. The total ion current diagram for the HYSJD is shown in Fig. 1.

#### Analysis of the absorbed components in mouse serum after oral administration of HYSJD

Serum samples were collected at 30 min after oral administration of HYSJD solution (19.93 g/kg). 100 μL of serum was placed in a 1.5 mL centrifuge tube and sonicated for 1 h with 50 μL of methanol. The sample was then centrifuged at 12,000 rpm, 4 °C for 10 min. Afterward, 100 μL of the supernatant was transferred to a new vial. Chromatographic separation was performed using the Thermo Scientific's U3000 fast liquid chromatograph. Serum samples were analyzed using a reversed-phaseand hydrophilic chromatograph. Details are as follows; Column: waters UPLC HSS T3 (1.8 μm 2.1 mm * 100 mm); mobile phase: A (water, 0.1% formic acid) and B (acetonitrile); elution program: see Table 2, flow rate: 0.3 mL/min; injection amount = 1.0 µL; column temperature: 50 °C.

**Table 2:** C18 reversed phase chromatography elution sequence

Time (min)	A (v%)	B (v%)
0	95	5
2	95	5
5	50	50
10	50	50
12	0	100
14	0	100
14.1	95	5
16	95	5

H_2_O, 0.1% formic acid (A); Acetonitrile (B)

Mass spectrometry analysis was performed using a quadrupole orbital ion trap mass spectrometer equipped with a thermo electrospray ion source. The voltages of the positive and negative ion sources were 3.7 kV and 3.5 kV, respectively. The capillary heating temperature was 320 °C. Lift air pressure was 30 psi, and auxiliary air pressure was 10 psi. The volumetric heating evaporation temperature was 300 °C. Both lift and auxiliary gases were nitrogen. The collision gas was nitrogen, and the pressure was 1.5 mTorr. The first-level full scan parameters were as follows: resolution = 70,000, automatic gain control target = 1 × 10^6^, maximum isolation time = 50 ms, and the mass-to-charge ratio scan range was 50–1500. The liquid mass system was controlled using the Xcalibur 2.2 SP 1.48 software. Data acquisition and targeted metabolite quantitative processing was performed by the software.

### Animals and grouping

Male C57 BLKS/J db/db diabetic mice (9–10 weeks; body weight 35–49 g) and male C57BL/6J db/m mice (9–10 weeks; body weight 18–27 g) were purchased from the Laboratory Animal Center of Peking University Health Science Center (laboratory animal certificate number: SCXK (Beijing) 2016-0010). Mice were housed in single cages and had free access to food and water. All surgical procedures were approved by the Animal Ethics Committee of Beijing Institute of Traditional Chinese Medicine (No. 2018100203). Mice were housed for 1 week, weighed, and blood glucose levels (Sannuo Company) were measured using tail vein whole blood.

Mice were anesthetized with 1% intraperitoneal injection of sodium pentobarbital (60 mg/kg). A full-thickness skin excision wound with a diameter of 7 mm was made in the middle of the back using a skin punch. A digital camera (Canon, Japan) was used to take images of the wounds. db/m mice that did not undergo the skin punch procedure were considered the control group (Ctrl group), *n* = 16; 48 db/db mice that underwent the skin punch procedure were randomly divided into 3 groups: (1) Model group (Model group), *n* = 16; (3) Positive control group, *n* = 16, topical treatment with basic fibroblast growth factor (bFGF) (3.6 μg/mL, PT 6358, PeproTech, USA), dissolved in normal saline (50 μL) to prepare a drug-containing gelatin sponge dressing (Nanjing Jinling Pharmaceutical Factory), and was applied to the wound externally; (4) HYSJD group, *n* = 16, HYSJD (19.93 g/kg) treatment. The mice in the HYSJD group were administered HYSJD (0.4 mL) 6 h after surgery. The blank group, model group, and bFGF group were administered the same volume of sterile water (0.4 mL). Except for mice in the positive control group that were administered bFGF externally, all other groups were administered the same volume of normal saline (50 μL) gelatin sponge for external wound application. The gelatin sponge was wrapped in a disposable sterile dressing (Shandong Donghua Medical Technology Co., Ltd.). Gavage was administered once a day, the wound dressing was changed every other day, during which images of the wound were taken.

On the 7th day post wound formation, blood and skin samples were collected. ELISA quantitative analysis of serum samples was performed. A section of the wound surface was used for the measurement of tissue growth factors and chemokine levels, while the remaining wound skin was fixed in 10% formalin for morphology analysis. On the 14th day of wound formation, wound tissues from the remaining mice were fixed with 10% formaldehyde solution for histo-morphological examination.

### Wound healing rate

The wounds were imaged and then Image-Pro Plus 6.0 software was used to calculate the wound area. Wound healing rate was calculated using the following formula: Wound healing rate (%) = (initial wound area − wound area after administration)/initial wound area × 100%.

### HE staining

Excised skin was fixed with 4% paraformaldehyde, dehydrated, embedded in paraffin, and then sliced to 5 μm thickness. The slices were stained with Hematoxylin and Eosin (Beijing Zhongshan Jinqiao Company) and skin morphology changes were observed under a light microscope. Images were taken using an Axio Imager A2 Zeiss optical microscope (Carl Zeiss, Germany).

### Measurement of mouse wound tissue growth factor and chemokine levels using chip antibody arrays

#### Growth factor and chemokine antibody array

The AAM-GF-3-8, QAM-CHE-1-2 kit (Guangzhou Ruiboao Biotechnology Co., Ltd., China) was used to measure levels of growth factors and chemokines. The growth factor chip consisted of 30 proteins and the chemokine chip consisted of 25 proteins (see Additional file 1: Table S1). Each capture antibody was printed in four repeated dots in a sub-array. Each slide contained 16 identical sub-arrays. The sub-arrays were separated by a 16-hole sealed hybridization chamber to prevent cross-contamination of the samples. The antibody arrays were stored at − 20 °C until used.

#### Growth factor and cytokine expression levels using antibody arrays

Wound skin with a diameter of 1 cm from the center of the wound was excised seven days after wound formation. The skin was cut using sterile ophthalmic scissors. Proteins were extracted using tissue lysis buffer containing protease inhibitors. Tissues were ground using a low-speed electric grinder. The lysates were centrifuged at 4 °C (13,000 rpm, 20 min) and proteins in the supernatant were quantified using the BCA protein quantification kit (Thermo, USA). Lysates were diluted to the same concentration of 500 μg/mL. Based on the manufacturer's instructions, the Quantibody mouse cell growth factor and chemokine array 1 (RayBiotech, Inc, Norcross, GA, USA) were used to quantify the levels of growth factors and chemokines in the wounds. A chemiluminescence scanner was used to measure signal intensity. The scanning parameters were as follows: high-resolution laser scanner for Cy3 or the green channel (excitation frequency = 532 nm): Wavelength: 532 nm; Resolution: 10 μm: The original signal data was extracted using the MapPix 6.0 software. Data were analyzed using the Ray Biotech growth factor and chemokine antibody array software.

#### Bioinformatics analysis

Principal component analysis (PCA) and gene ontology analysis (GO) was used to identify differentially expressed proteins and their functions. (KEGG) enrichment analysis found significant enrichment for functions and pathways. Hierarchical cluster analysis was performed to identify different protein expression patterns. Analysis was performed using the following: PCA (R package “ggbiplot”), Hierarchical cluster analysis (R package “gplot”), GO analysis (R package “org.Hs.eg.” and “clusterProfiler”), and KEGG enrichment analysis (R package “clusterProfiler”) were performed using the open source program R (version 3.5.1).

### Enzyme-linked immunosorbent assay (ELISA)

Seven days after skin puncture, blood samples were collected, incubated for 2 h, and then centrifuged at 3000 rpm for 15 min. The serum was then harvested to measure EGFR and IGF-1 levels using the mouse EGFR and IGF-1 ELISA kit (Guangzhou Ruiboao Biotechnology Co., Ltd., China). A microplate reader was used to measure the absorbance (A) at 450 nm wavelength. EGFR and IGF-1 levels in the serum were calculated using the standard curve.

### Ki67 immunohistochemical staining

Ki67 staining was used to measure proliferating cells in the epidermis of the wound from each group. Fixed tissue sections underwent heat-mediated antigen recovery in citrate buffer (pH 6.0) and then blocked with 4% goat serum at room temperature for 30 min. Afterward, tissues were incubated with primary antibody (1:100, rabbit anti-mouse, Abcam) overnight at 4 °C and then incubated with the secondary antibody Alexa Fluor^®^ 488 antibody (1:600, goat anti-rabbit, 1910, GXY bioSci & Tech Co., Ltd.) at room temperature for 1 h. Nuclei were stained with 2-(4-amidino)-6-indolecarbamidine hydrochloride (DAPI; Beyotime). Ki67 staining of epidermal cells was observed under a 400× microscope. Image-Pro Plus 6.0 software was used to calculate the number of Ki67 positive cells in three random fields (400×).

### Western blot analysis

Wound tissues were lysed with RIPA buffer and total protein was extracted and quantified using the BCA kit (Thermo, USA). After protein denaturation at 100 °C for 10 min, an equal volume of loading buffer was added to the sample before SDS-PAGE electrophoresis. Proteins were then transferred to a PVDF membrane and blocked with 5% skim milk at room temperature for 1 h with gently shaking. Membranes were incubated with the following primary antibodies; AKT (1:1000, 4691S, Cell Signaling Technology), p-AKT (1:300, bs-0876R, Bioss), PI3K (1:500, 4292S, Cell Signaling Technology), P-PI3K (1:300,17366S, Cell Signaling Technology), or β-actin (1:5000, G1001, GXY bioSci & Tech Co., Ltd.) overnight at 4 °C. Afterward, Anti-Rabbit IgG (H + L) Antibody, DyLight™ 680-Labeled (1:5000, 5230-0402, SeraCare) was incubated at room temperature for 1 h. Protein bands were visualized using the far-infrared laser imaging system (LI-COR, USA). Gray value intensities of the bands were quantified using the Image J software.

### The effect of HYSJD on the Hacat cell proliferation

10,000 Hacat cells/well were seeded on to a 96-well plate. After 24 h, cell culture media was removed, and serum-free media was added and incubated for an additional 24 h. Cells were then treated with different concentrations of HYSJD for 24 h. After incubation, cells were washed with PBS and 10% CCK-8 detection solution was added and incubated for 2 absorbance was measured using a microplate reader at 450 nm wavelength.

### Statistical analysis

Experimental data were presented as “${\overline{\text{x}}} + {\text{s}}$”. SPSS 20.0 was used to analyze the data. One-way analysis of variance (ANOVA) was used to compare the statistical differences of three or more groups. GraphPad Prism software (GraphPad, La Jolla, USA) was used for graphical analysis (version 7.03). *P* value < 0.05 was considered statistically significant. In vitro experiments were performed in triplicate.

## Results

### HYSJD drug components and main absorbed components in serum

#### HYSJD drug components

We qualitatively analyzed the components of HYSJD using UPLC–MS/MS. The main components were flavonoids, terpenes, alkaloids, phenylpropanoids, and carbohydrates. Total ion current diagrams are shown in Fig. 1A, B. UPLC-MS/MS chromatogram for chromatographic peak measurement data and inference are shown in Table 3. The higher abundance peaks were for hypaconitine, neoline, calycosin, calycosin-7-glucoside, verbascose, formononetin, sinapine, etc., which were derived from *Aconiti Lateralis Radix Praeparata*, *Astragli Radix*, *Rehmanniae Radix Praeparata*, and *Sinapis Semen*.

**Table 3:** Chemical components of HYSJD

Number	Observed RT (min)	Ion mode	Neutral mass (Da)	Observed m/z	Mass error (ppm)	Molecular formula	Compound	Source
1	10.27	ESI+	300.09977	301.1087	5.4	C_23_H_28_O_10_	Astraisoflavan-7-O-β-d-glucoside	Astragalus
2	11.93	ESI+	629.32	630.327	− 0.4	C_33_H_45_NO_10_	3-Deoxyaconitine	Aconite
3	5.6	ESI−	194.05791	239.0583	9.1	C_10_H_10_O_4_	Isoferulic acid	Astragalus
4	7.11	ESI+	437.27774	438.2856	1.2	C_24_H_39_NO_6_	Neoline	Aconite
5	13.78	ESI−	868.48204	913.478	− 2.4	C_45_H_72_O_16_	Isoastragaloside I	Astragalus
6	10.5	ESI+	284.06847	285.078	7.9	C_16_H_12_O_5_	Calycosin	Astragalus
7	8.66	ESI+	446.1213	447.1294	1.9	C_22_H_22_O_10_	Calycosin-7-glucoside	Astragalus
8	1.43	ESI−	828.27468	827.2683	1	C_30_H_52_O_26_	Verbascose	Radix rehmanniae praeparata
9	11.9	ESI+	268.07356	269.083	7.9	C_16_H_12_O_4_	Formononetin	Astragalus
10	6.42	ESI+	309.15762	310.1662	4.2	C_16_H_24_NO_5_	Sinapine	Sinapis semen
11	13.09	ESI+	232.14633	233.1554	7.6	C_15_H_20_O_2_	Atractylenolide II	Atractylodes, atractylodes lancea
12	0.74	ESI+	504.16903	543.1321	− 0.1	C_18_H_32_O_16_	Raffinose	Radix rehmanniae praeparata
13	16.86	ESI−	456.36035	455.3527	− 0.9	C_30_H_46_O_3_	Dehydrotrametenolic acid	Poria
14	11.48	ESI+	615.30435	616.3118	0.4	C_33_H_45_NO_10_	Hypaconitine	Aconite
15	3.13	ESI−	524.17412	569.1714	− 1.6	C_21_H_32_O_15_	Rehmannioside A	Radix rehmanniae praeparata

#### Identification of the main absorbed components in serum

The five main absorbed components, including formononetin, calycosin, hypaconitine, calycosin-7-glucoside, and sinapic acid, were identified in serum after oral administration of HYSJD by UPLC-MS/MS (see Table 4). Figure 1C–G showed the chromatograms of the five main absorbed components at 30 min after oral administration of HYSJD. The retention time of formononetin was approximately 7.22 min, calycosin was 6.64 min, hypoaconitine was 6.33 min, calycosin-7-glucoside was 5.63 min, and sinapic acid was 5.78 min.

**Table 4:** Five components measured in the serum at 30 min after oral administration of HYSJD

Number	Compound	Molecular formula	Observed RT (min)	Neutral mass (Da)	Observed m/z	Ion type	Mass error (ppm)	MS/MS
1	Formononetin	C_16_H_12_O_4_	7.22	269.0814	269.0802	M+H	4.45	269.0802/149.0117/116.9858
2	Calycosin	C_16_H_12_O_5_	6.64	285.0763	285.0751	M+H	4.2	285.0715/217.0489/149.0118/116.9858
3	Hypaconitine	C_33_H_45_NO_10_	6.33	616.3122	616.3109	M+H	2.1	616.3109/431.1321/247.0931/207.1009
4	Calycosin-7-glucoside	C_22_H_22_O_10_	5.63	447.1291	447.1279	M+H	2.63	447.1279/403.25465/372.2164/149.0120
5	Sinapic acid	C_11_H_12_O_5_	5.78	225.0763	225.0751	M+H	5.33	225.0751/207.0650

### Body weight and blood glucose levels of diabetic mice

**Fig. 2:**
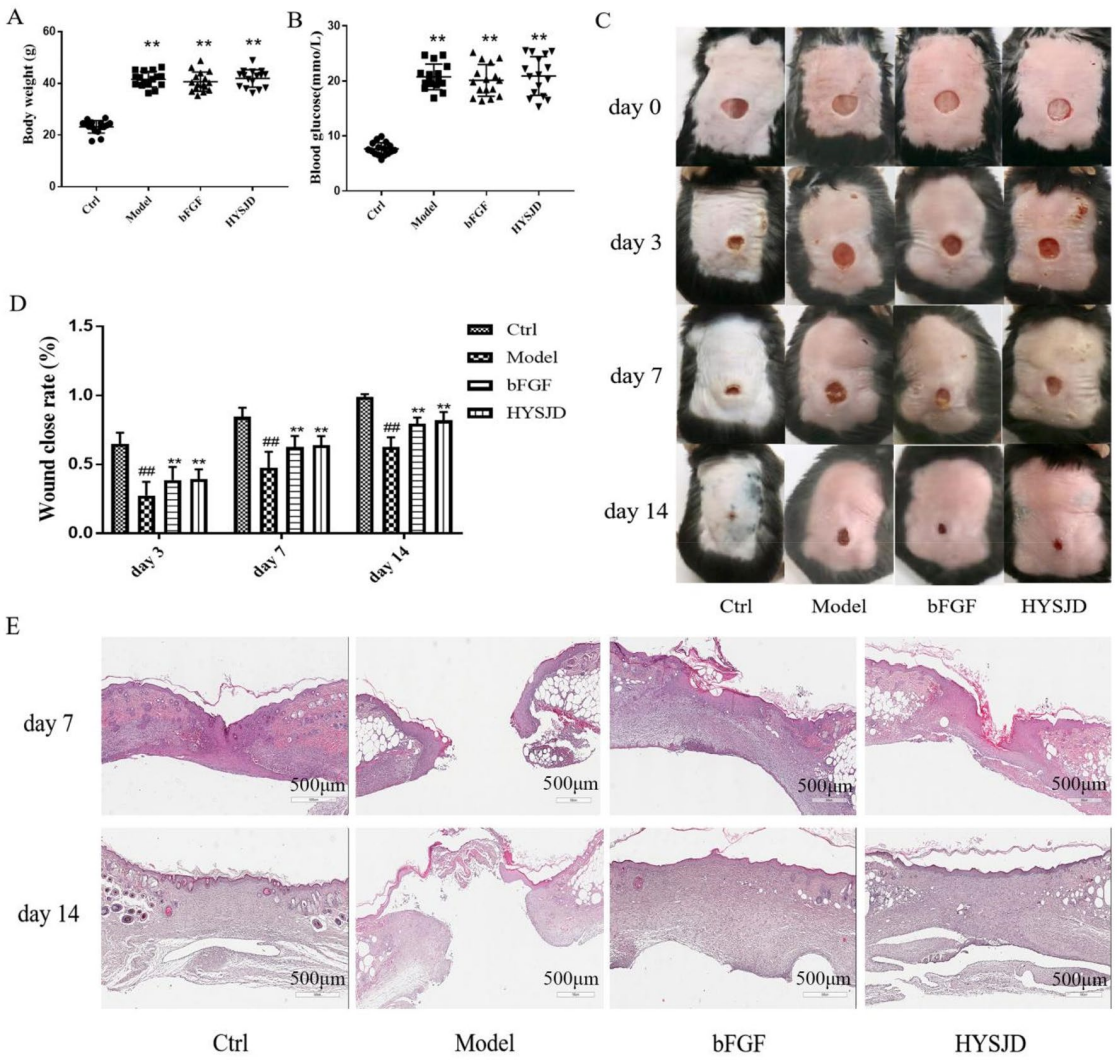
HYSJD treatment accelerates wound closure in db/db diabetic mice. **A** Body weight of db/m and db/db mice. **B** Blood glucose levels (*n* = 16 in each group). **C** Representative images of wound healing at days 3, 7, and 14 after wound surgery. **D** Wound closure analyzed using Image Pro (*n* = 16, at day 7; *n* = 8, at day 14). **E** Hematoxylin and eosin staining of the wound healing tissues ondays 7 and 14 after wound surgery. Scale bar: 500 μm. Data were expressed as mean ± SD, and significance was expressed as ^##^*P* < 0.01 vs Ctrl and **P < 0.01 vs Model. Ctrl, control; bFGF, basic fibroblast growth factor; HYSJD, Huiyang Shengji decoction

Compared to db/m mice, db/db mice were bulimia and obese, drank more water, had polyuria, and movement was slower. Compared to db/m mice, the body weight and blood glucose levels of db/db diabetic mice were higher (*P* < 0.01). There were no significant differences in body weight and blood glucose levels between the groups of db/db diabetic mice (*P* > 0.05) (Fig. 2A, B).

### Effect of HYSJD on the wound healing rate in db/db mice

At 3, 7, and 14 days post wound formation, the wound healing rate in the model group was lower compared to the control group (*P* < 0.01). The wound healing rate in the HYSJD and bFGF group was higher compared to the model group (*P* < 0.01) (Fig. 2C, D). This demonstrated that HYSJD could promote chronic wound healing in db/db diabetic mice.

### Effect of HYSJD on wound histopathology in diabetic mice

HE staining showed that 7 days post wound formation, new epidermal cells were observed in each group. A large number of fibroblasts were observed in the wounds of the control group and were arranged evenly. The epidermal structure in the model group was incomplete, dermal fibroblasts were arranged disorderly, and increased inflammatory cell infiltration were observed. The epidermal structure in the bFGF and HYSJD groups were complete, with dense and regular fibroblasts and abundant capillaries. 14 days post wound formation, a small number of newly produced epidermal cells were observed in the model group, however, complete healing was not observed. The majority of the epidermis in the bFGF and HYSJD group had healed after 14 days (Fig. 2E).

### Identification of differential proteins

**Fig. 3:**
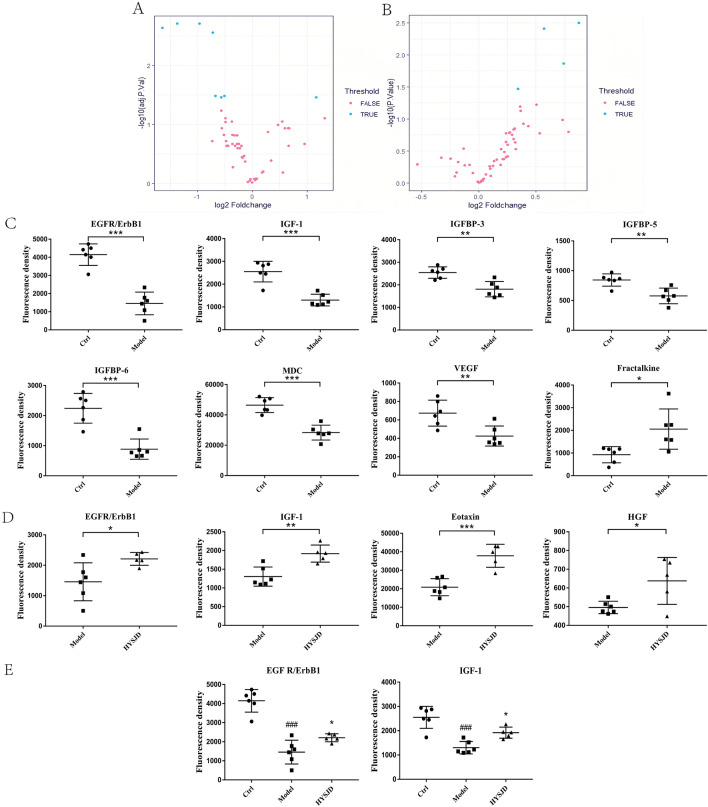
Identification of differentially expressed proteins in wound skin from the Ctrl, Model, and HYSJD mice using the growth factor and chemokine antibody array. **A**, **B** Volcano plot of the protein expression profiles in wound skin. **A** Model mice versus Ctrl mice; **B** HYSJD mice versus Model mice. The log2 (fold change) value of the differential protein expression levels was taken as the abscissa, and the negative logarithm-log10 (value) of the significance test values between the two groups was taken as the ordinate. Blue spots represent up-and down-regulated ≥ 1.2 or < 0.83-fold and p-value < 0.05 in the Model mice versus the Ctrl mice or the HYSJD mice versus the Model mice. **C** Eight differentially expressed proteins in wound skin between the Model mice versus the Ctrl mice. **D** Four differentially expressed proteins in wound skin between the HYSJD mice versus the Model mice. **E** Two differentially expressed proteins in wound skin among the Ctrl, Model, and HYSJD mice. *p-value < 0.05, **p-value < 0.01, ***p-value < 0.001

The growth factor and chemokine antibody array were used to measure the expression levels of 30 growth factors and 25 chemokines in the wound tissues of the control group, model group, and HYSJD treatment group 7 days after wound formation. Volcano plots of these differentially expressed proteins showed that, compared to the control group, the model group had 7 proteins that were down-regulated. These included insulin-like growth factor binding protein-6 (IGFBP-6), IGF-1, epidermal growth factor receptor (EGFR), Macrophage-derived chemokine (MDC), IGFBP-3, VEGF, IGFBP-5, and Fractalkine which were significantly down-regulated (Fold-change ≥ 1.2 or < 0.83, p-value < 0.05) (Fig. 3A, C); Four proteins in the HYSJD treatment group were up-regulated and included IGF-1, Eotaxin, EGFR, hepatocyte growth factor (HGF) (Fig. 3B, D). Of the three groups, i.e., the control group, model group, and HYSJD, the differentially expressed proteins were IGF-1 and EGFR (Fig. 3E). PCA identified three groups of differentially expressed proteins.

**Fig. 4:**
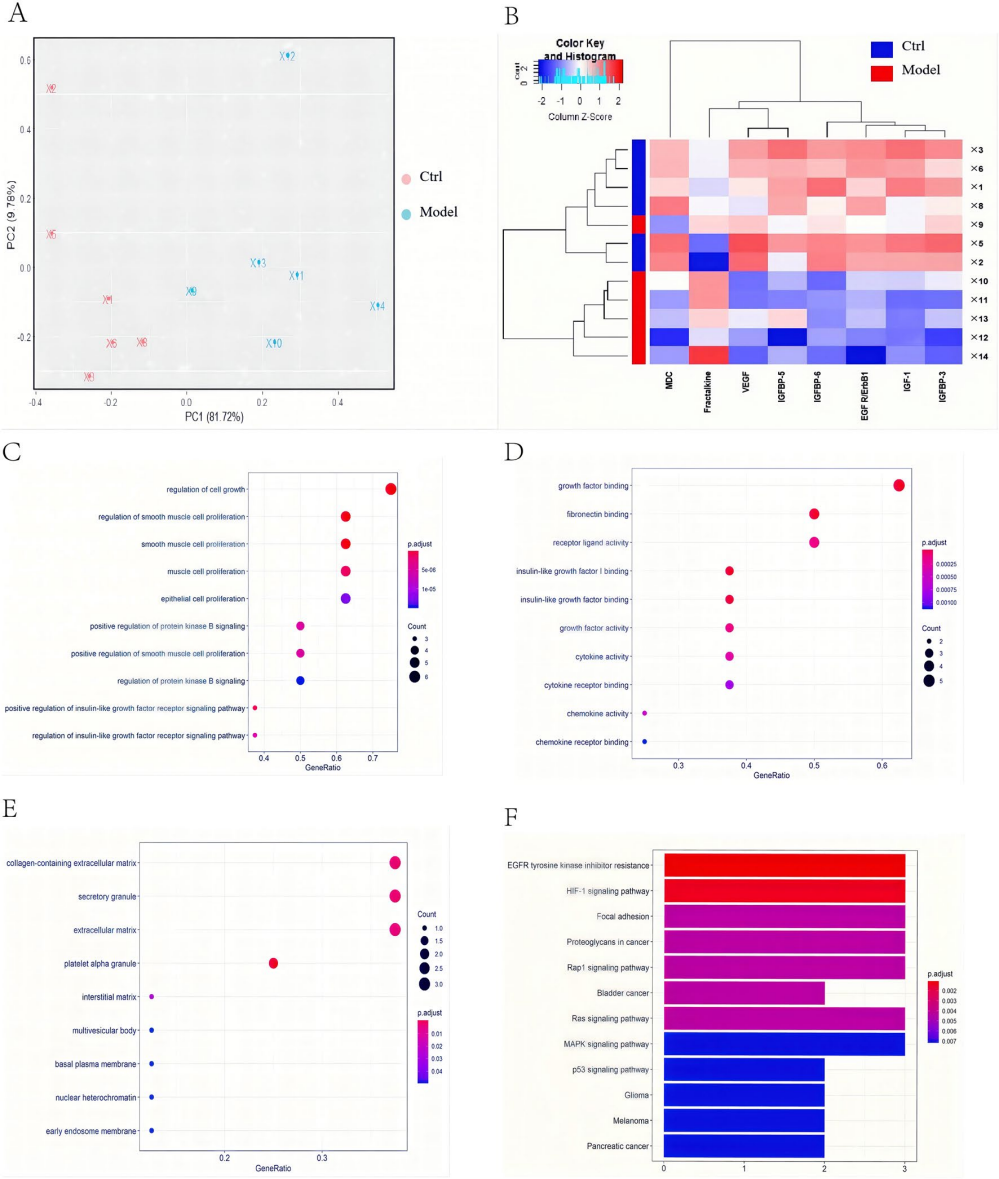
Differentially expressed protein levels between the Model and Ctrl mice. **A** PCA mapping of sixmodel mice and six Ctrl mouse samples. **B** Hierarchical cluster analysis of significantly different protein expression levels in wound skin. Each row represents a sample, and each column represents a protein. **C**–**E** GO enrichment analysis of significantly different protein expression levels with regards to **C** biological process, **D** molecular function, and **E** cellular component. **F** KEGG enrichment analysis of significantly enriched pathways in the differentially expressed proteins

**Fig. 5:**
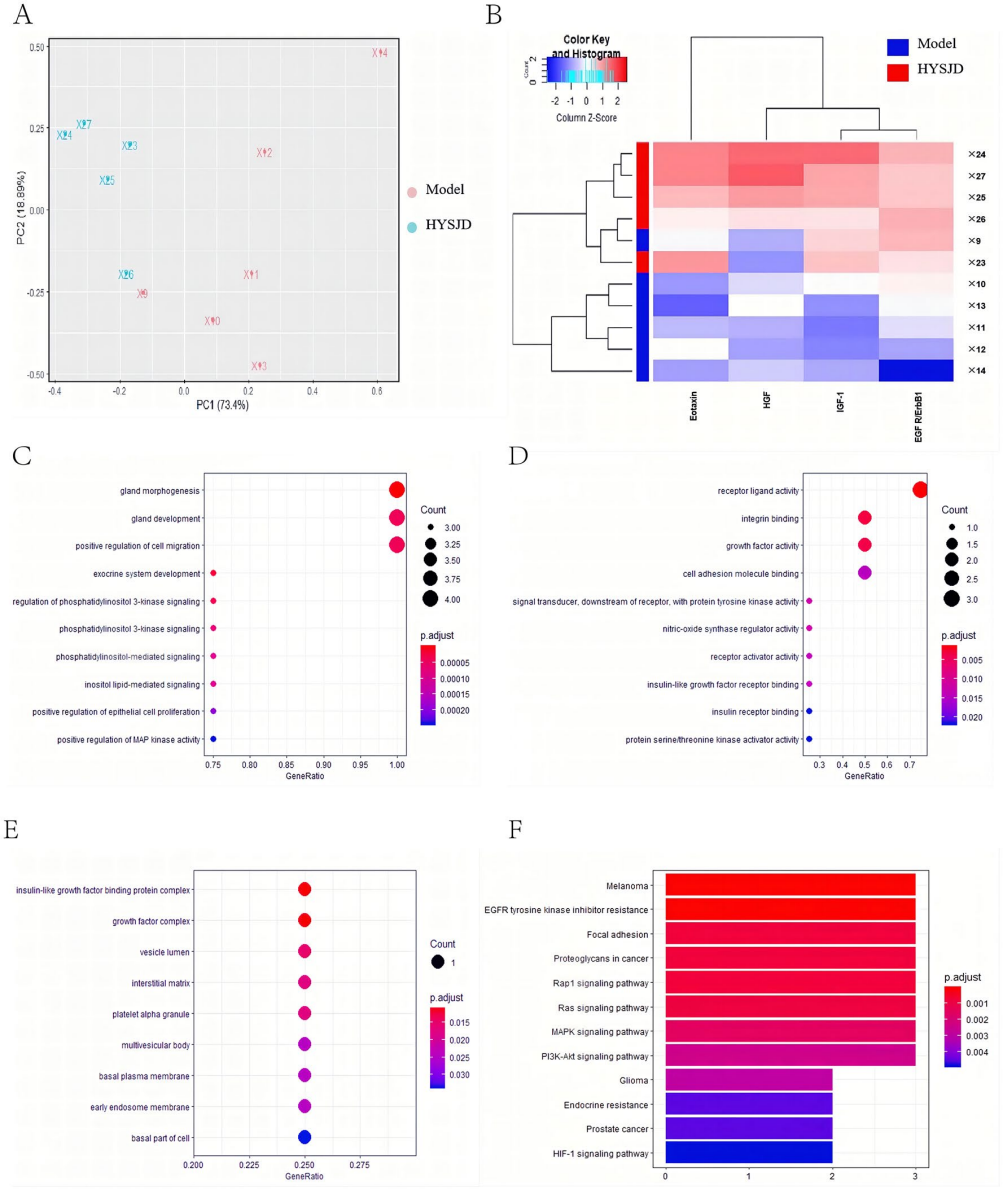
Differential protein expression levels between the Model and HYSJD treated mice. **A** PCA mapping of six model mice and five HYSJD mouse samples. **B** Hierarchical cluster analysis of significantly different protein expression levels in wound skin. Each row represents a sample, and each column represents a protein. **C**–**E** GO enrichment analysis of significantly different protein expression levels regarding **C** biological process, **D** molecular function, and **E** cellular component. **F** KEGG enrichment analysis of significantly enriched pathways in the differentially expressed proteins

Comparing the control group with the model group, the graphs for the first two principal components (PC1 and PC2) of each sample showed that the total variability accounted was 81.72% and 9.78%, respectively (Fig. 4A). This suggested that for the mice in the control group and the model, there were indeed differences in protein expression patterns between the groups of mice. Comparing the model group with the HYSJD group, the curves for the first two principal components (PC1 and PC2) of each sample showed that the total variability was 73.4% and 18.89%, respectively (Fig. 5A). This indicated that the model group and the HYSJD group had significantly different protein expression patterns. Hierarchical clustering was used to cluster the control and model group (Fig. 4B), as well as the model and HYSJD group (Fig. 5B) based on the differentially expressed proteins. The control and model group samples, as well as the model and HYSJD group were clearly divided into two main clusters. This indicated that the groups could be distinguished by the differential proteins and unsupervised clustering.

Using GO and KEGG enrichment analysis, the enriched pathways derived from the differentially expressed proteins were then studied. GO enrichment analysis showed that the differentially expressed proteins in the model group had 712 important functional terms for “biological process” (BP), 30 important functional terms for “molecular function (MF)” and 9 for the “cell component (CC)” category (Fig. 4C–E and Additional file 2: Table S2). The differential expressed proteins in the HYSJD treatment group had 794 important functional terms for “BP”, 37 important functional terms for “MF” and 9 for the “CC” category (Fig. 5C–E, and Additional file 3: Table S3).

KEGG enrichment analysis showed that the differentially expressed proteins in the model group were significantly enriched for 23 pathways (Fig. 4F and Additional file 4: Table S4). These enriched functional terms and signal pathways may explain the delayed wound healing in db/db mice. The differentially expressed proteins in the HYSJD treatment group were significantly enriched for 17 pathways (Fig. 5F and Additional file 5: Table S5). These enriched functional terms and signaling pathways may reflect the specific mechanism of HYSJD on the wound healing process.

### Serum EGFR levels

**Fig. 6:**
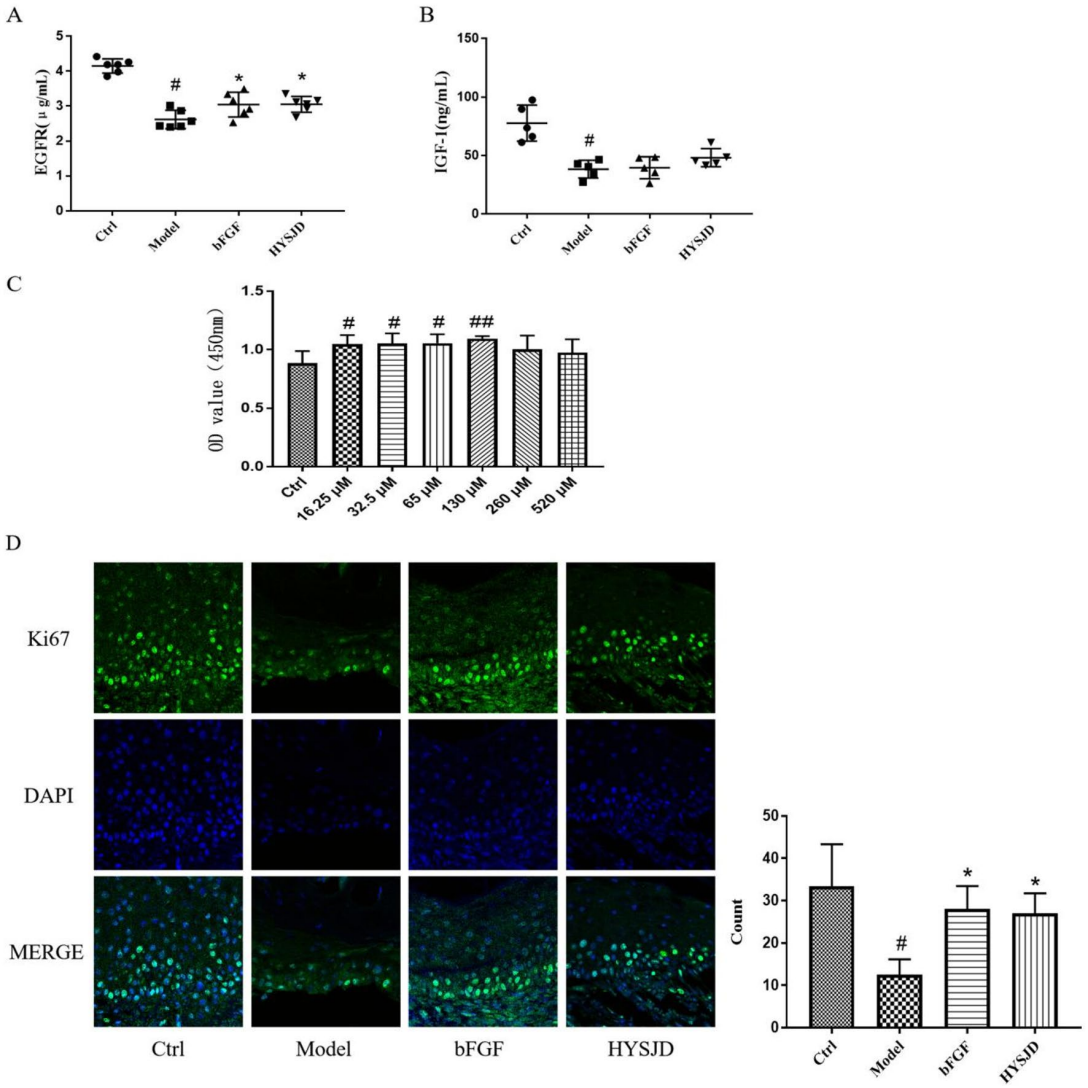
HYSJD facilitates cell proliferation in HaCaT cells by up-regulating the expression of EGFR. (A and B) EFGR and IGF-1 expression levels measured by ELISA. **A** Serum EGFR levels (*n* = 6 per group). **B** Serum IGF-1 levels (*n* = 6 per group). **C** Cell viability at different concentrations of HYSJD (*n* = 6). **D** Immunofluorescence staining of Ki67 (green) and quantitative results (*n* = 3 per group), nucleus (blue), ×400 . Data were expressed as mean ± SD, and significance was expressed as ^#^*P* < 0.05 and ^##^*P* < 0.01 vs Ctrl and *P < 0.05 vs Model

Compared with the control group, the serum EGFR levels in mice in the model group were reduced and the difference was statistically significant (*P* < 0.05). Compared to the model group, the serum EGFR levels in HYSJD treated mice were increased and the difference was statistically significant (*P* < 0.05) (Fig. 6A).

Compared to the control group, the IGF-1 levels in the model group were reduced (*P* < 0.05), while the IGF-1 levels in the HYSJD group were increased compared to the model group. However, the results were not statistically different (*P* > 0.05) (Fig. 6B). This demonstrated that HYSJD promotes the proliferation of epidermal cells by increasing the expression of EGFR to promote wound healing.

### Ki67 staining of wound epidermal basal cells

Ki67 staining of wound epidermal basal cells was performed to determine their proliferation ability. Compared to the control, the number of epidermal basal cells in the model group was significantly reduced (Fig. 6D) and the difference was statistically significant (*P* < 0.05). Compared to the model group, the bFGF group and the HYSJD group had an increased number of Ki67-positive cells in the epidermal basal layer and the difference was statistically significant (*P* < 0.05).

### Effect of HYSJD on the proliferation of Hacat cells

We used CCK-8 in vitro assays to observe the effect of HYSJD on the proliferation of human Hacat cells. Compared with the control group (PBS), 16.25 μM, 32.5 μM, 65 μM, and 130 μM HYSJD could significantly promote the proliferation of epidermal cells (Fig. 6C). The differences were statistically significant (*P* < 0.05).

### Effect of HYSJD on the PI3K/AKT pathway

**Fig. 7:**
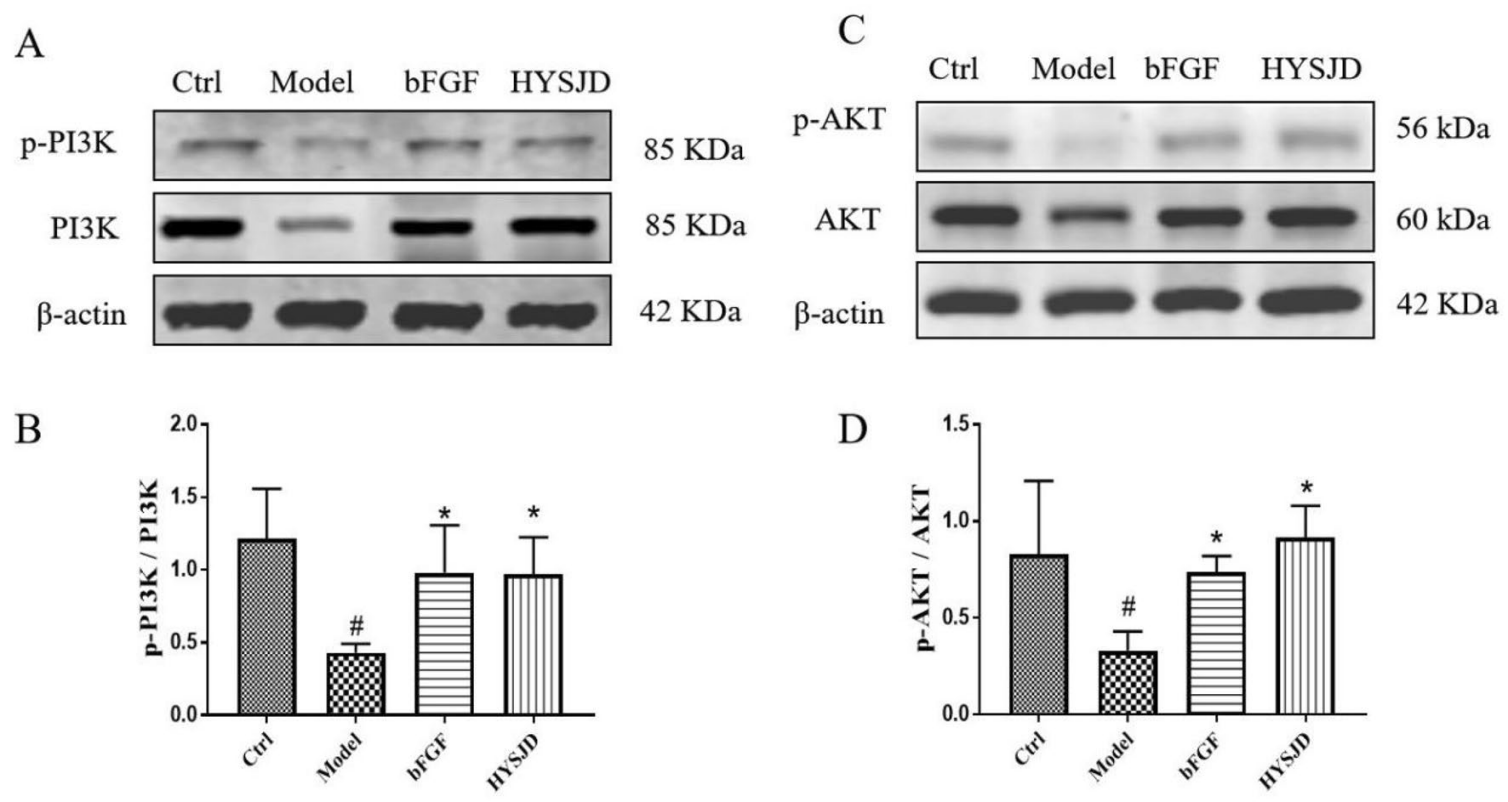
Treatment-activated PI3K/AKT signal pathway in db/db diabetic wound skin. **A** Representative western blotting results of p-PI3K and PI3K protein (*n* = 3). **B** Expression levels of p-PI3K and PI3K protein. **C** Representative western blotting results of p-AKT and AKT protein (*n* = 3). **D** Expression levels of p-AKT and AKT protein. Data were presented as mean ± SD, and significance was expressed as ^#^*P* < 0.05, compared to the Ctrl group. *P < 0.05 compared to the Model group

Western blot analysis was used to determine the effect of HYSJD on the EGFR-related PI3K/AKT signaling pathway. Compared to the control group, the expression levels of p-AKT and p-PI3K in the model group were reduced (Fig. 7A, B). Compared to the model group, the expression levels of p-AKT and p-PI3K in the bFGF group and HYSJD group were increased (Fig. 7C, D). This indicated that HYSJD activated the PI3K/AKT pathway.

## Discussion

A diabetic ulcer is a chronic skin ulcer accompanied by reduced expression of cytokines in the wound and dysfunction of wound repair cells such as keratinocytes. This study demonstrated that HYSJD promoted skin wound healing in db/db diabetic mice. HYSJD was found to promote the proliferation of wound epidermal cells. This was accompanied by the increase in EGFR expression levels in the wound and the activation of the PI3K/AKT signaling pathway.

Using the growth factor and chemokine antibody array, we found eight differentially expressed proteins in db/db wound model mice and db/m mice. In addition to the increased expression levels of fractalkine, the other seven proteins were all down-regulated, which included IGF-1, IGFBP-6, IGFBP-3, IGFBP-5, EGFR, VEGF, and MDC. These proteins induce changes in fibroblast growth, epidermal cell proliferation, and growth factor receptor binding. This in turn leads to delayed wound healing. The up-regulation of fractalkine can lead to increased inflammation [22] and increased cell apoptosis. IGF-1 is a polypeptide growth factor similar in molecular structure to insulin. Hyperglycemia inhibits the synthesis and release of IGF-1 [22]. IGF-1 has anti-inflammatory activity and can promote fibroblast proliferation, extracellular matrix synthesis, and angiogenesis to promote wound healing [23, 24]. VEGF is currently the most active factor in promoting angiogenesis, proliferation, migration, and differentiation of vascular endothelial cells into functional blood vessels to provide sufficient oxygen and nutrients for wound healing [25]. EGF after binding to EGFR, promotes the proliferation and migration of epidermal cells [26]. MDC is a cytokine that mediates the chemotactic movement of leukocytes, especially monocytes, macrophages, T lymphocytes (especially Th2 cells), and natural killer cells. After tissue injury, monocytes infiltrate the injury site and can differentiate into different macrophage phenotypes, such as M2 macrophages to promote wound repair [27]. The slow wound healing in diabetic mice may be related to the decreased chemotaxis of MDC on monocyte macrophages and decreased polarization of M2 macrophages. In summary, the up-regulation of fractalkine, down-regulation of IGF-1, IGFBP, VEGF, EGFR, and MDC lead to the failure of wound repair in db/db mice and delayed healing.

Diabetic chronic skin ulcers belong to the category of “*skin and external diseases*” in traditional Chinese medicine. Traditional Chinese medicine believes that insufficiency in the spleen and kidney, blood deficiency, and blood stasis play an important role in the onset of diabetic chronic skin ulcers. Insufficiency of the spleen and kidney leads to a lack of biochemical sources of qi and blood, and blood stasis affects the circulation of qi and blood to the wound area. If the wound lacks qi and blood, healing is reduced [28]. HYSJD has the effects of strengthening the spleen and replenishing qi, strengthening the kidney and warming yang, tonifying blood, and promoting blood circulation. It can improve chronic wounds with deficiency of spleen and kidney and qi deficiency and blood stasis to promote wound healing. By analyzing the components of HYSJD, we found that hypaconitine, neoline, calycosin, calycosin-7-glucoside, verbascose, formononetin, sinapine, etc. was abundant in the water extracts.

After oral administration of HYSJD, five absorbed components in the serum were detected by UPLC-MS/MS. These included formononetin, calycosin, hypaconitine, calycosin-7-glucoside, and sinapic acid. Whether HYSJD components could be absorbed into the blood is an important prerequisite for its pharmacological activity. Hence, the efficacy of oral HYSJD on the healing of diabetic chronic ulcers was likely attributable to the five components measured in the blood. Additional studies were needed to determine which component of HYSJD is essential for its efficacy.

Studies have reported that hypaconitine can activate mesenchymal precursor cells and promote skin stromal cells to produce higher levels of growth factors and promote skin wound healing [29]. Calycosin, a cardiovascular protective isoflavone, can promote angiogenesis through the PI3K/AKT pathway [30]. Calycosin-7-glucoside is a calycosin derivative compound derived from *Astragli Radix*. It can induce myocardial ischemia–reperfusion injury and bacterial endotoxin induction. Vascular cell damage has a protective effect to improve myocardial blood supply, reduce inflammation and oxidative damage [31]. Formononetin is a type of phytoestrogen derived from the root of *Astragli Radix*. It is used as a blood enhancer and improves blood microcirculation in complementary and alternative medicine. It can induce phosphorylation of ERK in human umbilical vein endothelial cells, increase growth factors such as TGF-β1, VEGF, PDGF, and bFGF to promote wound healing [32].

The four differentially expressed proteins in the HYSJD treatment group and the model group were all up-regulated and included IGF-1, EGFR, Eotaxin, and HGF. HGF is secreted by fibroblasts and endothelial cells and mediates cell migration, proliferation, survival, regulates angiogenesis, matrix deposition, and degradation, promotes wound re-epithelization, and reduces scar formation [33]. We found that expression levels of EGFR and IGF-1 in the wound of model mice were decreased, but increased after HYSJD treatment. Hence, we believe that the effect of HYSJD in promoting wound healing in db/db mice was closely related to the increase of EGFR and IGF-1. However, ELISA assays demonstrated that EGFR in the serum was significantly increased, and not IGF-1. This suggested that the mechanism of HYSJD in wound healing was through EGFR.

HYSJD was observed to promote the proliferation of wound keratinocytes (Ki67) and the regeneration of granulation tissue. Ki67 is present in all active phases of the cell cycle (G1, S, G2, and M phases). Ki67 protein expression is significantly increased in S phase cells [34], but not in quiescent cells (G0 phase) [35]. Ki67 protein staining indicated that the number of wound keratinocytes in the proliferation stage was increased. In vitro experiments further confirmed that HYSJD could promote the proliferation of Hacat cells. Keratinocytes are the main cell component of the epidermis and are responsible for re-epithelialization. Keratinocytes migrate to the newly formed granulation tissue and re-epithelialize. This process includes activation of membrane-related kinases to increase the permeability of the cell membrane to calcium ions, which causes the intracellular tension filaments to contract and move in the direction of the wound [36]. Re-epithelialization is an important part of wound healing and a decisive indicator of successful wound healing. In all types of chronic wounds, the epithelialization process is impaired [37]. The EGF/EGFR signaling pathway is one of the important pathways that promote the proliferation and migration of epidermal cells [38]. In EGFR-deficient skin, the migration of epithelial cells to the wound is impaired [39]. PI3K/AKT and MAPK/ERK are important signaling molecules that are downstream of EGFR crucial for wound healing.

We next investigated the mechanism of HYSJD on wound healing. KEGG and GO analysis were performed to identify the enriched biological process of the differential expressed proteins after HYSJD treatment. We found that the PI3K/AKT signaling pathway was highly enriched. PI3K/AKT signaling pathway has been shown to play a key role in skin development and wound healing [40, 41]. We found that after HYSJD treatment, the expression levels of p-PI3K and p-AKT were increased in db/db mice wounds.

Our study demonstrated that HYSJD could enhance the proliferation of keratinocytes in db/db diabetic mice wounds. This was accompanied by the increase in EGFR expression levels in the wound and the activation of the PI3K/AKT signalling pathway.

## Conclusion

We found that the main components of HYSJD were flavonoids, terpenes, alkaloids, phenylpropanoids, and carbohydrates. After oral administration of HYSJD, formononetin, calycosin, hypaconitine, calycosin-7-glucoside, and sinapic acid were detected in the serum. HYSJD was found to promote the proliferation of epidermal cells in chronic diabetic wounds. This was accompanied by the increase in EGFR expression levels in the wounds and the activation of PI3K/AKT signaling pathway.

## Supplementary Information

**Additional file 1: Table S1.** Gene list of the Growth factor and chemokine antibody array.

**Additional file 2: Table S2.** GO analysis of the differentially-expressed proteins between the control and model group.

**Additional file 3: Table S3.** GO analysis of the differentially-expressed proteins between the HYSJD and model group.

**Additional file 4: Table S4.** KEGG analysis of the differentially-expressed proteins between the control and model group.

**Additional file 5: Table S5.** KEGG analysis of the differentially-expressed proteins between the HYSJD and model group.

## Data Availability

The datasets used and/or analyzed during the current study are available from the corresponding author on reasonable request.

## Abbreviations

HYSJD: Huiyang Shengji decoction
UPLC-MS/MS: Ultra-performance liquid chromatography was combined with tandem mass spectrometry
GFs: Growth factors
PDGF: Platelet-derived GF
VEGF: Vascular endothelial GF
EGF: Epidermal GF
FGF: Fibroblast GF
TGF: Transforming GF
KGF: Keratinocyte GF
IGF: Insulin-like GF
bFGF: Basic fibroblast growth factor
PCA: Principal component analysis
ELISA: Enzyme-linked immunosorbent assay
ANOVA: One-way analysis of variance
IGFBP-6: Insulin-like growth factor binding protein-6
EGFR: Epidermal growth factor receptor
MDC: Macrophage-derived chemokine
HGF: Hepatocyte growth factor
BP: Biological process
MF: Molecular function
CC: Cell component
