# Supplementary material for: Huiyang Shengji decoction promotes wound healing in diabetic mice by activating the EGFR/PI3K/ATK pathway
Source: Chin Med. 2021 Nov 2;16:111. doi: 10.1186/s13020-021-00497-0 (PMC8565039; doi:10.1186/s13020-021-00497-0)
Supplement: Supplementary file 1 — Additional file 1: Table S1. Gene list of the Growth factor and chemokine antibody array. [file 13020_2021_497_MOESM1_ESM.doc]

| **Table S1：Gene list of the Growth factor and chemokine antibody array** | | |
| --- | --- | --- |
| Number | Growth factor | chemokine |
| 1 | Amphiregulin /AR | CCL21 |
| 2 | bFGF | BLC |
| 3 | b-NGF | CTACK |
| 4 | EGF | CXCL16 |
| 5 | EGF R/ErbB1 | Eotaxin |
| 6 | GCSF | Eotaxin-2 |
| 7 | GM-CSF | Fractalkine |
| 8 | FGF-7 | I-TAC |
| 9 | HGF | KC |
| 10 | HGF R | LIX |
| 11 | IGFBP-2 | MCP-1 |
| 12 | IGFBP-3 | MCP-5 |
| 13 | IGFBP-5 | MDC |
| 14 | IGFBP-6 | MIG |
| 15 | IGF-I | MIP-1a |
| 16 | IGF-I sR | MIP-1g |
| 17 | IGF-II | MIP-2 |
| 18 | IL-2 | MIP-3a |
| 19 | IL-7 | MIP-3b |
| 20 | M-CSF | PF4 |
| 21 | PDGF-AA | RANTES |
| 22 | PDGF-BB | SDF-1a |
| 23 | PlGF-2 | TARC |
| 24 | SCF | TCA-3 |
| 25 | TGF-beta1 | TECK |
| 26 | VEGF |  |
| 27 | VEGF R1 |  |
| 28 | VEGF R2 |  |
| 29 | VEGF R3 |  |
| 30 | VEGF-D |  |
