# Supplementary material for: Huiyang Shengji decoction promotes wound healing in diabetic mice by activating the EGFR/PI3K/ATK pathway
Source: Chin Med. 2021 Nov 2;16:111. doi: 10.1186/s13020-021-00497-0 (PMC8565039; doi:10.1186/s13020-021-00497-0)
Supplement: Supplementary file 2 — Additional file 2: Table S2. GO analysis of the differentially-expressed proteins between the control and model group. [file 13020_2021_497_MOESM2_ESM.doc]

|  | **Table S2: GO analysis of the differentially-expressed proteins between the control and model group** | | | | | | | |
| --- | --- | --- | --- | --- | --- | --- | --- | --- |
|  | Gene Ontology Classification | ID | Description | GeneRatio | pvalue | p.adjust | geneID | Count |
| 1 | Biological Process | GO:0001558 | regulation of cell growth | 0.75 | 0.000000001 | 0.000000437 | 16012/16000/13649/16009/22339/16011 | 6 |
| 2 | Biological Process | GO:0048660 | regulation of smooth muscle cell proliferation | 0.625 | 0.000000001 | 0.000000437 | 16000/13649/16009/22339/16011 | 5 |
| 3 | Biological Process | GO:0048659 | smooth muscle cell proliferation | 0.625 | 0.000000001 | 0.000000437 | 16000/13649/16009/22339/16011 | 5 |
| 4 | Biological Process | GO:0033002 | muscle cell proliferation | 0.625 | 0.000000005 | 0.000001232 | 16000/13649/16009/22339/16011 | 5 |
| 5 | Biological Process | GO:0050673 | epithelial cell proliferation | 0.625 | 0.000000092 | 0.000012350 | 16000/13649/16009/22339/16011 | 5 |
| 6 | Biological Process | GO:0051897 | positive regulation of protein kinase B signaling | 0.5 | 0.000000023 | 0.000004693 | 16000/13649/22339/16011 | 4 |
| 7 | Biological Process | GO:0048661 | positive regulation of smooth muscle cell proliferation | 0.5 | 0.000000027 | 0.000004707 | 16000/13649/22339/16011 | 4 |
| 8 | Biological Process | GO:0051896 | regulation of protein kinase B signaling | 0.5 | 0.000000121 | 0.000014636 | 16000/13649/22339/16011 | 4 |
| 9 | Biological Process | GO:0043491 | protein kinase B signaling | 0.5 | 0.000000243 | 0.000024583 | 16000/13649/22339/16011 | 4 |
| 10 | Biological Process | GO:0048771 | tissue remodeling | 0.5 | 0.000000277 | 0.000025860 | 16000/13649/22339/16011 | 4 |
| 11 | Biological Process | GO:0001649 | osteoblast differentiation | 0.5 | 0.000000308 | 0.000026690 | 16000/16009/22339/16011 | 4 |
| 12 | Biological Process | GO:0070372 | regulation of ERK1 and ERK2 cascade | 0.5 | 0.000002672 | 0.000190672 | 16000/13649/20299/22339 | 4 |
| 13 | Biological Process | GO:0070371 | ERK1 and ERK2 cascade | 0.5 | 0.000003300 | 0.000210653 | 16000/13649/20299/22339 | 4 |
| 14 | Biological Process | GO:0001503 | ossification | 0.5 | 0.000004163 | 0.000252487 | 16000/16009/22339/16011 | 4 |
| 15 | Biological Process | GO:0048732 | gland development | 0.5 | 0.000010482 | 0.000508593 | 16000/13649/22339/16011 | 4 |
| 16 | Biological Process | GO:0010608 | posttranscriptional regulation of gene expression | 0.5 | 0.000012474 | 0.000537495 | 16000/13649/22339/16011 | 4 |
| 17 | Biological Process | GO:0043568 | positive regulation of insulin-like growth factor receptor signaling pathway | 0.375 | 0.000000004 | 0.000001232 | 16000/16009/16011 | 3 |
| 18 | Biological Process | GO:0043567 | regulation of insulin-like growth factor receptor signaling pathway | 0.375 | 0.000000041 | 0.000006233 | 16000/16009/16011 | 3 |
| 19 | Biological Process | GO:0048009 | insulin-like growth factor receptor signaling pathway | 0.375 | 0.000000145 | 0.000016032 | 16000/16009/16011 | 3 |
| 20 | Biological Process | GO:0048286 | lung alveolus development | 0.375 | 0.000000863 | 0.000069757 | 16000/22339/16011 | 3 |
| 21 | Biological Process | GO:0014910 | regulation of smooth muscle cell migration | 0.375 | 0.000002518 | 0.000190672 | 16000/16009/16011 | 3 |
| 22 | Biological Process | GO:0014909 | smooth muscle cell migration | 0.375 | 0.000003207 | 0.000210653 | 16000/16009/16011 | 3 |
| 23 | Biological Process | GO:0014812 | muscle cell migration | 0.375 | 0.000004937 | 0.000285190 | 16000/16009/16011 | 3 |
| 24 | Biological Process | GO:0010906 | regulation of glucose metabolic process | 0.375 | 0.000006664 | 0.000360331 | 16000/16009/16011 | 3 |
| 25 | Biological Process | GO:0071621 | granulocyte chemotaxis | 0.375 | 0.000007947 | 0.000401669 | 20299/22339/14825 | 3 |
| 26 | Biological Process | GO:0045667 | regulation of osteoblast differentiation | 0.375 | 0.000011722 | 0.000537495 | 16000/22339/16011 | 3 |
| 27 | Biological Process | GO:0010675 | regulation of cellular carbohydrate metabolic process | 0.375 | 0.000013022 | 0.000537495 | 16000/16009/16011 | 3 |
| 28 | Biological Process | GO:0097530 | granulocyte migration | 0.375 | 0.000013022 | 0.000537495 | 20299/22339/14825 | 3 |
| 29 | Biological Process | GO:0022612 | gland morphogenesis | 0.375 | 0.000013293 | 0.000537495 | 16000/13649/16011 | 3 |
| 30 | Biological Process | GO:0030879 | mammary gland development | 0.375 | 0.000018829 | 0.000713744 | 16000/22339/16011 | 3 |
| 31 | Biological Process | GO:0006109 | regulation of carbohydrate metabolic process | 0.375 | 0.000021712 | 0.000798073 | 16000/16009/16011 | 3 |
| 32 | Biological Process | GO:0035051 | cardiocyte differentiation | 0.375 | 0.000024872 | 0.000861980 | 16000/13649/22339 | 3 |
| 33 | Biological Process | GO:0006006 | glucose metabolic process | 0.375 | 0.000029230 | 0.000914541 | 16000/16009/16011 | 3 |
| 34 | Biological Process | GO:0030307 | positive regulation of cell growth | 0.375 | 0.000029692 | 0.000914541 | 16000/13649/22339 | 3 |
| 35 | Biological Process | GO:2000377 | regulation of reactive oxygen species metabolic process | 0.375 | 0.000030158 | 0.000914541 | 16000/13649/14825 | 3 |
| 36 | Biological Process | GO:0050679 | positive regulation of epithelial cell proliferation | 0.375 | 0.000031586 | 0.000934488 | 16000/13649/22339 | 3 |
| 37 | Biological Process | GO:0097529 | myeloid leukocyte migration | 0.375 | 0.000033058 | 0.000946788 | 20299/22339/14825 | 3 |
| 38 | Biological Process | GO:0030595 | leukocyte chemotaxis | 0.375 | 0.000039398 | 0.001086135 | 20299/22339/14825 | 3 |
| 39 | Biological Process | GO:0030324 | lung development | 0.375 | 0.000043442 | 0.001143629 | 16000/22339/16011 | 3 |
| 40 | Biological Process | GO:0019318 | hexose metabolic process | 0.375 | 0.000044645 | 0.001143629 | 16000/16009/16011 | 3 |
| 41 | Biological Process | GO:0043406 | positive regulation of MAP kinase activity | 0.375 | 0.000044645 | 0.001143629 | 16000/13649/22339 | 3 |
| 42 | Biological Process | GO:0030323 | respiratory tube development | 0.375 | 0.000045255 | 0.001143629 | 16000/22339/16011 | 3 |
| 43 | Biological Process | GO:0030278 | regulation of ossification | 0.375 | 0.000049030 | 0.001213733 | 16000/22339/16011 | 3 |
| 44 | Biological Process | GO:0051054 | positive regulation of DNA metabolic process | 0.375 | 0.000056477 | 0.001317437 | 13649/16009/22339 | 3 |
| 45 | Biological Process | GO:0032147 | activation of protein kinase activity | 0.375 | 0.000061583 | 0.001383341 | 16000/13649/22339 | 3 |
| 46 | Biological Process | GO:0005996 | monosaccharide metabolic process | 0.375 | 0.000065410 | 0.001391978 | 16000/16009/16011 | 3 |
| 47 | Biological Process | GO:0060541 | respiratory system development | 0.375 | 0.000065410 | 0.001391978 | 16000/22339/16011 | 3 |
| 48 | Biological Process | GO:0070374 | positive regulation of ERK1 and ERK2 cascade | 0.375 | 0.000065410 | 0.001391978 | 13649/20299/22339 | 3 |
| 49 | Biological Process | GO:0050730 | regulation of peptidyl-tyrosine phosphorylation | 0.375 | 0.000068582 | 0.001434313 | 16000/13649/22339 | 3 |
| 50 | Biological Process | GO:0071902 | positive regulation of protein serine/threonine kinase activity | 0.375 | 0.000086900 | 0.001746793 | 16000/13649/22339 | 3 |
| 51 | Biological Process | GO:0044262 | cellular carbohydrate metabolic process | 0.375 | 0.000087844 | 0.001746793 | 16000/16009/16011 | 3 |
| 52 | Biological Process | GO:0072593 | reactive oxygen species metabolic process | 0.375 | 0.000091688 | 0.001793826 | 16000/13649/14825 | 3 |
| 53 | Biological Process | GO:0032103 | positive regulation of response to external stimulus | 0.375 | 0.000104939 | 0.002009180 | 13649/22339/14825 | 3 |
| 54 | Biological Process | GO:0043405 | regulation of MAP kinase activity | 0.375 | 0.000106008 | 0.002009180 | 16000/13649/22339 | 3 |
| 55 | Biological Process | GO:0060326 | cell chemotaxis | 0.375 | 0.000108166 | 0.002018542 | 20299/22339/14825 | 3 |
| 56 | Biological Process | GO:1903829 | positive regulation of cellular protein localization | 0.375 | 0.000112568 | 0.002068863 | 16000/13649/22339 | 3 |
| 57 | Biological Process | GO:0051146 | striated muscle cell differentiation | 0.375 | 0.000114812 | 0.002078619 | 16000/22339/16011 | 3 |
| 58 | Biological Process | GO:0045927 | positive regulation of growth | 0.375 | 0.000119389 | 0.002126552 | 16000/13649/22339 | 3 |
| 59 | Biological Process | GO:0018108 | peptidyl-tyrosine phosphorylation | 0.375 | 0.000124083 | 0.002150183 | 16000/13649/22339 | 3 |
| 60 | Biological Process | GO:0018212 | peptidyl-tyrosine modification | 0.375 | 0.000127682 | 0.002181386 | 16000/13649/22339 | 3 |
| 61 | Biological Process | GO:0050900 | leukocyte migration | 0.375 | 0.000145382 | 0.002287119 | 20299/22339/14825 | 3 |
| 62 | Biological Process | GO:0062012 | regulation of small molecule metabolic process | 0.375 | 0.000154803 | 0.002376918 | 16000/16009/16011 | 3 |
| 63 | Biological Process | GO:0050678 | regulation of epithelial cell proliferation | 0.375 | 0.000177817 | 0.002624151 | 16000/13649/22339 | 3 |
| 64 | Biological Process | GO:0042692 | muscle cell differentiation | 0.375 | 0.000232102 | 0.003199313 | 16000/22339/16011 | 3 |
| 65 | Biological Process | GO:0034655 | nucleobase-containing compound catabolic process | 0.375 | 0.000267756 | 0.003418826 | 16000/16009/22339 | 3 |
| 66 | Biological Process | GO:0071900 | regulation of protein serine/threonine kinase activity | 0.375 | 0.000302528 | 0.003822564 | 16000/13649/22339 | 3 |
| 67 | Biological Process | GO:0019932 | second-messenger-mediated signaling | 0.375 | 0.000317658 | 0.003972356 | 16000/13649/22339 | 3 |
| 68 | Biological Process | GO:0051052 | regulation of DNA metabolic process | 0.375 | 0.000324289 | 0.003973365 | 13649/16009/22339 | 3 |
| 69 | Biological Process | GO:0045860 | positive regulation of protein kinase activity | 0.375 | 0.000328760 | 0.003987864 | 16000/13649/22339 | 3 |
| 70 | Biological Process | GO:0044270 | cellular nitrogen compound catabolic process | 0.375 | 0.000347045 | 0.004138432 | 16000/16009/22339 | 3 |
| 71 | Biological Process | GO:0046700 | heterocycle catabolic process | 0.375 | 0.000361183 | 0.004176252 | 16000/16009/22339 | 3 |
| 72 | Biological Process | GO:0048608 | reproductive structure development | 0.375 | 0.000363575 | 0.004176252 | 16000/13649/22339 | 3 |
| 73 | Biological Process | GO:0061458 | reproductive system development | 0.375 | 0.000373247 | 0.004231295 | 16000/13649/22339 | 3 |
| 74 | Biological Process | GO:0019439 | aromatic compound catabolic process | 0.375 | 0.000395616 | 0.004418646 | 16000/16009/22339 | 3 |
| 75 | Biological Process | GO:0060537 | muscle tissue development | 0.375 | 0.000400702 | 0.004418646 | 16000/22339/16011 | 3 |
| 76 | Biological Process | GO:0033674 | positive regulation of kinase activity | 0.375 | 0.000418837 | 0.004456570 | 16000/13649/22339 | 3 |
| 77 | Biological Process | GO:0071407 | cellular response to organic cyclic compound | 0.375 | 0.000467894 | 0.004684187 | 16000/13649/16011 | 3 |
| 78 | Biological Process | GO:1901361 | organic cyclic compound catabolic process | 0.375 | 0.000473564 | 0.004684187 | 16000/16009/22339 | 3 |
| 79 | Biological Process | GO:0001701 | in utero embryonic development | 0.375 | 0.000493758 | 0.004791427 | 16000/13649/22339 | 3 |
| 80 | Biological Process | GO:0010749 | regulation of nitric oxide mediated signal transduction | 0.25 | 0.000006832 | 0.000360331 | 13649/22339 | 2 |
| 81 | Biological Process | GO:0032930 | positive regulation of superoxide anion generation | 0.25 | 0.000017681 | 0.000691824 | 13649/14825 | 2 |
| 82 | Biological Process | GO:0014912 | negative regulation of smooth muscle cell migration | 0.25 | 0.000023872 | 0.000851668 | 16009/16011 | 2 |
| 83 | Biological Process | GO:0032928 | regulation of superoxide anion generation | 0.25 | 0.000026141 | 0.000880806 | 13649/14825 | 2 |
| 84 | Biological Process | GO:0044342 | type B pancreatic cell proliferation | 0.25 | 0.000028513 | 0.000914541 | 16009/16011 | 2 |
| 85 | Biological Process | GO:0007263 | nitric oxide mediated signal transduction | 0.25 | 0.000033563 | 0.000946788 | 13649/22339 | 2 |
| 86 | Biological Process | GO:0042554 | superoxide anion generation | 0.25 | 0.000051169 | 0.001241369 | 13649/14825 | 2 |
| 87 | Biological Process | GO:0090322 | regulation of superoxide metabolic process | 0.25 | 0.000054461 | 0.001295324 | 13649/14825 | 2 |
| 88 | Biological Process | GO:1904707 | positive regulation of vascular smooth muscle cell proliferation | 0.25 | 0.000057855 | 0.001324118 | 16000/16011 | 2 |
| 89 | Biological Process | GO:0038083 | peptidyl-tyrosine autophosphorylation | 0.25 | 0.000084464 | 0.001736513 | 13649/22339 | 2 |
| 90 | Biological Process | GO:0045124 | regulation of bone resorption | 0.25 | 0.000120966 | 0.002126552 | 13649/22339 | 2 |
| 91 | Biological Process | GO:0001974 | blood vessel remodeling | 0.25 | 0.000131104 | 0.002208747 | 16000/22339 | 2 |
| 92 | Biological Process | GO:1904705 | regulation of vascular smooth muscle cell proliferation | 0.25 | 0.000136325 | 0.002234628 | 16000/16011 | 2 |
| 93 | Biological Process | GO:1990874 | vascular smooth muscle cell proliferation | 0.25 | 0.000136325 | 0.002234628 | 16000/16011 | 2 |
| 94 | Biological Process | GO:0048662 | negative regulation of smooth muscle cell proliferation | 0.25 | 0.000141647 | 0.002260759 | 16009/16011 | 2 |
| 95 | Biological Process | GO:2000648 | positive regulation of stem cell proliferation | 0.25 | 0.000141647 | 0.002260759 | 22339/14825 | 2 |
| 96 | Biological Process | GO:0035272 | exocrine system development | 0.25 | 0.000147069 | 0.002287119 | 16000/13649 | 2 |
| 97 | Biological Process | GO:0043536 | positive regulation of blood vessel endothelial cell migration | 0.25 | 0.000158217 | 0.002398970 | 16000/22339 | 2 |
| 98 | Biological Process | GO:0046850 | regulation of bone remodeling | 0.25 | 0.000175695 | 0.002624151 | 13649/22339 | 2 |
| 99 | Biological Process | GO:0014911 | positive regulation of smooth muscle cell migration | 0.25 | 0.000181722 | 0.002624151 | 16000/16011 | 2 |
| 100 | Biological Process | GO:1905207 | regulation of cardiocyte differentiation | 0.25 | 0.000181722 | 0.002624151 | 16000/13649 | 2 |
| 101 | Biological Process | GO:0006801 | superoxide metabolic process | 0.25 | 0.000194078 | 0.002737401 | 13649/14825 | 2 |
| 102 | Biological Process | GO:0070098 | chemokine-mediated signaling pathway | 0.25 | 0.000194078 | 0.002737401 | 20299/14825 | 2 |
| 103 | Biological Process | GO:2000179 | positive regulation of neural precursor cell proliferation | 0.25 | 0.000219995 | 0.003067285 | 16000/22339 | 2 |
| 104 | Biological Process | GO:0045669 | positive regulation of osteoblast differentiation | 0.25 | 0.000240485 | 0.003241209 | 16000/22339 | 2 |
| 105 | Biological Process | GO:0048146 | positive regulation of fibroblast proliferation | 0.25 | 0.000240485 | 0.003241209 | 16000/13649 | 2 |
| 106 | Biological Process | GO:1990868 | response to chemokine | 0.25 | 0.000247516 | 0.003263443 | 20299/14825 | 2 |
| 107 | Biological Process | GO:1990869 | cellular response to chemokine | 0.25 | 0.000247516 | 0.003263443 | 20299/14825 | 2 |
| 108 | Biological Process | GO:0045453 | bone resorption | 0.25 | 0.000254646 | 0.003321356 | 13649/22339 | 2 |
| 109 | Biological Process | GO:0014743 | regulation of muscle hypertrophy | 0.25 | 0.000261877 | 0.003379326 | 16000/16011 | 2 |
| 110 | Biological Process | GO:0043154 | negative regulation of cysteine-type endopeptidase activity involved in apoptotic process | 0.25 | 0.000323314 | 0.003973365 | 16000/22339 | 2 |
| 111 | Biological Process | GO:0034103 | regulation of tissue remodeling | 0.25 | 0.000347997 | 0.004138432 | 13649/22339 | 2 |
| 112 | Biological Process | GO:0072091 | regulation of stem cell proliferation | 0.25 | 0.000356423 | 0.004176252 | 22339/14825 | 2 |
| 113 | Biological Process | GO:2000117 | negative regulation of cysteine-type endopeptidase activity | 0.25 | 0.000364949 | 0.004176252 | 16000/22339 | 2 |
| 114 | Biological Process | GO:0001938 | positive regulation of endothelial cell proliferation | 0.25 | 0.000400043 | 0.004418646 | 16000/22339 | 2 |
| 115 | Biological Process | GO:0014066 | regulation of phosphatidylinositol 3-kinase signaling | 0.25 | 0.000409065 | 0.004430316 | 16000/13649 | 2 |
| 116 | Biological Process | GO:0043535 | regulation of blood vessel endothelial cell migration | 0.25 | 0.000409065 | 0.004430316 | 16000/22339 | 2 |
| 117 | Biological Process | GO:0010660 | regulation of muscle cell apoptotic process | 0.25 | 0.000418185 | 0.004456570 | 16000/16009 | 2 |
| 118 | Biological Process | GO:0045445 | myoblast differentiation | 0.25 | 0.000446140 | 0.004684187 | 16000/16009 | 2 |
| 119 | Biological Process | GO:0010595 | positive regulation of endothelial cell migration | 0.25 | 0.000455656 | 0.004684187 | 16000/22339 | 2 |
| 120 | Biological Process | GO:0043502 | regulation of muscle adaptation | 0.25 | 0.000455656 | 0.004684187 | 16000/16011 | 2 |
| 121 | Biological Process | GO:0010657 | muscle cell apoptotic process | 0.25 | 0.000465270 | 0.004684187 | 16000/16009 | 2 |
| 122 | Biological Process | GO:0046849 | bone remodeling | 0.25 | 0.000465270 | 0.004684187 | 13649/22339 | 2 |
| 123 | Biological Process | GO:0055013 | cardiac muscle cell development | 0.25 | 0.000474983 | 0.004684187 | 16000/22339 | 2 |
| 124 | Biological Process | GO:2001237 | negative regulation of extrinsic apoptotic signaling pathway | 0.25 | 0.000474983 | 0.004684187 | 16000/22339 | 2 |
| 125 | Biological Process | GO:0045778 | positive regulation of ossification | 0.25 | 0.000484795 | 0.004742394 | 16000/22339 | 2 |
| 126 | Biological Process | GO:0030593 | neutrophil chemotaxis | 0.25 | 0.000504715 | 0.004858883 | 20299/14825 | 2 |
| 127 | Biological Process | GO:0014897 | striated muscle hypertrophy | 0.25 | 0.000535333 | 0.005073115 | 16000/16011 | 2 |
| 128 | Biological Process | GO:0055006 | cardiac cell development | 0.25 | 0.000535333 | 0.005073115 | 16000/22339 | 2 |
| 129 | Biological Process | GO:0014896 | muscle hypertrophy | 0.25 | 0.000556236 | 0.005190114 | 16000/16011 | 2 |
| 130 | Biological Process | GO:2000379 | positive regulation of reactive oxygen species metabolic process | 0.25 | 0.000556236 | 0.005190114 | 13649/14825 | 2 |
| 131 | Biological Process | GO:0048145 | regulation of fibroblast proliferation | 0.25 | 0.000577533 | 0.005347690 | 16000/13649 | 2 |
| 132 | Biological Process | GO:0048144 | fibroblast proliferation | 0.25 | 0.000599222 | 0.005465084 | 16000/13649 | 2 |
| 133 | Biological Process | GO:2000177 | regulation of neural precursor cell proliferation | 0.25 | 0.000599222 | 0.005465084 | 16000/22339 | 2 |
| 134 | Biological Process | GO:0001942 | hair follicle development | 0.25 | 0.000632490 | 0.005725453 | 13649/16011 | 2 |
| 135 | Biological Process | GO:0043500 | muscle adaptation | 0.25 | 0.000643776 | 0.005784442 | 16000/16011 | 2 |
| 136 | Biological Process | GO:0043534 | blood vessel endothelial cell migration | 0.25 | 0.000655159 | 0.005800784 | 16000/22339 | 2 |
| 137 | Biological Process | GO:0098773 | skin epidermis development | 0.25 | 0.000655159 | 0.005800784 | 13649/16011 | 2 |
| 138 | Biological Process | GO:0022404 | molting cycle process | 0.25 | 0.000666639 | 0.005817508 | 13649/16011 | 2 |
| 139 | Biological Process | GO:0022405 | hair cycle process | 0.25 | 0.000666639 | 0.005817508 | 13649/16011 | 2 |
| 140 | Biological Process | GO:0033138 | positive regulation of peptidyl-serine phosphorylation | 0.25 | 0.000689894 | 0.005977439 | 13649/22339 | 2 |
| 141 | Biological Process | GO:1990266 | neutrophil migration | 0.25 | 0.000701668 | 0.006036334 | 20299/14825 | 2 |
| 142 | Biological Process | GO:0014065 | phosphatidylinositol 3-kinase signaling | 0.25 | 0.000725508 | 0.006197473 | 16000/13649 | 2 |
| 143 | Biological Process | GO:0001936 | regulation of endothelial cell proliferation | 0.25 | 0.000812015 | 0.006887934 | 16000/22339 | 2 |
| 144 | Biological Process | GO:0042303 | molting cycle | 0.25 | 0.000876720 | 0.007334215 | 13649/16011 | 2 |
| 145 | Biological Process | GO:0042633 | hair cycle | 0.25 | 0.000876720 | 0.007334215 | 13649/16011 | 2 |
| 146 | Biological Process | GO:0045598 | regulation of fat cell differentiation | 0.25 | 0.000889951 | 0.007343613 | 16000/22339 | 2 |
| 147 | Biological Process | GO:0055123 | digestive system development | 0.25 | 0.000889951 | 0.007343613 | 16000/13649 | 2 |
| 148 | Biological Process | GO:0072089 | stem cell proliferation | 0.25 | 0.000916705 | 0.007513264 | 22339/14825 | 2 |
| 149 | Biological Process | GO:0001935 | endothelial cell proliferation | 0.25 | 0.000999284 | 0.008027361 | 16000/22339 | 2 |
| 150 | Biological Process | GO:0010634 | positive regulation of epithelial cell migration | 0.25 | 0.000999284 | 0.008027361 | 16000/22339 | 2 |
| 151 | Biological Process | GO:0055007 | cardiac muscle cell differentiation | 0.25 | 0.000999284 | 0.008027361 | 16000/22339 | 2 |
| 152 | Biological Process | GO:0048015 | phosphatidylinositol-mediated signaling | 0.25 | 0.001041875 | 0.008314438 | 16000/13649 | 2 |
| 153 | Biological Process | GO:0048017 | inositol lipid-mediated signaling | 0.25 | 0.001085332 | 0.008604628 | 16000/13649 | 2 |
| 154 | Biological Process | GO:0033135 | regulation of peptidyl-serine phosphorylation | 0.25 | 0.001129654 | 0.008897858 | 13649/22339 | 2 |
| 155 | Biological Process | GO:0010594 | regulation of endothelial cell migration | 0.25 | 0.001174839 | 0.009194060 | 16000/22339 | 2 |
| 156 | Biological Process | GO:2001236 | regulation of extrinsic apoptotic signaling pathway | 0.25 | 0.001315558 | 0.010229309 | 16000/22339 | 2 |
| 157 | Biological Process | GO:1901861 | regulation of muscle tissue development | 0.25 | 0.001532438 | 0.011839790 | 16000/16011 | 2 |
| 158 | Biological Process | GO:0010951 | negative regulation of endopeptidase activity | 0.25 | 0.001549785 | 0.011898031 | 16000/22339 | 2 |
| 159 | Biological Process | GO:0061351 | neural precursor cell proliferation | 0.25 | 0.001584762 | 0.012090042 | 16000/22339 | 2 |
| 160 | Biological Process | GO:0055002 | striated muscle cell development | 0.25 | 0.001637937 | 0.012417610 | 16000/22339 | 2 |
| 161 | Biological Process | GO:0048754 | branching morphogenesis of an epithelial tube | 0.25 | 0.001673859 | 0.012611122 | 16000/22339 | 2 |
| 162 | Biological Process | GO:0050731 | positive regulation of peptidyl-tyrosine phosphorylation | 0.25 | 0.001765311 | 0.013218038 | 16000/22339 | 2 |
| 163 | Biological Process | GO:0003018 | vascular process in circulatory system | 0.25 | 0.001840165 | 0.013693987 | 13649/22339 | 2 |
| 164 | Biological Process | GO:0043542 | endothelial cell migration | 0.25 | 0.001878155 | 0.013891472 | 16000/22339 | 2 |
| 165 | Biological Process | GO:0055001 | muscle cell development | 0.25 | 0.001897290 | 0.013947956 | 16000/22339 | 2 |
| 166 | Biological Process | GO:0043281 | regulation of cysteine-type endopeptidase activity involved in apoptotic process | 0.25 | 0.001935842 | 0.014145638 | 16000/22339 | 2 |
| 167 | Biological Process | GO:0048639 | positive regulation of developmental growth | 0.25 | 0.002174994 | 0.015798010 | 16000/22339 | 2 |
| 168 | Biological Process | GO:0006898 | receptor-mediated endocytosis | 0.25 | 0.002257689 | 0.016301054 | 13649/22339 | 2 |
| 169 | Biological Process | GO:0061138 | morphogenesis of a branching epithelium | 0.25 | 0.002405974 | 0.017119716 | 16000/22339 | 2 |
| 170 | Biological Process | GO:2000116 | regulation of cysteine-type endopeptidase activity | 0.25 | 0.002405974 | 0.017119716 | 16000/22339 | 2 |
| 171 | Biological Process | GO:0010001 | glial cell differentiation | 0.25 | 0.002427528 | 0.017119716 | 16000/13649 | 2 |
| 172 | Biological Process | GO:0010632 | regulation of epithelial cell migration | 0.25 | 0.002427528 | 0.017119716 | 16000/22339 | 2 |
| 173 | Biological Process | GO:2001234 | negative regulation of apoptotic signaling pathway | 0.25 | 0.002492742 | 0.017478011 | 16000/22339 | 2 |
| 174 | Biological Process | GO:0001894 | tissue homeostasis | 0.25 | 0.002580984 | 0.017889908 | 13649/22339 | 2 |
| 175 | Biological Process | GO:0097191 | extrinsic apoptotic signaling pathway | 0.25 | 0.002580984 | 0.017889908 | 16000/22339 | 2 |
| 176 | Biological Process | GO:0032872 | regulation of stress-activated MAPK cascade | 0.25 | 0.002625658 | 0.018096151 | 13649/22339 | 2 |
| 177 | Biological Process | GO:0090257 | regulation of muscle system process | 0.25 | 0.002648132 | 0.018147933 | 16000/16011 | 2 |
| 178 | Biological Process | GO:0070302 | regulation of stress-activated protein kinase signaling cascade | 0.25 | 0.002670698 | 0.018199759 | 13649/22339 | 2 |
| 179 | Biological Process | GO:1901215 | negative regulation of neuron death | 0.25 | 0.002693356 | 0.018251628 | 16000/22339 | 2 |
| 180 | Biological Process | GO:0045444 | fat cell differentiation | 0.25 | 0.002738948 | 0.018355490 | 16000/22339 | 2 |
| 181 | Biological Process | GO:0046777 | protein autophosphorylation | 0.25 | 0.002738948 | 0.018355490 | 13649/22339 | 2 |
| 182 | Biological Process | GO:0001763 | morphogenesis of a branching structure | 0.25 | 0.002831230 | 0.018869678 | 16000/22339 | 2 |
| 183 | Biological Process | GO:0010466 | negative regulation of peptidase activity | 0.25 | 0.002877919 | 0.019076044 | 16000/22339 | 2 |
| 184 | Biological Process | GO:0048588 | developmental cell growth | 0.25 | 0.003141232 | 0.020708232 | 16000/22339 | 2 |
| 185 | Biological Process | GO:0048738 | cardiac muscle tissue development | 0.25 | 0.003165716 | 0.020756827 | 16000/22339 | 2 |
| 186 | Biological Process | GO:0051403 | stress-activated MAPK cascade | 0.25 | 0.003214954 | 0.020966342 | 13649/22339 | 2 |
| 187 | Biological Process | GO:0042593 | glucose homeostasis | 0.25 | 0.003239710 | 0.021014801 | 16000/16011 | 2 |
| 188 | Biological Process | GO:0033500 | carbohydrate homeostasis | 0.25 | 0.003264556 | 0.021063329 | 16000/16011 | 2 |
| 189 | Biological Process | GO:0030336 | negative regulation of cell migration | 0.25 | 0.003364844 | 0.021385039 | 16009/16011 | 2 |
| 190 | Biological Process | GO:0048511 | rhythmic process | 0.25 | 0.003595774 | 0.021736493 | 16000/13649 | 2 |
| 191 | Biological Process | GO:2000146 | negative regulation of cell motility | 0.25 | 0.003727217 | 0.021736493 | 16009/16011 | 2 |
| 192 | Biological Process | GO:0071229 | cellular response to acid chemical | 0.25 | 0.003780423 | 0.021736493 | 13649/22339 | 2 |
| 193 | Biological Process | GO:0010631 | epithelial cell migration | 0.25 | 0.003807160 | 0.021783418 | 16000/22339 | 2 |
| 194 | Biological Process | GO:0009895 | negative regulation of catabolic process | 0.25 | 0.003833986 | 0.021833921 | 13649/22339 | 2 |
| 195 | Biological Process | GO:0090132 | epithelium migration | 0.25 | 0.003860902 | 0.021884459 | 16000/22339 | 2 |
| 196 | Biological Process | GO:0007611 | learning or memory | 0.25 | 0.003915002 | 0.021985638 | 16000/13649 | 2 |
| 197 | Biological Process | GO:0090130 | tissue migration | 0.25 | 0.003915002 | 0.021985638 | 16000/22339 | 2 |
| 198 | Biological Process | GO:0050714 | positive regulation of protein secretion | 0.25 | 0.003996822 | 0.022333001 | 16000/13649 | 2 |
| 199 | Biological Process | GO:0043588 | skin development | 0.25 | 0.004024274 | 0.022333001 | 13649/16011 | 2 |
| 200 | Biological Process | GO:0031098 | stress-activated protein kinase signaling cascade | 0.25 | 0.004079445 | 0.022333001 | 13649/22339 | 2 |
| 201 | Biological Process | GO:0046883 | regulation of hormone secretion | 0.25 | 0.004247092 | 0.022620372 | 13649/16009 | 2 |
| 202 | Biological Process | GO:0018105 | peptidyl-serine phosphorylation | 0.25 | 0.004446717 | 0.022620372 | 13649/22339 | 2 |
| 203 | Biological Process | GO:0042063 | gliogenesis | 0.25 | 0.004475589 | 0.022620372 | 16000/13649 | 2 |
| 204 | Biological Process | GO:0021700 | developmental maturation | 0.25 | 0.004650673 | 0.023407745 | 16000/22339 | 2 |
| 205 | Biological Process | GO:0051271 | negative regulation of cellular component movement | 0.25 | 0.004680162 | 0.023458830 | 16009/16011 | 2 |
| 206 | Biological Process | GO:0002793 | positive regulation of peptide secretion | 0.25 | 0.004798999 | 0.023526613 | 16000/13649 | 2 |
| 207 | Biological Process | GO:0050890 | cognition | 0.25 | 0.004858944 | 0.023670277 | 16000/13649 | 2 |
| 208 | Biological Process | GO:0006417 | regulation of translation | 0.25 | 0.004919240 | 0.023765840 | 16000/16011 | 2 |
| 209 | Biological Process | GO:0008544 | epidermis development | 0.25 | 0.005071512 | 0.023765840 | 13649/16011 | 2 |
| 210 | Biological Process | GO:0018209 | peptidyl-serine modification | 0.25 | 0.005071512 | 0.023765840 | 13649/22339 | 2 |
| 211 | Biological Process | GO:0040013 | negative regulation of locomotion | 0.25 | 0.005194903 | 0.023869007 | 16009/16011 | 2 |
| 212 | Biological Process | GO:0052548 | regulation of endopeptidase activity | 0.25 | 0.005288362 | 0.024206730 | 16000/22339 | 2 |
| 213 | Biological Process | GO:0019221 | cytokine-mediated signaling pathway | 0.25 | 0.005319689 | 0.024258584 | 20299/14825 | 2 |
| 214 | Biological Process | GO:0031331 | positive regulation of cellular catabolic process | 0.25 | 0.005509477 | 0.024301803 | 16000/16009 | 2 |
| 215 | Biological Process | GO:0031349 | positive regulation of defense response | 0.25 | 0.005509477 | 0.024301803 | 13649/14825 | 2 |
| 216 | Biological Process | GO:0043010 | camera-type eye development | 0.25 | 0.005573434 | 0.024494838 | 13649/22339 | 2 |
| 217 | Biological Process | GO:0001655 | urogenital system development | 0.25 | 0.005637737 | 0.024674814 | 16000/22339 | 2 |
| 218 | Biological Process | GO:0045861 | negative regulation of proteolysis | 0.25 | 0.005767381 | 0.024674814 | 16000/22339 | 2 |
| 219 | Biological Process | GO:0042060 | wound healing | 0.25 | 0.005800008 | 0.024674814 | 16000/13649 | 2 |
| 220 | Biological Process | GO:0046879 | hormone secretion | 0.25 | 0.006064125 | 0.025158092 | 13649/16009 | 2 |
| 221 | Biological Process | GO:1901214 | regulation of neuron death | 0.25 | 0.006064125 | 0.025158092 | 16000/22339 | 2 |
| 222 | Biological Process | GO:0001101 | response to acid chemical | 0.25 | 0.006299737 | 0.025557127 | 13649/22339 | 2 |
| 223 | Biological Process | GO:0034248 | regulation of cellular amide metabolic process | 0.25 | 0.006333738 | 0.025606557 | 16000/16011 | 2 |
| 224 | Biological Process | GO:0009914 | hormone transport | 0.25 | 0.006367825 | 0.025606557 | 13649/16009 | 2 |
| 225 | Biological Process | GO:0060562 | epithelial tube morphogenesis | 0.25 | 0.006818716 | 0.026023799 | 16000/22339 | 2 |
| 226 | Biological Process | GO:0003012 | muscle system process | 0.25 | 0.006995959 | 0.026190057 | 16000/16011 | 2 |
| 227 | Biological Process | GO:0022407 | regulation of cell-cell adhesion | 0.25 | 0.007067449 | 0.026190057 | 16000/22339 | 2 |
| 228 | Biological Process | GO:0060249 | anatomical structure homeostasis | 0.25 | 0.007211442 | 0.026190057 | 13649/22339 | 2 |
| 229 | Biological Process | GO:0001654 | eye development | 0.25 | 0.007283945 | 0.026295907 | 13649/22339 | 2 |
| 230 | Biological Process | GO:0150063 | visual system development | 0.25 | 0.007283945 | 0.026295907 | 13649/22339 | 2 |
| 231 | Biological Process | GO:0048880 | sensory system development | 0.25 | 0.007393330 | 0.026467108 | 13649/22339 | 2 |
| 232 | Biological Process | GO:0043900 | regulation of multi-organism process | 0.25 | 0.007429960 | 0.026467108 | 16000/14825 | 2 |
| 233 | Biological Process | GO:0048638 | regulation of developmental growth | 0.25 | 0.007429960 | 0.026467108 | 16000/22339 | 2 |
| 234 | Biological Process | GO:0070997 | neuron death | 0.25 | 0.007429960 | 0.026467108 | 16000/22339 | 2 |
| 235 | Biological Process | GO:0051346 | negative regulation of hydrolase activity | 0.25 | 0.007466675 | 0.026467108 | 16000/22339 | 2 |
| 236 | Biological Process | GO:1903706 | regulation of hemopoiesis | 0.25 | 0.007577321 | 0.026487868 | 22339/14825 | 2 |
| 237 | Biological Process | GO:0045785 | positive regulation of cell adhesion | 0.25 | 0.007688722 | 0.026738641 | 16000/22339 | 2 |
| 238 | Biological Process | GO:0009896 | positive regulation of catabolic process | 0.25 | 0.007763408 | 0.026738641 | 16000/16009 | 2 |
| 239 | Biological Process | GO:0001933 | negative regulation of protein phosphorylation | 0.25 | 0.007800876 | 0.026738641 | 16000/16009 | 2 |
| 240 | Biological Process | GO:0001667 | ameboidal-type cell migration | 0.25 | 0.007838428 | 0.026738641 | 16000/22339 | 2 |
| 241 | Biological Process | GO:0052547 | regulation of peptidase activity | 0.25 | 0.007838428 | 0.026738641 | 16000/22339 | 2 |
| 242 | Biological Process | GO:2001233 | regulation of apoptotic signaling pathway | 0.25 | 0.007876064 | 0.026738641 | 16000/22339 | 2 |
| 243 | Biological Process | GO:0010959 | regulation of metal ion transport | 0.25 | 0.007989471 | 0.026995064 | 16000/14825 | 2 |
| 244 | Biological Process | GO:0051222 | positive regulation of protein transport | 0.25 | 0.008606945 | 0.027766554 | 16000/13649 | 2 |
| 245 | Biological Process | GO:0014706 | striated muscle tissue development | 0.25 | 0.009245561 | 0.028195226 | 16000/22339 | 2 |
| 246 | Biological Process | GO:1904951 | positive regulation of establishment of protein localization | 0.25 | 0.009326866 | 0.028195226 | 16000/13649 | 2 |
| 247 | Biological Process | GO:0042326 | negative regulation of phosphorylation | 0.25 | 0.009490454 | 0.028195226 | 16000/16009 | 2 |
| 248 | Biological Process | GO:1903532 | positive regulation of secretion by cell | 0.25 | 0.009821544 | 0.028711173 | 16000/13649 | 2 |
| 249 | Biological Process | GO:0050708 | regulation of protein secretion | 0.25 | 0.010115512 | 0.029145167 | 16000/13649 | 2 |
| 250 | Biological Process | GO:0008015 | blood circulation | 0.25 | 0.010370638 | 0.029529540 | 13649/22339 | 2 |
| 251 | Biological Process | GO:0048871 | multicellular organismal homeostasis | 0.25 | 0.010671952 | 0.030175007 | 13649/22339 | 2 |
| 252 | Biological Process | GO:0003013 | circulatory system process | 0.25 | 0.010758765 | 0.030296437 | 13649/22339 | 2 |
| 253 | Biological Process | GO:0009611 | response to wounding | 0.25 | 0.010933353 | 0.030296437 | 16000/13649 | 2 |
| 254 | Biological Process | GO:0048568 | embryonic organ development | 0.25 | 0.011330851 | 0.030955679 | 13649/22339 | 2 |
| 255 | Biological Process | GO:0002791 | regulation of peptide secretion | 0.25 | 0.011780074 | 0.031824566 | 16000/13649 | 2 |
| 256 | Biological Process | GO:0007346 | regulation of mitotic cell cycle | 0.25 | 0.011825433 | 0.031876111 | 16000/13649 | 2 |
| 257 | Biological Process | GO:0021940 | positive regulation of cerebellar granule cell precursor proliferation | 0.125 | 0.003437826 | 0.021385039 | 16000 | 1 |
| 258 | Biological Process | GO:0060215 | primitive hemopoiesis | 0.125 | 0.003437826 | 0.021385039 | 22339 | 1 |
| 259 | Biological Process | GO:0060982 | coronary artery morphogenesis | 0.125 | 0.003437826 | 0.021385039 | 22339 | 1 |
| 260 | Biological Process | GO:0150065 | regulation of deacetylase activity | 0.125 | 0.003437826 | 0.021385039 | 22339 | 1 |
| 261 | Biological Process | GO:1905288 | vascular associated smooth muscle cell apoptotic process | 0.125 | 0.003437826 | 0.021385039 | 16000 | 1 |
| 262 | Biological Process | GO:1905459 | regulation of vascular associated smooth muscle cell apoptotic process | 0.125 | 0.003437826 | 0.021385039 | 16000 | 1 |
| 263 | Biological Process | GO:0031077 | post-embryonic camera-type eye development | 0.125 | 0.003781039 | 0.021736493 | 22339 | 1 |
| 264 | Biological Process | GO:0034392 | negative regulation of smooth muscle cell apoptotic process | 0.125 | 0.003781039 | 0.021736493 | 16000 | 1 |
| 265 | Biological Process | GO:0035630 | bone mineralization involved in bone maturation | 0.125 | 0.003781039 | 0.021736493 | 16000 | 1 |
| 266 | Biological Process | GO:0048845 | venous blood vessel morphogenesis | 0.125 | 0.003781039 | 0.021736493 | 22339 | 1 |
| 267 | Biological Process | GO:0060056 | mammary gland involution | 0.125 | 0.003781039 | 0.021736493 | 16011 | 1 |
| 268 | Biological Process | GO:0060346 | bone trabecula formation | 0.125 | 0.003781039 | 0.021736493 | 22339 | 1 |
| 269 | Biological Process | GO:0070943 | neutrophil mediated killing of symbiont cell | 0.125 | 0.003781039 | 0.021736493 | 14825 | 1 |
| 270 | Biological Process | GO:0097084 | vascular smooth muscle cell development | 0.125 | 0.003781039 | 0.021736493 | 22339 | 1 |
| 271 | Biological Process | GO:1902510 | regulation of apoptotic DNA fragmentation | 0.125 | 0.003781039 | 0.021736493 | 16009 | 1 |
| 272 | Biological Process | GO:1902946 | protein localization to early endosome | 0.125 | 0.003781039 | 0.021736493 | 22339 | 1 |
| 273 | Biological Process | GO:1905666 | regulation of protein localization to endosome | 0.125 | 0.003781039 | 0.021736493 | 22339 | 1 |
| 274 | Biological Process | GO:1905668 | positive regulation of protein localization to endosome | 0.125 | 0.003781039 | 0.021736493 | 22339 | 1 |
| 275 | Biological Process | GO:2000288 | positive regulation of myoblast proliferation | 0.125 | 0.003781039 | 0.021736493 | 16000 | 1 |
| 276 | Biological Process | GO:0014745 | negative regulation of muscle adaptation | 0.125 | 0.004124149 | 0.022333001 | 16011 | 1 |
| 277 | Biological Process | GO:0034350 | regulation of glial cell apoptotic process | 0.125 | 0.004124149 | 0.022333001 | 16000 | 1 |
| 278 | Biological Process | GO:0048143 | astrocyte activation | 0.125 | 0.004124149 | 0.022333001 | 13649 | 1 |
| 279 | Biological Process | GO:0060525 | prostate glandular acinus development | 0.125 | 0.004124149 | 0.022333001 | 16000 | 1 |
| 280 | Biological Process | GO:0061299 | retina vasculature morphogenesis in camera-type eye | 0.125 | 0.004124149 | 0.022333001 | 22339 | 1 |
| 281 | Biological Process | GO:0002551 | mast cell chemotaxis | 0.125 | 0.004467155 | 0.022620372 | 22339 | 1 |
| 282 | Biological Process | GO:0021924 | cell proliferation in external granule layer | 0.125 | 0.004467155 | 0.022620372 | 16000 | 1 |
| 283 | Biological Process | GO:0021930 | cerebellar granule cell precursor proliferation | 0.125 | 0.004467155 | 0.022620372 | 16000 | 1 |
| 284 | Biological Process | GO:0021936 | regulation of cerebellar granule cell precursor proliferation | 0.125 | 0.004467155 | 0.022620372 | 16000 | 1 |
| 285 | Biological Process | GO:0043117 | positive regulation of vascular permeability | 0.125 | 0.004467155 | 0.022620372 | 22339 | 1 |
| 286 | Biological Process | GO:0060281 | regulation of oocyte development | 0.125 | 0.004467155 | 0.022620372 | 16000 | 1 |
| 287 | Biological Process | GO:0061140 | lung secretory cell differentiation | 0.125 | 0.004467155 | 0.022620372 | 16000 | 1 |
| 288 | Biological Process | GO:0061418 | regulation of transcription from RNA polymerase II promoter in response to hypoxia | 0.125 | 0.004467155 | 0.022620372 | 22339 | 1 |
| 289 | Biological Process | GO:0070942 | neutrophil mediated cytotoxicity | 0.125 | 0.004467155 | 0.022620372 | 14825 | 1 |
| 290 | Biological Process | GO:0071679 | commissural neuron axon guidance | 0.125 | 0.004467155 | 0.022620372 | 22339 | 1 |
| 291 | Biological Process | GO:1903587 | regulation of blood vessel endothelial cell proliferation involved in sprouting angiogenesis | 0.125 | 0.004467155 | 0.022620372 | 22339 | 1 |
| 292 | Biological Process | GO:1903624 | regulation of DNA catabolic process | 0.125 | 0.004467155 | 0.022620372 | 16009 | 1 |
| 293 | Biological Process | GO:1905879 | regulation of oogenesis | 0.125 | 0.004467155 | 0.022620372 | 16000 | 1 |
| 294 | Biological Process | GO:0021534 | cell proliferation in hindbrain | 0.125 | 0.004810058 | 0.023526613 | 16000 | 1 |
| 295 | Biological Process | GO:0030949 | positive regulation of vascular endothelial growth factor receptor signaling pathway | 0.125 | 0.004810058 | 0.023526613 | 22339 | 1 |
| 296 | Biological Process | GO:0051712 | positive regulation of killing of cells of other organism | 0.125 | 0.004810058 | 0.023526613 | 14825 | 1 |
| 297 | Biological Process | GO:0060736 | prostate gland growth | 0.125 | 0.004810058 | 0.023526613 | 16000 | 1 |
| 298 | Biological Process | GO:0097531 | mast cell migration | 0.125 | 0.004810058 | 0.023526613 | 22339 | 1 |
| 299 | Biological Process | GO:0014733 | regulation of skeletal muscle adaptation | 0.125 | 0.005152857 | 0.023765840 | 16011 | 1 |
| 300 | Biological Process | GO:0043129 | surfactant homeostasis | 0.125 | 0.005152857 | 0.023765840 | 22339 | 1 |
| 301 | Biological Process | GO:0045779 | negative regulation of bone resorption | 0.125 | 0.005152857 | 0.023765840 | 22339 | 1 |
| 302 | Biological Process | GO:0050930 | induction of positive chemotaxis | 0.125 | 0.005152857 | 0.023765840 | 22339 | 1 |
| 303 | Biological Process | GO:0051709 | regulation of killing of cells of other organism | 0.125 | 0.005152857 | 0.023765840 | 14825 | 1 |
| 304 | Biological Process | GO:0060442 | branching involved in prostate gland morphogenesis | 0.125 | 0.005152857 | 0.023765840 | 16000 | 1 |
| 305 | Biological Process | GO:0061029 | eyelid development in camera-type eye | 0.125 | 0.005152857 | 0.023765840 | 13649 | 1 |
| 306 | Biological Process | GO:0061051 | positive regulation of cell growth involved in cardiac muscle cell development | 0.125 | 0.005152857 | 0.023765840 | 16000 | 1 |
| 307 | Biological Process | GO:0061430 | bone trabecula morphogenesis | 0.125 | 0.005152857 | 0.023765840 | 22339 | 1 |
| 308 | Biological Process | GO:1900119 | positive regulation of execution phase of apoptosis | 0.125 | 0.005152857 | 0.023765840 | 16009 | 1 |
| 309 | Biological Process | GO:1905564 | positive regulation of vascular endothelial cell proliferation | 0.125 | 0.005152857 | 0.023765840 | 16000 | 1 |
| 310 | Biological Process | GO:0032793 | positive regulation of CREB transcription factor activity | 0.125 | 0.005495553 | 0.024301803 | 22339 | 1 |
| 311 | Biological Process | GO:0034349 | glial cell apoptotic process | 0.125 | 0.005495553 | 0.024301803 | 16000 | 1 |
| 312 | Biological Process | GO:0045821 | positive regulation of glycolytic process | 0.125 | 0.005495553 | 0.024301803 | 16000 | 1 |
| 313 | Biological Process | GO:0051852 | disruption by host of symbiont cells | 0.125 | 0.005495553 | 0.024301803 | 14825 | 1 |
| 314 | Biological Process | GO:0051873 | killing by host of symbiont cells | 0.125 | 0.005495553 | 0.024301803 | 14825 | 1 |
| 315 | Biological Process | GO:0060766 | negative regulation of androgen receptor signaling pathway | 0.125 | 0.005495553 | 0.024301803 | 16000 | 1 |
| 316 | Biological Process | GO:2000291 | regulation of myoblast proliferation | 0.125 | 0.005495553 | 0.024301803 | 16000 | 1 |
| 317 | Biological Process | GO:0010960 | magnesium ion homeostasis | 0.125 | 0.005838146 | 0.024674814 | 13649 | 1 |
| 318 | Biological Process | GO:0030813 | positive regulation of nucleotide catabolic process | 0.125 | 0.005838146 | 0.024674814 | 16000 | 1 |
| 319 | Biological Process | GO:0045725 | positive regulation of glycogen biosynthetic process | 0.125 | 0.005838146 | 0.024674814 | 16000 | 1 |
| 320 | Biological Process | GO:0048739 | cardiac muscle fiber development | 0.125 | 0.005838146 | 0.024674814 | 22339 | 1 |
| 321 | Biological Process | GO:0048875 | chemical homeostasis within a tissue | 0.125 | 0.005838146 | 0.024674814 | 22339 | 1 |
| 322 | Biological Process | GO:0051197 | positive regulation of coenzyme metabolic process | 0.125 | 0.005838146 | 0.024674814 | 16000 | 1 |
| 323 | Biological Process | GO:0090036 | regulation of protein kinase C signaling | 0.125 | 0.005838146 | 0.024674814 | 22339 | 1 |
| 324 | Biological Process | GO:2001028 | positive regulation of endothelial cell chemotaxis | 0.125 | 0.005838146 | 0.024674814 | 22339 | 1 |
| 325 | Biological Process | GO:0033160 | positive regulation of protein import into nucleus, translocation | 0.125 | 0.006180636 | 0.025158092 | 16000 | 1 |
| 326 | Biological Process | GO:0046851 | negative regulation of bone remodeling | 0.125 | 0.006180636 | 0.025158092 | 22339 | 1 |
| 327 | Biological Process | GO:0050650 | chondroitin sulfate proteoglycan biosynthetic process | 0.125 | 0.006180636 | 0.025158092 | 16000 | 1 |
| 328 | Biological Process | GO:0060841 | venous blood vessel development | 0.125 | 0.006180636 | 0.025158092 | 22339 | 1 |
| 329 | Biological Process | GO:0060947 | cardiac vascular smooth muscle cell differentiation | 0.125 | 0.006180636 | 0.025158092 | 22339 | 1 |
| 330 | Biological Process | GO:0070886 | positive regulation of calcineurin-NFAT signaling cascade | 0.125 | 0.006180636 | 0.025158092 | 16000 | 1 |
| 331 | Biological Process | GO:0090030 | regulation of steroid hormone biosynthetic process | 0.125 | 0.006180636 | 0.025158092 | 16000 | 1 |
| 332 | Biological Process | GO:0106058 | positive regulation of calcineurin-mediated signaling | 0.125 | 0.006180636 | 0.025158092 | 16000 | 1 |
| 333 | Biological Process | GO:1904754 | positive regulation of vascular associated smooth muscle cell migration | 0.125 | 0.006180636 | 0.025158092 | 16011 | 1 |
| 334 | Biological Process | GO:0002043 | blood vessel endothelial cell proliferation involved in sprouting angiogenesis | 0.125 | 0.006523022 | 0.025606557 | 22339 | 1 |
| 335 | Biological Process | GO:0006309 | apoptotic DNA fragmentation | 0.125 | 0.006523022 | 0.025606557 | 16009 | 1 |
| 336 | Biological Process | GO:0010666 | positive regulation of cardiac muscle cell apoptotic process | 0.125 | 0.006523022 | 0.025606557 | 16009 | 1 |
| 337 | Biological Process | GO:0043931 | ossification involved in bone maturation | 0.125 | 0.006523022 | 0.025606557 | 16000 | 1 |
| 338 | Biological Process | GO:0046886 | positive regulation of hormone biosynthetic process | 0.125 | 0.006523022 | 0.025606557 | 16000 | 1 |
| 339 | Biological Process | GO:0070875 | positive regulation of glycogen metabolic process | 0.125 | 0.006523022 | 0.025606557 | 16000 | 1 |
| 340 | Biological Process | GO:0090201 | negative regulation of release of cytochrome c from mitochondria | 0.125 | 0.006523022 | 0.025606557 | 16000 | 1 |
| 341 | Biological Process | GO:1903798 | regulation of production of miRNAs involved in gene silencing by miRNA | 0.125 | 0.006523022 | 0.025606557 | 13649 | 1 |
| 342 | Biological Process | GO:0001946 | lymphangiogenesis | 0.125 | 0.006865306 | 0.026023799 | 22339 | 1 |
| 343 | Biological Process | GO:0010560 | positive regulation of glycoprotein biosynthetic process | 0.125 | 0.006865306 | 0.026023799 | 16000 | 1 |
| 344 | Biological Process | GO:0010663 | positive regulation of striated muscle cell apoptotic process | 0.125 | 0.006865306 | 0.026023799 | 16009 | 1 |
| 345 | Biological Process | GO:0032148 | activation of protein kinase B activity | 0.125 | 0.006865306 | 0.026023799 | 16000 | 1 |
| 346 | Biological Process | GO:0048245 | eosinophil chemotaxis | 0.125 | 0.006865306 | 0.026023799 | 20299 | 1 |
| 347 | Biological Process | GO:0051818 | disruption of cells of other organism involved in symbiotic interaction | 0.125 | 0.006865306 | 0.026023799 | 14825 | 1 |
| 348 | Biological Process | GO:0051883 | killing of cells in other organism involved in symbiotic interaction | 0.125 | 0.006865306 | 0.026023799 | 14825 | 1 |
| 349 | Biological Process | GO:0060252 | positive regulation of glial cell proliferation | 0.125 | 0.006865306 | 0.026023799 | 16000 | 1 |
| 350 | Biological Process | GO:0070920 | regulation of production of small RNA involved in gene silencing by RNA | 0.125 | 0.006865306 | 0.026023799 | 13649 | 1 |
| 351 | Biological Process | GO:0070977 | bone maturation | 0.125 | 0.006865306 | 0.026023799 | 16000 | 1 |
| 352 | Biological Process | GO:0031065 | positive regulation of histone deacetylation | 0.125 | 0.007207486 | 0.026190057 | 22339 | 1 |
| 353 | Biological Process | GO:0031290 | retinal ganglion cell axon guidance | 0.125 | 0.007207486 | 0.026190057 | 22339 | 1 |
| 354 | Biological Process | GO:0036010 | protein localization to endosome | 0.125 | 0.007207486 | 0.026190057 | 22339 | 1 |
| 355 | Biological Process | GO:0036303 | lymph vessel morphogenesis | 0.125 | 0.007207486 | 0.026190057 | 22339 | 1 |
| 356 | Biological Process | GO:0043501 | skeletal muscle adaptation | 0.125 | 0.007207486 | 0.026190057 | 16011 | 1 |
| 357 | Biological Process | GO:0048569 | post-embryonic animal organ development | 0.125 | 0.007207486 | 0.026190057 | 22339 | 1 |
| 358 | Biological Process | GO:0051450 | myoblast proliferation | 0.125 | 0.007207486 | 0.026190057 | 16000 | 1 |
| 359 | Biological Process | GO:0090050 | positive regulation of cell migration involved in sprouting angiogenesis | 0.125 | 0.007207486 | 0.026190057 | 22339 | 1 |
| 360 | Biological Process | GO:0101023 | vascular endothelial cell proliferation | 0.125 | 0.007207486 | 0.026190057 | 16000 | 1 |
| 361 | Biological Process | GO:1901522 | positive regulation of transcription from RNA polymerase II promoter involved in cellular response to chemical stimulus | 0.125 | 0.007207486 | 0.026190057 | 22339 | 1 |
| 362 | Biological Process | GO:1905562 | regulation of vascular endothelial cell proliferation | 0.125 | 0.007207486 | 0.026190057 | 16000 | 1 |
| 363 | Biological Process | GO:0051194 | positive regulation of cofactor metabolic process | 0.125 | 0.007549562 | 0.026467108 | 16000 | 1 |
| 364 | Biological Process | GO:0060749 | mammary gland alveolus development | 0.125 | 0.007549562 | 0.026467108 | 22339 | 1 |
| 365 | Biological Process | GO:0061377 | mammary gland lobule development | 0.125 | 0.007549562 | 0.026467108 | 22339 | 1 |
| 366 | Biological Process | GO:1903020 | positive regulation of glycoprotein metabolic process | 0.125 | 0.007549562 | 0.026467108 | 16000 | 1 |
| 367 | Biological Process | GO:2001026 | regulation of endothelial cell chemotaxis | 0.125 | 0.007549562 | 0.026467108 | 22339 | 1 |
| 368 | Biological Process | GO:0031954 | positive regulation of protein autophosphorylation | 0.125 | 0.007891536 | 0.026738641 | 22339 | 1 |
| 369 | Biological Process | GO:0071276 | cellular response to cadmium ion | 0.125 | 0.007891536 | 0.026738641 | 13649 | 1 |
| 370 | Biological Process | GO:0090190 | positive regulation of branching involved in ureteric bud morphogenesis | 0.125 | 0.007891536 | 0.026738641 | 22339 | 1 |
| 371 | Biological Process | GO:1905208 | negative regulation of cardiocyte differentiation | 0.125 | 0.007891536 | 0.026738641 | 13649 | 1 |
| 372 | Biological Process | GO:2000637 | positive regulation of gene silencing by miRNA | 0.125 | 0.007891536 | 0.026738641 | 13649 | 1 |
| 373 | Biological Process | GO:0032352 | positive regulation of hormone metabolic process | 0.125 | 0.008233407 | 0.027138920 | 16000 | 1 |
| 374 | Biological Process | GO:0034104 | negative regulation of tissue remodeling | 0.125 | 0.008233407 | 0.027138920 | 22339 | 1 |
| 375 | Biological Process | GO:0048799 | animal organ maturation | 0.125 | 0.008233407 | 0.027138920 | 16000 | 1 |
| 376 | Biological Process | GO:0060148 | positive regulation of posttranscriptional gene silencing | 0.125 | 0.008233407 | 0.027138920 | 13649 | 1 |
| 377 | Biological Process | GO:0071392 | cellular response to estradiol stimulus | 0.125 | 0.008233407 | 0.027138920 | 13649 | 1 |
| 378 | Biological Process | GO:0072677 | eosinophil migration | 0.125 | 0.008233407 | 0.027138920 | 20299 | 1 |
| 379 | Biological Process | GO:1904738 | vascular associated smooth muscle cell migration | 0.125 | 0.008233407 | 0.027138920 | 16011 | 1 |
| 380 | Biological Process | GO:1904752 | regulation of vascular associated smooth muscle cell migration | 0.125 | 0.008233407 | 0.027138920 | 16011 | 1 |
| 381 | Biological Process | GO:2000679 | positive regulation of transcription regulatory region DNA binding | 0.125 | 0.008233407 | 0.027138920 | 16000 | 1 |
| 382 | Biological Process | GO:0032878 | regulation of establishment or maintenance of cell polarity | 0.125 | 0.008575174 | 0.027737830 | 16000 | 1 |
| 383 | Biological Process | GO:0050832 | defense response to fungus | 0.125 | 0.008575174 | 0.027737830 | 14825 | 1 |
| 384 | Biological Process | GO:0051894 | positive regulation of focal adhesion assembly | 0.125 | 0.008575174 | 0.027737830 | 22339 | 1 |
| 385 | Biological Process | GO:0061213 | positive regulation of mesonephros development | 0.125 | 0.008575174 | 0.027737830 | 22339 | 1 |
| 386 | Biological Process | GO:0071425 | hematopoietic stem cell proliferation | 0.125 | 0.008575174 | 0.027737830 | 14825 | 1 |
| 387 | Biological Process | GO:1901532 | regulation of hematopoietic progenitor cell differentiation | 0.125 | 0.008575174 | 0.027737830 | 22339 | 1 |
| 388 | Biological Process | GO:1904646 | cellular response to amyloid-beta | 0.125 | 0.008575174 | 0.027737830 | 16000 | 1 |
| 389 | Biological Process | GO:0000737 | DNA catabolic process, endonucleolytic | 0.125 | 0.008916839 | 0.028195226 | 16009 | 1 |
| 390 | Biological Process | GO:0033158 | regulation of protein import into nucleus, translocation | 0.125 | 0.008916839 | 0.028195226 | 16000 | 1 |
| 391 | Biological Process | GO:0042104 | positive regulation of activated T cell proliferation | 0.125 | 0.008916839 | 0.028195226 | 16000 | 1 |
| 392 | Biological Process | GO:0050927 | positive regulation of positive chemotaxis | 0.125 | 0.008916839 | 0.028195226 | 22339 | 1 |
| 393 | Biological Process | GO:0090189 | regulation of branching involved in ureteric bud morphogenesis | 0.125 | 0.008916839 | 0.028195226 | 22339 | 1 |
| 394 | Biological Process | GO:2001171 | positive regulation of ATP biosynthetic process | 0.125 | 0.008916839 | 0.028195226 | 16000 | 1 |
| 395 | Biological Process | GO:0002052 | positive regulation of neuroblast proliferation | 0.125 | 0.009258400 | 0.028195226 | 22339 | 1 |
| 396 | Biological Process | GO:0010893 | positive regulation of steroid biosynthetic process | 0.125 | 0.009258400 | 0.028195226 | 16000 | 1 |
| 397 | Biological Process | GO:0031063 | regulation of histone deacetylation | 0.125 | 0.009258400 | 0.028195226 | 22339 | 1 |
| 398 | Biological Process | GO:0032369 | negative regulation of lipid transport | 0.125 | 0.009258400 | 0.028195226 | 16009 | 1 |
| 399 | Biological Process | GO:0036003 | positive regulation of transcription from RNA polymerase II promoter in response to stress | 0.125 | 0.009258400 | 0.028195226 | 22339 | 1 |
| 400 | Biological Process | GO:0045780 | positive regulation of bone resorption | 0.125 | 0.009258400 | 0.028195226 | 13649 | 1 |
| 401 | Biological Process | GO:0046852 | positive regulation of bone remodeling | 0.125 | 0.009258400 | 0.028195226 | 13649 | 1 |
| 402 | Biological Process | GO:0050926 | regulation of positive chemotaxis | 0.125 | 0.009258400 | 0.028195226 | 22339 | 1 |
| 403 | Biological Process | GO:0060343 | trabecula formation | 0.125 | 0.009258400 | 0.028195226 | 22339 | 1 |
| 404 | Biological Process | GO:0061050 | regulation of cell growth involved in cardiac muscle cell development | 0.125 | 0.009258400 | 0.028195226 | 16000 | 1 |
| 405 | Biological Process | GO:1900745 | positive regulation of p38MAPK cascade | 0.125 | 0.009258400 | 0.028195226 | 22339 | 1 |
| 406 | Biological Process | GO:1904645 | response to amyloid-beta | 0.125 | 0.009258400 | 0.028195226 | 16000 | 1 |
| 407 | Biological Process | GO:0001832 | blastocyst growth | 0.125 | 0.009599859 | 0.028195226 | 16000 | 1 |
| 408 | Biological Process | GO:0001945 | lymph vessel development | 0.125 | 0.009599859 | 0.028195226 | 22339 | 1 |
| 409 | Biological Process | GO:0005979 | regulation of glycogen biosynthetic process | 0.125 | 0.009599859 | 0.028195226 | 16000 | 1 |
| 410 | Biological Process | GO:0010719 | negative regulation of epithelial to mesenchymal transition | 0.125 | 0.009599859 | 0.028195226 | 22339 | 1 |
| 411 | Biological Process | GO:0010962 | regulation of glucan biosynthetic process | 0.125 | 0.009599859 | 0.028195226 | 16000 | 1 |
| 412 | Biological Process | GO:0030224 | monocyte differentiation | 0.125 | 0.009599859 | 0.028195226 | 22339 | 1 |
| 413 | Biological Process | GO:0034390 | smooth muscle cell apoptotic process | 0.125 | 0.009599859 | 0.028195226 | 16000 | 1 |
| 414 | Biological Process | GO:0034391 | regulation of smooth muscle cell apoptotic process | 0.125 | 0.009599859 | 0.028195226 | 16000 | 1 |
| 415 | Biological Process | GO:0035162 | embryonic hemopoiesis | 0.125 | 0.009599859 | 0.028195226 | 22339 | 1 |
| 416 | Biological Process | GO:0045663 | positive regulation of myoblast differentiation | 0.125 | 0.009599859 | 0.028195226 | 16009 | 1 |
| 417 | Biological Process | GO:0060765 | regulation of androgen receptor signaling pathway | 0.125 | 0.009599859 | 0.028195226 | 16000 | 1 |
| 418 | Biological Process | GO:0061217 | regulation of mesonephros development | 0.125 | 0.009599859 | 0.028195226 | 22339 | 1 |
| 419 | Biological Process | GO:0061298 | retina vasculature development in camera-type eye | 0.125 | 0.009599859 | 0.028195226 | 22339 | 1 |
| 420 | Biological Process | GO:0090312 | positive regulation of protein deacetylation | 0.125 | 0.009599859 | 0.028195226 | 22339 | 1 |
| 421 | Biological Process | GO:1903131 | mononuclear cell differentiation | 0.125 | 0.009599859 | 0.028195226 | 22339 | 1 |
| 422 | Biological Process | GO:2000727 | positive regulation of cardiac muscle cell differentiation | 0.125 | 0.009599859 | 0.028195226 | 16000 | 1 |
| 423 | Biological Process | GO:0002092 | positive regulation of receptor internalization | 0.125 | 0.009941214 | 0.028711173 | 22339 | 1 |
| 424 | Biological Process | GO:0030262 | apoptotic nuclear changes | 0.125 | 0.009941214 | 0.028711173 | 16009 | 1 |
| 425 | Biological Process | GO:0043114 | regulation of vascular permeability | 0.125 | 0.009941214 | 0.028711173 | 22339 | 1 |
| 426 | Biological Process | GO:0050654 | chondroitin sulfate proteoglycan metabolic process | 0.125 | 0.009941214 | 0.028711173 | 16000 | 1 |
| 427 | Biological Process | GO:0060571 | morphogenesis of an epithelial fold | 0.125 | 0.009941214 | 0.028711173 | 13649 | 1 |
| 428 | Biological Process | GO:1900117 | regulation of execution phase of apoptosis | 0.125 | 0.009941214 | 0.028711173 | 16009 | 1 |
| 429 | Biological Process | GO:0006921 | cellular component disassembly involved in execution phase of apoptosis | 0.125 | 0.010282467 | 0.029347369 | 16009 | 1 |
| 430 | Biological Process | GO:0014002 | astrocyte development | 0.125 | 0.010282467 | 0.029347369 | 13649 | 1 |
| 431 | Biological Process | GO:0030947 | regulation of vascular endothelial growth factor receptor signaling pathway | 0.125 | 0.010282467 | 0.029347369 | 22339 | 1 |
| 432 | Biological Process | GO:0035767 | endothelial cell chemotaxis | 0.125 | 0.010282467 | 0.029347369 | 22339 | 1 |
| 433 | Biological Process | GO:0050999 | regulation of nitric-oxide synthase activity | 0.125 | 0.010623616 | 0.030108519 | 13649 | 1 |
| 434 | Biological Process | GO:1903393 | positive regulation of adherens junction organization | 0.125 | 0.010623616 | 0.030108519 | 22339 | 1 |
| 435 | Biological Process | GO:0006308 | DNA catabolic process | 0.125 | 0.010964663 | 0.030296437 | 16009 | 1 |
| 436 | Biological Process | GO:0014741 | negative regulation of muscle hypertrophy | 0.125 | 0.010964663 | 0.030296437 | 16011 | 1 |
| 437 | Biological Process | GO:0030810 | positive regulation of nucleotide biosynthetic process | 0.125 | 0.010964663 | 0.030296437 | 16000 | 1 |
| 438 | Biological Process | GO:0060487 | lung epithelial cell differentiation | 0.125 | 0.010964663 | 0.030296437 | 16000 | 1 |
| 439 | Biological Process | GO:0060977 | coronary vasculature morphogenesis | 0.125 | 0.010964663 | 0.030296437 | 22339 | 1 |
| 440 | Biological Process | GO:0070528 | protein kinase C signaling | 0.125 | 0.010964663 | 0.030296437 | 22339 | 1 |
| 441 | Biological Process | GO:1900373 | positive regulation of purine nucleotide biosynthetic process | 0.125 | 0.010964663 | 0.030296437 | 16000 | 1 |
| 442 | Biological Process | GO:1901890 | positive regulation of cell junction assembly | 0.125 | 0.010964663 | 0.030296437 | 22339 | 1 |
| 443 | Biological Process | GO:0002446 | neutrophil mediated immunity | 0.125 | 0.011305607 | 0.030955679 | 14825 | 1 |
| 444 | Biological Process | GO:0048841 | regulation of axon extension involved in axon guidance | 0.125 | 0.011305607 | 0.030955679 | 22339 | 1 |
| 445 | Biological Process | GO:0060479 | lung cell differentiation | 0.125 | 0.011305607 | 0.030955679 | 16000 | 1 |
| 446 | Biological Process | GO:0060740 | prostate gland epithelium morphogenesis | 0.125 | 0.011305607 | 0.030955679 | 16000 | 1 |
| 447 | Biological Process | GO:0031069 | hair follicle morphogenesis | 0.125 | 0.011646448 | 0.031533796 | 16011 | 1 |
| 448 | Biological Process | GO:0038084 | vascular endothelial growth factor signaling pathway | 0.125 | 0.011646448 | 0.031533796 | 22339 | 1 |
| 449 | Biological Process | GO:0060512 | prostate gland morphogenesis | 0.125 | 0.011646448 | 0.031533796 | 16000 | 1 |
| 450 | Biological Process | GO:0070873 | regulation of glycogen metabolic process | 0.125 | 0.011646448 | 0.031533796 | 16000 | 1 |
| 451 | Biological Process | GO:0000186 | activation of MAPKK activity | 0.125 | 0.011987186 | 0.032027437 | 13649 | 1 |
| 452 | Biological Process | GO:0045940 | positive regulation of steroid metabolic process | 0.125 | 0.011987186 | 0.032027437 | 16000 | 1 |
| 453 | Biological Process | GO:0046686 | response to cadmium ion | 0.125 | 0.011987186 | 0.032027437 | 13649 | 1 |
| 454 | Biological Process | GO:1903580 | positive regulation of ATP metabolic process | 0.125 | 0.011987186 | 0.032027437 | 16000 | 1 |
| 455 | Biological Process | GO:0010661 | positive regulation of muscle cell apoptotic process | 0.125 | 0.012327821 | 0.032507928 | 16009 | 1 |
| 456 | Biological Process | GO:0032885 | regulation of polysaccharide biosynthetic process | 0.125 | 0.012327821 | 0.032507928 | 16000 | 1 |
| 457 | Biological Process | GO:0035886 | vascular smooth muscle cell differentiation | 0.125 | 0.012327821 | 0.032507928 | 22339 | 1 |
| 458 | Biological Process | GO:0045601 | regulation of endothelial cell differentiation | 0.125 | 0.012327821 | 0.032507928 | 22339 | 1 |
| 459 | Biological Process | GO:0046326 | positive regulation of glucose import | 0.125 | 0.012327821 | 0.032507928 | 16000 | 1 |
| 460 | Biological Process | GO:0051968 | positive regulation of synaptic transmission, glutamatergic | 0.125 | 0.012327821 | 0.032507928 | 13649 | 1 |
| 461 | Biological Process | GO:0002053 | positive regulation of mesenchymal cell proliferation | 0.125 | 0.012668354 | 0.032556595 | 22339 | 1 |
| 462 | Biological Process | GO:0007435 | salivary gland morphogenesis | 0.125 | 0.012668354 | 0.032556595 | 13649 | 1 |
| 463 | Biological Process | GO:0030225 | macrophage differentiation | 0.125 | 0.012668354 | 0.032556595 | 22339 | 1 |
| 464 | Biological Process | GO:0033144 | negative regulation of intracellular steroid hormone receptor signaling pathway | 0.125 | 0.012668354 | 0.032556595 | 16000 | 1 |
| 465 | Biological Process | GO:0040019 | positive regulation of embryonic development | 0.125 | 0.012668354 | 0.032556595 | 16000 | 1 |
| 466 | Biological Process | GO:0048255 | mRNA stabilization | 0.125 | 0.012668354 | 0.032556595 | 22339 | 1 |
| 467 | Biological Process | GO:0048846 | axon extension involved in axon guidance | 0.125 | 0.012668354 | 0.032556595 | 22339 | 1 |
| 468 | Biological Process | GO:0061900 | glial cell activation | 0.125 | 0.012668354 | 0.032556595 | 13649 | 1 |
| 469 | Biological Process | GO:0071320 | cellular response to cAMP | 0.125 | 0.012668354 | 0.032556595 | 16011 | 1 |
| 470 | Biological Process | GO:1902284 | neuron projection extension involved in neuron projection guidance | 0.125 | 0.012668354 | 0.032556595 | 22339 | 1 |
| 471 | Biological Process | GO:1902692 | regulation of neuroblast proliferation | 0.125 | 0.012668354 | 0.032556595 | 22339 | 1 |
| 472 | Biological Process | GO:1905209 | positive regulation of cardiocyte differentiation | 0.125 | 0.012668354 | 0.032556595 | 16000 | 1 |
| 473 | Biological Process | GO:0009620 | response to fungus | 0.125 | 0.013008783 | 0.032737872 | 14825 | 1 |
| 474 | Biological Process | GO:0043403 | skeletal muscle tissue regeneration | 0.125 | 0.013008783 | 0.032737872 | 16000 | 1 |
| 475 | Biological Process | GO:0046006 | regulation of activated T cell proliferation | 0.125 | 0.013008783 | 0.032737872 | 16000 | 1 |
| 476 | Biological Process | GO:0046885 | regulation of hormone biosynthetic process | 0.125 | 0.013008783 | 0.032737872 | 16000 | 1 |
| 477 | Biological Process | GO:0060251 | regulation of glial cell proliferation | 0.125 | 0.013008783 | 0.032737872 | 16000 | 1 |
| 478 | Biological Process | GO:0070884 | regulation of calcineurin-NFAT signaling cascade | 0.125 | 0.013008783 | 0.032737872 | 16000 | 1 |
| 479 | Biological Process | GO:0071542 | dopaminergic neuron differentiation | 0.125 | 0.013008783 | 0.032737872 | 22339 | 1 |
| 480 | Biological Process | GO:0106056 | regulation of calcineurin-mediated signaling | 0.125 | 0.013008783 | 0.032737872 | 16000 | 1 |
| 481 | Biological Process | GO:0150076 | neuroinflammatory response | 0.125 | 0.013008783 | 0.032737872 | 13649 | 1 |
| 482 | Biological Process | GO:1905332 | positive regulation of morphogenesis of an epithelium | 0.125 | 0.013008783 | 0.032737872 | 22339 | 1 |
| 483 | Biological Process | GO:0003298 | physiological muscle hypertrophy | 0.125 | 0.013349111 | 0.032844769 | 16000 | 1 |
| 484 | Biological Process | GO:0003301 | physiological cardiac muscle hypertrophy | 0.125 | 0.013349111 | 0.032844769 | 16000 | 1 |
| 485 | Biological Process | GO:0006110 | regulation of glycolytic process | 0.125 | 0.013349111 | 0.032844769 | 16000 | 1 |
| 486 | Biological Process | GO:0010613 | positive regulation of cardiac muscle hypertrophy | 0.125 | 0.013349111 | 0.032844769 | 16000 | 1 |
| 487 | Biological Process | GO:0014742 | positive regulation of muscle hypertrophy | 0.125 | 0.013349111 | 0.032844769 | 16000 | 1 |
| 488 | Biological Process | GO:0030811 | regulation of nucleotide catabolic process | 0.125 | 0.013349111 | 0.032844769 | 16000 | 1 |
| 489 | Biological Process | GO:0031640 | killing of cells of other organism | 0.125 | 0.013349111 | 0.032844769 | 14825 | 1 |
| 490 | Biological Process | GO:0044364 | disruption of cells of other organism | 0.125 | 0.013349111 | 0.032844769 | 14825 | 1 |
| 491 | Biological Process | GO:0048730 | epidermis morphogenesis | 0.125 | 0.013349111 | 0.032844769 | 16011 | 1 |
| 492 | Biological Process | GO:0061049 | cell growth involved in cardiac muscle cell development | 0.125 | 0.013349111 | 0.032844769 | 16000 | 1 |
| 493 | Biological Process | GO:0090049 | regulation of cell migration involved in sprouting angiogenesis | 0.125 | 0.013349111 | 0.032844769 | 22339 | 1 |
| 494 | Biological Process | GO:0010559 | regulation of glycoprotein biosynthetic process | 0.125 | 0.013689335 | 0.033343701 | 16000 | 1 |
| 495 | Biological Process | GO:0034105 | positive regulation of tissue remodeling | 0.125 | 0.013689335 | 0.033343701 | 13649 | 1 |
| 496 | Biological Process | GO:0071364 | cellular response to epidermal growth factor stimulus | 0.125 | 0.013689335 | 0.033343701 | 13649 | 1 |
| 497 | Biological Process | GO:1900744 | regulation of p38MAPK cascade | 0.125 | 0.013689335 | 0.033343701 | 22339 | 1 |
| 498 | Biological Process | GO:1905953 | negative regulation of lipid localization | 0.125 | 0.013689335 | 0.033343701 | 16009 | 1 |
| 499 | Biological Process | GO:0007431 | salivary gland development | 0.125 | 0.014029457 | 0.033832467 | 13649 | 1 |
| 500 | Biological Process | GO:0045981 | positive regulation of nucleotide metabolic process | 0.125 | 0.014029457 | 0.033832467 | 16000 | 1 |
| 501 | Biological Process | GO:0055023 | positive regulation of cardiac muscle tissue growth | 0.125 | 0.014029457 | 0.033832467 | 16000 | 1 |
| 502 | Biological Process | GO:1900544 | positive regulation of purine nucleotide metabolic process | 0.125 | 0.014029457 | 0.033832467 | 16000 | 1 |
| 503 | Biological Process | GO:1903672 | positive regulation of sprouting angiogenesis | 0.125 | 0.014029457 | 0.033832467 | 22339 | 1 |
| 504 | Biological Process | GO:0005978 | glycogen biosynthetic process | 0.125 | 0.014369476 | 0.034043309 | 16000 | 1 |
| 505 | Biological Process | GO:0009250 | glucan biosynthetic process | 0.125 | 0.014369476 | 0.034043309 | 16000 | 1 |
| 506 | Biological Process | GO:0010765 | positive regulation of sodium ion transport | 0.125 | 0.014369476 | 0.034043309 | 14825 | 1 |
| 507 | Biological Process | GO:0010907 | positive regulation of glucose metabolic process | 0.125 | 0.014369476 | 0.034043309 | 16000 | 1 |
| 508 | Biological Process | GO:0032768 | regulation of monooxygenase activity | 0.125 | 0.014369476 | 0.034043309 | 13649 | 1 |
| 509 | Biological Process | GO:0032881 | regulation of polysaccharide metabolic process | 0.125 | 0.014369476 | 0.034043309 | 16000 | 1 |
| 510 | Biological Process | GO:0042462 | eye photoreceptor cell development | 0.125 | 0.014369476 | 0.034043309 | 22339 | 1 |
| 511 | Biological Process | GO:0050918 | positive chemotaxis | 0.125 | 0.014369476 | 0.034043309 | 22339 | 1 |
| 512 | Biological Process | GO:0060964 | regulation of gene silencing by miRNA | 0.125 | 0.014369476 | 0.034043309 | 13649 | 1 |
| 513 | Biological Process | GO:0001569 | branching involved in blood vessel morphogenesis | 0.125 | 0.014709392 | 0.034246628 | 22339 | 1 |
| 514 | Biological Process | GO:0002888 | positive regulation of myeloid leukocyte mediated immunity | 0.125 | 0.014709392 | 0.034246628 | 14825 | 1 |
| 515 | Biological Process | GO:0014904 | myotube cell development | 0.125 | 0.014709392 | 0.034246628 | 16000 | 1 |
| 516 | Biological Process | GO:0030166 | proteoglycan biosynthetic process | 0.125 | 0.014709392 | 0.034246628 | 16000 | 1 |
| 517 | Biological Process | GO:0043489 | RNA stabilization | 0.125 | 0.014709392 | 0.034246628 | 22339 | 1 |
| 518 | Biological Process | GO:0045740 | positive regulation of DNA replication | 0.125 | 0.014709392 | 0.034246628 | 13649 | 1 |
| 519 | Biological Process | GO:0048873 | homeostasis of number of cells within a tissue | 0.125 | 0.014709392 | 0.034246628 | 22339 | 1 |
| 520 | Biological Process | GO:0050974 | detection of mechanical stimulus involved in sensory perception | 0.125 | 0.014709392 | 0.034246628 | 16000 | 1 |
| 521 | Biological Process | GO:0070849 | response to epidermal growth factor | 0.125 | 0.014709392 | 0.034246628 | 13649 | 1 |
| 522 | Biological Process | GO:0060421 | positive regulation of heart growth | 0.125 | 0.015049206 | 0.034903800 | 16000 | 1 |
| 523 | Biological Process | GO:0090184 | positive regulation of kidney development | 0.125 | 0.015049206 | 0.034903800 | 22339 | 1 |
| 524 | Biological Process | GO:0010464 | regulation of mesenchymal cell proliferation | 0.125 | 0.015388918 | 0.035022059 | 22339 | 1 |
| 525 | Biological Process | GO:0031952 | regulation of protein autophosphorylation | 0.125 | 0.015388918 | 0.035022059 | 22339 | 1 |
| 526 | Biological Process | GO:0035196 | production of miRNAs involved in gene silencing by miRNA | 0.125 | 0.015388918 | 0.035022059 | 13649 | 1 |
| 527 | Biological Process | GO:0048010 | vascular endothelial growth factor receptor signaling pathway | 0.125 | 0.015388918 | 0.035022059 | 22339 | 1 |
| 528 | Biological Process | GO:0060147 | regulation of posttranscriptional gene silencing | 0.125 | 0.015388918 | 0.035022059 | 13649 | 1 |
| 529 | Biological Process | GO:0060966 | regulation of gene silencing by RNA | 0.125 | 0.015388918 | 0.035022059 | 13649 | 1 |
| 530 | Biological Process | GO:0090199 | regulation of release of cytochrome c from mitochondria | 0.125 | 0.015388918 | 0.035022059 | 16000 | 1 |
| 531 | Biological Process | GO:1902373 | negative regulation of mRNA catabolic process | 0.125 | 0.015388918 | 0.035022059 | 22339 | 1 |
| 532 | Biological Process | GO:1902667 | regulation of axon guidance | 0.125 | 0.015388918 | 0.035022059 | 22339 | 1 |
| 533 | Biological Process | GO:1903018 | regulation of glycoprotein metabolic process | 0.125 | 0.015388918 | 0.035022059 | 16000 | 1 |
| 534 | Biological Process | GO:0010828 | positive regulation of glucose transmembrane transport | 0.125 | 0.015728527 | 0.035462273 | 16000 | 1 |
| 535 | Biological Process | GO:0033173 | calcineurin-NFAT signaling cascade | 0.125 | 0.015728527 | 0.035462273 | 16000 | 1 |
| 536 | Biological Process | GO:0045907 | positive regulation of vasoconstriction | 0.125 | 0.015728527 | 0.035462273 | 13649 | 1 |
| 537 | Biological Process | GO:0050798 | activated T cell proliferation | 0.125 | 0.015728527 | 0.035462273 | 16000 | 1 |
| 538 | Biological Process | GO:0090311 | regulation of protein deacetylation | 0.125 | 0.015728527 | 0.035462273 | 22339 | 1 |
| 539 | Biological Process | GO:0000060 | protein import into nucleus, translocation | 0.125 | 0.016068033 | 0.035566650 | 16000 | 1 |
| 540 | Biological Process | GO:0014009 | glial cell proliferation | 0.125 | 0.016068033 | 0.035566650 | 16000 | 1 |
| 541 | Biological Process | GO:0014888 | striated muscle adaptation | 0.125 | 0.016068033 | 0.035566650 | 16011 | 1 |
| 542 | Biological Process | GO:0031050 | dsRNA processing | 0.125 | 0.016068033 | 0.035566650 | 13649 | 1 |
| 543 | Biological Process | GO:0038066 | p38MAPK cascade | 0.125 | 0.016068033 | 0.035566650 | 22339 | 1 |
| 544 | Biological Process | GO:0042088 | T-helper 1 type immune response | 0.125 | 0.016068033 | 0.035566650 | 22339 | 1 |
| 545 | Biological Process | GO:0043470 | regulation of carbohydrate catabolic process | 0.125 | 0.016068033 | 0.035566650 | 16000 | 1 |
| 546 | Biological Process | GO:0070918 | production of small RNA involved in gene silencing by RNA | 0.125 | 0.016068033 | 0.035566650 | 13649 | 1 |
| 547 | Biological Process | GO:1904036 | negative regulation of epithelial cell apoptotic process | 0.125 | 0.016068033 | 0.035566650 | 16000 | 1 |
| 548 | Biological Process | GO:2000725 | regulation of cardiac muscle cell differentiation | 0.125 | 0.016068033 | 0.035566650 | 16000 | 1 |
| 549 | Biological Process | GO:0030521 | androgen receptor signaling pathway | 0.125 | 0.016407437 | 0.036185857 | 16000 | 1 |
| 550 | Biological Process | GO:0051196 | regulation of coenzyme metabolic process | 0.125 | 0.016407437 | 0.036185857 | 16000 | 1 |
| 551 | Biological Process | GO:0010665 | regulation of cardiac muscle cell apoptotic process | 0.125 | 0.016746739 | 0.036601430 | 16009 | 1 |
| 552 | Biological Process | GO:0030104 | water homeostasis | 0.125 | 0.016746739 | 0.036601430 | 16000 | 1 |
| 553 | Biological Process | GO:0048599 | oocyte development | 0.125 | 0.016746739 | 0.036601430 | 16000 | 1 |
| 554 | Biological Process | GO:0050850 | positive regulation of calcium-mediated signaling | 0.125 | 0.016746739 | 0.036601430 | 16000 | 1 |
| 555 | Biological Process | GO:0097720 | calcineurin-mediated signaling | 0.125 | 0.016746739 | 0.036601430 | 16000 | 1 |
| 556 | Biological Process | GO:0035924 | cellular response to vascular endothelial growth factor stimulus | 0.125 | 0.017085938 | 0.037009361 | 22339 | 1 |
| 557 | Biological Process | GO:0043268 | positive regulation of potassium ion transport | 0.125 | 0.017085938 | 0.037009361 | 14825 | 1 |
| 558 | Biological Process | GO:0060428 | lung epithelium development | 0.125 | 0.017085938 | 0.037009361 | 16000 | 1 |
| 559 | Biological Process | GO:2000677 | regulation of transcription regulatory region DNA binding | 0.125 | 0.017085938 | 0.037009361 | 16000 | 1 |
| 560 | Biological Process | GO:2001169 | regulation of ATP biosynthetic process | 0.125 | 0.017085938 | 0.037009361 | 16000 | 1 |
| 561 | Biological Process | GO:0010823 | negative regulation of mitochondrion organization | 0.125 | 0.017425034 | 0.037542747 | 16000 | 1 |
| 562 | Biological Process | GO:0032355 | response to estradiol | 0.125 | 0.017425034 | 0.037542747 | 13649 | 1 |
| 563 | Biological Process | GO:0043618 | regulation of transcription from RNA polymerase II promoter in response to stress | 0.125 | 0.017425034 | 0.037542747 | 22339 | 1 |
| 564 | Biological Process | GO:0001954 | positive regulation of cell-matrix adhesion | 0.125 | 0.017764029 | 0.037936209 | 22339 | 1 |
| 565 | Biological Process | GO:0002833 | positive regulation of response to biotic stimulus | 0.125 | 0.017764029 | 0.037936209 | 14825 | 1 |
| 566 | Biological Process | GO:0010662 | regulation of striated muscle cell apoptotic process | 0.125 | 0.017764029 | 0.037936209 | 16009 | 1 |
| 567 | Biological Process | GO:0045599 | negative regulation of fat cell differentiation | 0.125 | 0.017764029 | 0.037936209 | 22339 | 1 |
| 568 | Biological Process | GO:0045773 | positive regulation of axon extension | 0.125 | 0.017764029 | 0.037936209 | 22339 | 1 |
| 569 | Biological Process | GO:0009994 | oocyte differentiation | 0.125 | 0.018102921 | 0.038056920 | 16000 | 1 |
| 570 | Biological Process | GO:0010659 | cardiac muscle cell apoptotic process | 0.125 | 0.018102921 | 0.038056920 | 16009 | 1 |
| 571 | Biological Process | GO:0030850 | prostate gland development | 0.125 | 0.018102921 | 0.038056920 | 16000 | 1 |
| 572 | Biological Process | GO:0032350 | regulation of hormone metabolic process | 0.125 | 0.018102921 | 0.038056920 | 16000 | 1 |
| 573 | Biological Process | GO:0048247 | lymphocyte chemotaxis | 0.125 | 0.018102921 | 0.038056920 | 20299 | 1 |
| 574 | Biological Process | GO:0048546 | digestive tract morphogenesis | 0.125 | 0.018102921 | 0.038056920 | 13649 | 1 |
| 575 | Biological Process | GO:0050732 | negative regulation of peptidyl-tyrosine phosphorylation | 0.125 | 0.018102921 | 0.038056920 | 16000 | 1 |
| 576 | Biological Process | GO:0061383 | trabecula morphogenesis | 0.125 | 0.018102921 | 0.038056920 | 22339 | 1 |
| 577 | Biological Process | GO:1902369 | negative regulation of RNA catabolic process | 0.125 | 0.018102921 | 0.038056920 | 22339 | 1 |
| 578 | Biological Process | GO:0010656 | negative regulation of muscle cell apoptotic process | 0.125 | 0.018441710 | 0.038370145 | 16000 | 1 |
| 579 | Biological Process | GO:0042246 | tissue regeneration | 0.125 | 0.018441710 | 0.038370145 | 16000 | 1 |
| 580 | Biological Process | GO:0042307 | positive regulation of protein import into nucleus | 0.125 | 0.018441710 | 0.038370145 | 16000 | 1 |
| 581 | Biological Process | GO:0042531 | positive regulation of tyrosine phosphorylation of STAT protein | 0.125 | 0.018441710 | 0.038370145 | 16000 | 1 |
| 582 | Biological Process | GO:0048016 | inositol phosphate-mediated signaling | 0.125 | 0.018441710 | 0.038370145 | 16000 | 1 |
| 583 | Biological Process | GO:0055025 | positive regulation of cardiac muscle tissue development | 0.125 | 0.018441710 | 0.038370145 | 16000 | 1 |
| 584 | Biological Process | GO:0002042 | cell migration involved in sprouting angiogenesis | 0.125 | 0.018780398 | 0.038874782 | 22339 | 1 |
| 585 | Biological Process | GO:0002548 | monocyte chemotaxis | 0.125 | 0.018780398 | 0.038874782 | 20299 | 1 |
| 586 | Biological Process | GO:0021795 | cerebral cortex cell migration | 0.125 | 0.018780398 | 0.038874782 | 13649 | 1 |
| 587 | Biological Process | GO:0001754 | eye photoreceptor cell differentiation | 0.125 | 0.019118983 | 0.039108475 | 22339 | 1 |
| 588 | Biological Process | GO:0010658 | striated muscle cell apoptotic process | 0.125 | 0.019118983 | 0.039108475 | 16009 | 1 |
| 589 | Biological Process | GO:0043620 | regulation of DNA-templated transcription in response to stress | 0.125 | 0.019118983 | 0.039108475 | 22339 | 1 |
| 590 | Biological Process | GO:0046622 | positive regulation of organ growth | 0.125 | 0.019118983 | 0.039108475 | 16000 | 1 |
| 591 | Biological Process | GO:0048260 | positive regulation of receptor-mediated endocytosis | 0.125 | 0.019118983 | 0.039108475 | 22339 | 1 |
| 592 | Biological Process | GO:0051893 | regulation of focal adhesion assembly | 0.125 | 0.019118983 | 0.039108475 | 22339 | 1 |
| 593 | Biological Process | GO:0090109 | regulation of cell-substrate junction assembly | 0.125 | 0.019118983 | 0.039108475 | 22339 | 1 |
| 594 | Biological Process | GO:0010463 | mesenchymal cell proliferation | 0.125 | 0.019457465 | 0.039733848 | 22339 | 1 |
| 595 | Biological Process | GO:0001836 | release of cytochrome c from mitochondria | 0.125 | 0.019795846 | 0.040154450 | 16000 | 1 |
| 596 | Biological Process | GO:0050982 | detection of mechanical stimulus | 0.125 | 0.019795846 | 0.040154450 | 16000 | 1 |
| 597 | Biological Process | GO:0060443 | mammary gland morphogenesis | 0.125 | 0.019795846 | 0.040154450 | 16011 | 1 |
| 598 | Biological Process | GO:0090183 | regulation of kidney development | 0.125 | 0.019795846 | 0.040154450 | 22339 | 1 |
| 599 | Biological Process | GO:0042698 | ovulation cycle | 0.125 | 0.020134125 | 0.040636761 | 13649 | 1 |
| 600 | Biological Process | GO:1903078 | positive regulation of protein localization to plasma membrane | 0.125 | 0.020134125 | 0.040636761 | 13649 | 1 |
| 601 | Biological Process | GO:1904591 | positive regulation of protein import | 0.125 | 0.020134125 | 0.040636761 | 16000 | 1 |
| 602 | Biological Process | GO:0042461 | photoreceptor cell development | 0.125 | 0.020472301 | 0.041046117 | 22339 | 1 |
| 603 | Biological Process | GO:0045840 | positive regulation of mitotic nuclear division | 0.125 | 0.020472301 | 0.041046117 | 16000 | 1 |
| 604 | Biological Process | GO:0046579 | positive regulation of Ras protein signal transduction | 0.125 | 0.020472301 | 0.041046117 | 16000 | 1 |
| 605 | Biological Process | GO:0048008 | platelet-derived growth factor receptor signaling pathway | 0.125 | 0.020472301 | 0.041046117 | 22339 | 1 |
| 606 | Biological Process | GO:0007595 | lactation | 0.125 | 0.020810375 | 0.041449893 | 22339 | 1 |
| 607 | Biological Process | GO:0045600 | positive regulation of fat cell differentiation | 0.125 | 0.020810375 | 0.041449893 | 16000 | 1 |
| 608 | Biological Process | GO:0045668 | negative regulation of osteoblast differentiation | 0.125 | 0.020810375 | 0.041449893 | 16011 | 1 |
| 609 | Biological Process | GO:0045739 | positive regulation of DNA repair | 0.125 | 0.020810375 | 0.041449893 | 13649 | 1 |
| 610 | Biological Process | GO:0046324 | regulation of glucose import | 0.125 | 0.021148347 | 0.041985180 | 16000 | 1 |
| 611 | Biological Process | GO:1903391 | regulation of adherens junction organization | 0.125 | 0.021148347 | 0.041985180 | 22339 | 1 |
| 612 | Biological Process | GO:0002886 | regulation of myeloid leukocyte mediated immunity | 0.125 | 0.021486217 | 0.042104655 | 14825 | 1 |
| 613 | Biological Process | GO:0010676 | positive regulation of cellular carbohydrate metabolic process | 0.125 | 0.021486217 | 0.042104655 | 16000 | 1 |
| 614 | Biological Process | GO:0030808 | regulation of nucleotide biosynthetic process | 0.125 | 0.021486217 | 0.042104655 | 16000 | 1 |
| 615 | Biological Process | GO:0033692 | cellular polysaccharide biosynthetic process | 0.125 | 0.021486217 | 0.042104655 | 16000 | 1 |
| 616 | Biological Process | GO:0043388 | positive regulation of DNA binding | 0.125 | 0.021486217 | 0.042104655 | 16000 | 1 |
| 617 | Biological Process | GO:0045661 | regulation of myoblast differentiation | 0.125 | 0.021486217 | 0.042104655 | 16009 | 1 |
| 618 | Biological Process | GO:1900371 | regulation of purine nucleotide biosynthetic process | 0.125 | 0.021486217 | 0.042104655 | 16000 | 1 |
| 619 | Biological Process | GO:2000242 | negative regulation of reproductive process | 0.125 | 0.021486217 | 0.042104655 | 16000 | 1 |
| 620 | Biological Process | GO:0001658 | branching involved in ureteric bud morphogenesis | 0.125 | 0.021823985 | 0.042560280 | 22339 | 1 |
| 621 | Biological Process | GO:0051193 | regulation of cofactor metabolic process | 0.125 | 0.021823985 | 0.042560280 | 16000 | 1 |
| 622 | Biological Process | GO:0097194 | execution phase of apoptosis | 0.125 | 0.021823985 | 0.042560280 | 16009 | 1 |
| 623 | Biological Process | GO:0051591 | response to cAMP | 0.125 | 0.022161651 | 0.043080261 | 16011 | 1 |
| 624 | Biological Process | GO:0060425 | lung morphogenesis | 0.125 | 0.022161651 | 0.043080261 | 16000 | 1 |
| 625 | Biological Process | GO:0007405 | neuroblast proliferation | 0.125 | 0.022499215 | 0.043388788 | 22339 | 1 |
| 626 | Biological Process | GO:0014068 | positive regulation of phosphatidylinositol 3-kinase signaling | 0.125 | 0.022499215 | 0.043388788 | 16000 | 1 |
| 627 | Biological Process | GO:0045428 | regulation of nitric oxide biosynthetic process | 0.125 | 0.022499215 | 0.043388788 | 16000 | 1 |
| 628 | Biological Process | GO:1901224 | positive regulation of NIK/NF-kappaB signaling | 0.125 | 0.022499215 | 0.043388788 | 13649 | 1 |
| 629 | Biological Process | GO:1904377 | positive regulation of protein localization to cell periphery | 0.125 | 0.022499215 | 0.043388788 | 13649 | 1 |
| 630 | Biological Process | GO:0002090 | regulation of receptor internalization | 0.125 | 0.022836677 | 0.043761278 | 22339 | 1 |
| 631 | Biological Process | GO:0006029 | proteoglycan metabolic process | 0.125 | 0.022836677 | 0.043761278 | 16000 | 1 |
| 632 | Biological Process | GO:0051057 | positive regulation of small GTPase mediated signal transduction | 0.125 | 0.022836677 | 0.043761278 | 16000 | 1 |
| 633 | Biological Process | GO:0060688 | regulation of morphogenesis of a branching structure | 0.125 | 0.022836677 | 0.043761278 | 22339 | 1 |
| 634 | Biological Process | GO:1903670 | regulation of sprouting angiogenesis | 0.125 | 0.023174037 | 0.044337708 | 22339 | 1 |
| 635 | Biological Process | GO:0001541 | ovarian follicle development | 0.125 | 0.023511295 | 0.044841511 | 22339 | 1 |
| 636 | Biological Process | GO:0042509 | regulation of tyrosine phosphorylation of STAT protein | 0.125 | 0.023511295 | 0.044841511 | 16000 | 1 |
| 637 | Biological Process | GO:0000271 | polysaccharide biosynthetic process | 0.125 | 0.023848451 | 0.045129753 | 16000 | 1 |
| 638 | Biological Process | GO:0010611 | regulation of cardiac muscle hypertrophy | 0.125 | 0.023848451 | 0.045129753 | 16000 | 1 |
| 639 | Biological Process | GO:0016575 | histone deacetylation | 0.125 | 0.023848451 | 0.045129753 | 22339 | 1 |
| 640 | Biological Process | GO:0022029 | telencephalon cell migration | 0.125 | 0.023848451 | 0.045129753 | 13649 | 1 |
| 641 | Biological Process | GO:0060675 | ureteric bud morphogenesis | 0.125 | 0.023848451 | 0.045129753 | 22339 | 1 |
| 642 | Biological Process | GO:0046323 | glucose import | 0.125 | 0.024185506 | 0.045413341 | 16000 | 1 |
| 643 | Biological Process | GO:0050810 | regulation of steroid biosynthetic process | 0.125 | 0.024185506 | 0.045413341 | 16000 | 1 |
| 644 | Biological Process | GO:0051851 | modification by host of symbiont morphology or physiology | 0.125 | 0.024185506 | 0.045413341 | 14825 | 1 |
| 645 | Biological Process | GO:0072171 | mesonephric tubule morphogenesis | 0.125 | 0.024185506 | 0.045413341 | 22339 | 1 |
| 646 | Biological Process | GO:0097755 | positive regulation of blood vessel diameter | 0.125 | 0.024185506 | 0.045413341 | 13649 | 1 |
| 647 | Biological Process | GO:0042446 | hormone biosynthetic process | 0.125 | 0.024522458 | 0.045974871 | 16000 | 1 |
| 648 | Biological Process | GO:0007045 | cell-substrate adherens junction assembly | 0.125 | 0.024859309 | 0.046037163 | 22339 | 1 |
| 649 | Biological Process | GO:0007260 | tyrosine phosphorylation of STAT protein | 0.125 | 0.024859309 | 0.046037163 | 16000 | 1 |
| 650 | Biological Process | GO:0021885 | forebrain cell migration | 0.125 | 0.024859309 | 0.046037163 | 13649 | 1 |
| 651 | Biological Process | GO:0048041 | focal adhesion assembly | 0.125 | 0.024859309 | 0.046037163 | 22339 | 1 |
| 652 | Biological Process | GO:0048844 | artery morphogenesis | 0.125 | 0.024859309 | 0.046037163 | 22339 | 1 |
| 653 | Biological Process | GO:0051145 | smooth muscle cell differentiation | 0.125 | 0.024859309 | 0.046037163 | 22339 | 1 |
| 654 | Biological Process | GO:0051155 | positive regulation of striated muscle cell differentiation | 0.125 | 0.024859309 | 0.046037163 | 16000 | 1 |
| 655 | Biological Process | GO:2000573 | positive regulation of DNA biosynthetic process | 0.125 | 0.024859309 | 0.046037163 | 22339 | 1 |
| 656 | Biological Process | GO:0006809 | nitric oxide biosynthetic process | 0.125 | 0.025196058 | 0.046307300 | 16000 | 1 |
| 657 | Biological Process | GO:0034637 | cellular carbohydrate biosynthetic process | 0.125 | 0.025196058 | 0.046307300 | 16000 | 1 |
| 658 | Biological Process | GO:0051966 | regulation of synaptic transmission, glutamatergic | 0.125 | 0.025196058 | 0.046307300 | 13649 | 1 |
| 659 | Biological Process | GO:0055021 | regulation of cardiac muscle tissue growth | 0.125 | 0.025196058 | 0.046307300 | 16000 | 1 |
| 660 | Biological Process | GO:0071347 | cellular response to interleukin-1 | 0.125 | 0.025196058 | 0.046307300 | 20299 | 1 |
| 661 | Biological Process | GO:0019229 | regulation of vasoconstriction | 0.125 | 0.025532705 | 0.046643330 | 13649 | 1 |
| 662 | Biological Process | GO:0046824 | positive regulation of nucleocytoplasmic transport | 0.125 | 0.025532705 | 0.046643330 | 16000 | 1 |
| 663 | Biological Process | GO:0070373 | negative regulation of ERK1 and ERK2 cascade | 0.125 | 0.025532705 | 0.046643330 | 16000 | 1 |
| 664 | Biological Process | GO:1903578 | regulation of ATP metabolic process | 0.125 | 0.025532705 | 0.046643330 | 16000 | 1 |
| 665 | Biological Process | GO:0005977 | glycogen metabolic process | 0.125 | 0.025869250 | 0.046765128 | 16000 | 1 |
| 666 | Biological Process | GO:0006073 | cellular glucan metabolic process | 0.125 | 0.025869250 | 0.046765128 | 16000 | 1 |
| 667 | Biological Process | GO:0044042 | glucan metabolic process | 0.125 | 0.025869250 | 0.046765128 | 16000 | 1 |
| 668 | Biological Process | GO:0051702 | interaction with symbiont | 0.125 | 0.025869250 | 0.046765128 | 14825 | 1 |
| 669 | Biological Process | GO:0051785 | positive regulation of nuclear division | 0.125 | 0.025869250 | 0.046765128 | 16000 | 1 |
| 670 | Biological Process | GO:1901862 | negative regulation of muscle tissue development | 0.125 | 0.025869250 | 0.046765128 | 16011 | 1 |
| 671 | Biological Process | GO:1903312 | negative regulation of mRNA metabolic process | 0.125 | 0.025869250 | 0.046765128 | 22339 | 1 |
| 672 | Biological Process | GO:0046209 | nitric oxide metabolic process | 0.125 | 0.026205694 | 0.047162473 | 16000 | 1 |
| 673 | Biological Process | GO:0048747 | muscle fiber development | 0.125 | 0.026205694 | 0.047162473 | 22339 | 1 |
| 674 | Biological Process | GO:0051781 | positive regulation of cell division | 0.125 | 0.026205694 | 0.047162473 | 22339 | 1 |
| 675 | Biological Process | GO:0006096 | glycolytic process | 0.125 | 0.026542036 | 0.047416038 | 16000 | 1 |
| 676 | Biological Process | GO:0033143 | regulation of intracellular steroid hormone receptor signaling pathway | 0.125 | 0.026542036 | 0.047416038 | 16000 | 1 |
| 677 | Biological Process | GO:0048708 | astrocyte differentiation | 0.125 | 0.026542036 | 0.047416038 | 13649 | 1 |
| 678 | Biological Process | GO:0060976 | coronary vasculature development | 0.125 | 0.026542036 | 0.047416038 | 22339 | 1 |
| 679 | Biological Process | GO:0071674 | mononuclear cell migration | 0.125 | 0.026542036 | 0.047416038 | 20299 | 1 |
| 680 | Biological Process | GO:0006757 | ATP generation from ADP | 0.125 | 0.026878277 | 0.047596131 | 16000 | 1 |
| 681 | Biological Process | GO:0038034 | signal transduction in absence of ligand | 0.125 | 0.026878277 | 0.047596131 | 16000 | 1 |
| 682 | Biological Process | GO:0045913 | positive regulation of carbohydrate metabolic process | 0.125 | 0.026878277 | 0.047596131 | 16000 | 1 |
| 683 | Biological Process | GO:0051341 | regulation of oxidoreductase activity | 0.125 | 0.026878277 | 0.047596131 | 13649 | 1 |
| 684 | Biological Process | GO:0072078 | nephron tubule morphogenesis | 0.125 | 0.026878277 | 0.047596131 | 22339 | 1 |
| 685 | Biological Process | GO:0097192 | extrinsic apoptotic signaling pathway in absence of ligand | 0.125 | 0.026878277 | 0.047596131 | 16000 | 1 |
| 686 | Biological Process | GO:0046530 | photoreceptor cell differentiation | 0.125 | 0.027214415 | 0.048121116 | 22339 | 1 |
| 687 | Biological Process | GO:0034333 | adherens junction assembly | 0.125 | 0.027550453 | 0.048362806 | 22339 | 1 |
| 688 | Biological Process | GO:0042306 | regulation of protein import into nucleus | 0.125 | 0.027550453 | 0.048362806 | 16000 | 1 |
| 689 | Biological Process | GO:0060420 | regulation of heart growth | 0.125 | 0.027550453 | 0.048362806 | 16000 | 1 |
| 690 | Biological Process | GO:0072088 | nephron epithelium morphogenesis | 0.125 | 0.027550453 | 0.048362806 | 22339 | 1 |
| 691 | Biological Process | GO:1901888 | regulation of cell junction assembly | 0.125 | 0.027550453 | 0.048362806 | 22339 | 1 |
| 692 | Biological Process | GO:0042866 | pyruvate biosynthetic process | 0.125 | 0.027886388 | 0.048670775 | 16000 | 1 |
| 693 | Biological Process | GO:0045471 | response to ethanol | 0.125 | 0.027886388 | 0.048670775 | 16000 | 1 |
| 694 | Biological Process | GO:0072028 | nephron morphogenesis | 0.125 | 0.027886388 | 0.048670775 | 22339 | 1 |
| 695 | Biological Process | GO:2001057 | reactive nitrogen species metabolic process | 0.125 | 0.027886388 | 0.048670775 | 16000 | 1 |
| 696 | Biological Process | GO:0003151 | outflow tract morphogenesis | 0.125 | 0.028222222 | 0.049045208 | 22339 | 1 |
| 697 | Biological Process | GO:0007044 | cell-substrate junction assembly | 0.125 | 0.028222222 | 0.049045208 | 22339 | 1 |
| 698 | Biological Process | GO:0010827 | regulation of glucose transmembrane transport | 0.125 | 0.028222222 | 0.049045208 | 16000 | 1 |
| 699 | Biological Process | GO:0014015 | positive regulation of gliogenesis | 0.125 | 0.028557955 | 0.049416261 | 16000 | 1 |
| 700 | Biological Process | GO:0045844 | positive regulation of striated muscle tissue development | 0.125 | 0.028557955 | 0.049416261 | 16000 | 1 |
| 701 | Biological Process | GO:0048636 | positive regulation of muscle organ development | 0.125 | 0.028557955 | 0.049416261 | 16000 | 1 |
| 702 | Biological Process | GO:0001912 | positive regulation of leukocyte mediated cytotoxicity | 0.125 | 0.028893586 | 0.049572729 | 14825 | 1 |
| 703 | Biological Process | GO:0002690 | positive regulation of leukocyte chemotaxis | 0.125 | 0.028893586 | 0.049572729 | 22339 | 1 |
| 704 | Biological Process | GO:0061333 | renal tubule morphogenesis | 0.125 | 0.028893586 | 0.049572729 | 22339 | 1 |
| 705 | Biological Process | GO:1900182 | positive regulation of protein localization to nucleus | 0.125 | 0.028893586 | 0.049572729 | 16000 | 1 |
| 706 | Biological Process | GO:1901863 | positive regulation of muscle tissue development | 0.125 | 0.028893586 | 0.049572729 | 16000 | 1 |
| 707 | Biological Process | GO:1904035 | regulation of epithelial cell apoptotic process | 0.125 | 0.028893586 | 0.049572729 | 16000 | 1 |
| 708 | Biological Process | GO:0006112 | energy reserve metabolic process | 0.125 | 0.029229115 | 0.049796232 | 16000 | 1 |
| 709 | Biological Process | GO:0010717 | regulation of epithelial to mesenchymal transition | 0.125 | 0.029229115 | 0.049796232 | 22339 | 1 |
| 710 | Biological Process | GO:0046031 | ADP metabolic process | 0.125 | 0.029229115 | 0.049796232 | 16000 | 1 |
| 711 | Biological Process | GO:1903524 | positive regulation of blood circulation | 0.125 | 0.029229115 | 0.049796232 | 13649 | 1 |
| 712 | Biological Process | GO:1904589 | regulation of protein import | 0.125 | 0.029229115 | 0.049796232 | 16000 | 1 |
| 1 | Molecular Function | GO:0019838 | growth factor binding | 0.625 | 0.000000001 | 0.000000014 | 16012/13649/16009/22339/16011 | 5 |
| 2 | Molecular Function | GO:0001968 | fibronectin binding | 0.5 | 0.000000000 | 0.000000009 | 16012/16009/22339/16011 | 4 |
| 3 | Molecular Function | GO:0048018 | receptor ligand activity | 0.5 | 0.000013249 | 0.000115729 | 16000/20299/22339/14825 | 4 |
| 4 | Molecular Function | GO:0031994 | insulin-like growth factor I binding | 0.375 | 0.000000006 | 0.000000100 | 16012/16009/16011 | 3 |
| 5 | Molecular Function | GO:0005520 | insulin-like growth factor binding | 0.375 | 0.000000093 | 0.000001118 | 16012/16009/16011 | 3 |
| 6 | Molecular Function | GO:0008083 | growth factor activity | 0.375 | 0.000014466 | 0.000115729 | 16000/22339/14825 | 3 |
| 7 | Molecular Function | GO:0005125 | cytokine activity | 0.375 | 0.000044425 | 0.000304626 | 20299/22339/14825 | 3 |
| 8 | Molecular Function | GO:0005126 | cytokine receptor binding | 0.375 | 0.000149355 | 0.000796561 | 20299/22339/14825 | 3 |
| 9 | Molecular Function | GO:0008009 | chemokine activity | 0.25 | 0.000097057 | 0.000582344 | 20299/14825 | 2 |
| 10 | Molecular Function | GO:0042379 | chemokine receptor binding | 0.25 | 0.000229620 | 0.001102176 | 20299/14825 | 2 |
| 11 | Molecular Function | GO:0005178 | integrin binding | 0.25 | 0.000782376 | 0.003414004 | 16000/13649 | 2 |
| 12 | Molecular Function | GO:0050839 | cell adhesion molecule binding | 0.25 | 0.002715509 | 0.010862037 | 16000/13649 | 2 |
| 13 | Molecular Function | GO:0001664 | G protein-coupled receptor binding | 0.25 | 0.004168257 | 0.013876332 | 20299/14825 | 2 |
| 14 | Molecular Function | GO:0008047 | enzyme activator activity | 0.25 | 0.009848833 | 0.022511617 | 16000/16009 | 2 |
| 15 | Molecular Function | GO:0019211 | phosphatase activator activity | 0.125 | 0.003863203 | 0.013876332 | 16009 | 1 |
| 16 | Molecular Function | GO:0005172 | vascular endothelial growth factor receptor binding | 0.125 | 0.004564198 | 0.013876332 | 22339 | 1 |
| 17 | Molecular Function | GO:0005159 | insulin-like growth factor receptor binding | 0.125 | 0.004914534 | 0.013876332 | 16000 | 1 |
| 18 | Molecular Function | GO:0005161 | platelet-derived growth factor receptor binding | 0.125 | 0.004914534 | 0.013876332 | 22339 | 1 |
| 19 | Molecular Function | GO:0038191 | neuropilin binding | 0.125 | 0.007014285 | 0.018704761 | 22339 | 1 |
| 20 | Molecular Function | GO:0043539 | protein serine/threonine kinase activator activity | 0.125 | 0.008411964 | 0.021251279 | 16000 | 1 |
| 21 | Molecular Function | GO:0005158 | insulin receptor binding | 0.125 | 0.009110158 | 0.021864380 | 16000 | 1 |
| 22 | Molecular Function | GO:0042056 | chemoattractant activity | 0.125 | 0.013637942 | 0.029755511 | 22339 | 1 |
| 23 | Molecular Function | GO:0048020 | CCR chemokine receptor binding | 0.125 | 0.016068474 | 0.033534206 | 20299 | 1 |
| 24 | Molecular Function | GO:0050840 | extracellular matrix binding | 0.125 | 0.020568417 | 0.040817500 | 22339 | 1 |
| 25 | Molecular Function | GO:0004714 | transmembrane receptor protein tyrosine kinase activity | 0.125 | 0.021259115 | 0.040817500 | 13649 | 1 |
| 26 | Molecular Function | GO:0019888 | protein phosphatase regulator activity | 0.125 | 0.023673203 | 0.043704375 | 16009 | 1 |
| 27 | Molecular Function | GO:0030295 | protein kinase activator activity | 0.125 | 0.025738273 | 0.045028305 | 16000 | 1 |
| 28 | Molecular Function | GO:0019199 | transmembrane receptor protein kinase activity | 0.125 | 0.027112862 | 0.045028305 | 13649 | 1 |
| 29 | Molecular Function | GO:0019208 | phosphatase regulator activity | 0.125 | 0.028142690 | 0.045028305 | 16009 | 1 |
| 30 | Molecular Function | GO:0019209 | kinase activator activity | 0.125 | 0.028142690 | 0.045028305 | 16000 | 1 |
| 1 | Cellular Component | GO:0062023 | collagen-containing extracellular matrix | 0.375 | 0.000147385 | 0.002063394 | 16012/16000/22339 | 3 |
| 2 | Cellular Component | GO:0030141 | secretory granule | 0.375 | 0.000223500 | 0.002086002 | 16000/16009/22339 | 3 |
| 3 | Cellular Component | GO:0031012 | extracellular matrix | 0.375 | 0.000427898 | 0.002995288 | 16012/16000/22339 | 3 |
| 4 | Cellular Component | GO:0031091 | platelet alpha granule | 0.25 | 0.000017415 | 0.000487611 | 16000/16009 | 2 |
| 5 | Cellular Component | GO:0005614 | interstitial matrix | 0.125 | 0.005794122 | 0.032447082 | 16000 | 1 |
| 6 | Cellular Component | GO:0005771 | multivesicular body | 0.125 | 0.013924043 | 0.048565798 | 13649 | 1 |
| 7 | Cellular Component | GO:0009925 | basal plasma membrane | 0.125 | 0.014261523 | 0.048565798 | 13649 | 1 |
| 8 | Cellular Component | GO:0005720 | nuclear heterochromatin | 0.125 | 0.014936181 | 0.048565798 | 16009 | 1 |
| 9 | Cellular Component | GO:0031901 | early endosome membrane | 0.125 | 0.015610435 | 0.048565798 | 13649 | 1 |
