# Supplementary material for: Huiyang Shengji decoction promotes wound healing in diabetic mice by activating the EGFR/PI3K/ATK pathway
Source: Chin Med. 2021 Nov 2;16:111. doi: 10.1186/s13020-021-00497-0 (PMC8565039; doi:10.1186/s13020-021-00497-0)
Supplement: Supplementary file 3 — Additional file 3: Table S3. GO analysis of the differentially-expressed proteins between the HYSJD and model group. [file 13020_2021_497_MOESM3_ESM.doc]

|  | **Table S3: GO analysis of the differentially-expressed proteins between the HYSJD and model group** | | | | | | | |
| --- | --- | --- | --- | --- | --- | --- | --- | --- |
|  | Gene Ontology Classification | ID | Description | GeneRatio | pvalue | p.adjust | geneID | Count |
| 1 | Biological Process | GO:0022612 | gland morphogenesis | 1 | 1.35E-09 | 1.26E-06 | Igf1/Ccl11/Egfr/Hgf | 4 |
| 2 | Biological Process | GO:0035272 | exocrine system development | 0.75 | 4.64E-08 | 2.17E-05 | Igf1/Egfr/Hgf | 3 |
| 3 | Biological Process | GO:0048732 | gland development | 1 | 1.37E-07 | 3.75E-05 | Igf1/Ccl11/Egfr/Hgf | 4 |
| 4 | Biological Process | GO:0030335 | positive regulation of cell migration | 1 | 1.82E-07 | 3.75E-05 | Igf1/Ccl11/Egfr/Hgf | 4 |
| 5 | Biological Process | GO:0014066 | regulation of phosphatidylinositol 3-kinase signaling | 0.75 | 2.01E-07 | 3.75E-05 | Igf1/Egfr/Hgf | 3 |
| 6 | Biological Process | GO:0014065 | phosphatidylinositol 3-kinase signaling | 0.75 | 4.83E-07 | 7.53E-05 | Igf1/Egfr/Hgf | 3 |
| 7 | Biological Process | GO:0048015 | phosphatidylinositol-mediated signaling | 0.75 | 8.24E-07 | 0.000102536 | Igf1/Egfr/Hgf | 3 |
| 8 | Biological Process | GO:0048017 | inositol lipid-mediated signaling | 0.75 | 8.77E-07 | 0.000102536 | Igf1/Egfr/Hgf | 3 |
| 9 | Biological Process | GO:0050679 | positive regulation of epithelial cell proliferation | 0.75 | 1.92E-06 | 0.000199333 | Igf1/Ccl11/Egfr | 3 |
| 10 | Biological Process | GO:0043406 | positive regulation of MAP kinase activity | 0.75 | 3.04E-06 | 0.000243556 | Igf1/Egfr/Hgf | 3 |
| 11 | Biological Process | GO:0061138 | morphogenesis of a branching epithelium | 0.75 | 3.04E-06 | 0.000243556 | Igf1/Ccl11/Hgf | 3 |
| 12 | Biological Process | GO:0090201 | negative regulation of release of cytochrome c from mitochondria | 0.5 | 3.61E-06 | 0.000243556 | Igf1/Hgf | 2 |
| 13 | Biological Process | GO:0051054 | positive regulation of DNA metabolic process | 0.75 | 3.80E-06 | 0.000243556 | Igf1/Egfr/Hgf | 3 |
| 14 | Biological Process | GO:0001763 | morphogenesis of a branching structure | 0.75 | 3.85E-06 | 0.000243556 | Igf1/Ccl11/Hgf | 3 |
| 15 | Biological Process | GO:0032147 | activation of protein kinase activity | 0.75 | 4.20E-06 | 0.000243556 | Igf1/Egfr/Hgf | 3 |
| 16 | Biological Process | GO:0050730 | regulation of peptidyl-tyrosine phosphorylation | 0.75 | 4.25E-06 | 0.000243556 | Igf1/Egfr/Hgf | 3 |
| 17 | Biological Process | GO:0051450 | myoblast proliferation | 0.5 | 4.43E-06 | 0.000243556 | Igf1/Hgf | 2 |
| 18 | Biological Process | GO:0043405 | regulation of MAP kinase activity | 0.75 | 7.06E-06 | 0.000366708 | Igf1/Egfr/Hgf | 3 |
| 19 | Biological Process | GO:0071902 | positive regulation of protein serine/threonine kinase activity | 0.75 | 7.66E-06 | 0.000376968 | Igf1/Egfr/Hgf | 3 |
| 20 | Biological Process | GO:0018108 | peptidyl-tyrosine phosphorylation | 0.75 | 8.62E-06 | 0.000395209 | Igf1/Egfr/Hgf | 3 |
| 21 | Biological Process | GO:0018212 | peptidyl-tyrosine modification | 0.75 | 8.88E-06 | 0.000395209 | Igf1/Egfr/Hgf | 3 |
| 22 | Biological Process | GO:0070372 | regulation of ERK1 and ERK2 cascade | 0.75 | 9.31E-06 | 0.000395612 | Igf1/Ccl11/Egfr | 3 |
| 23 | Biological Process | GO:0070371 | ERK1 and ERK2 cascade | 0.75 | 1.10E-05 | 0.000443199 | Igf1/Ccl11/Egfr | 3 |
| 24 | Biological Process | GO:0050678 | regulation of epithelial cell proliferation | 0.75 | 1.14E-05 | 0.000443199 | Igf1/Ccl11/Egfr | 3 |
| 25 | Biological Process | GO:0007435 | salivary gland morphogenesis | 0.5 | 1.40E-05 | 0.000524775 | Egfr/Hgf | 2 |
| 26 | Biological Process | GO:0007431 | salivary gland development | 0.5 | 1.73E-05 | 0.000621129 | Egfr/Hgf | 2 |
| 27 | Biological Process | GO:0050673 | epithelial cell proliferation | 0.75 | 1.89E-05 | 0.000655625 | Igf1/Ccl11/Egfr | 3 |
| 28 | Biological Process | GO:0051052 | regulation of DNA metabolic process | 0.75 | 2.01E-05 | 0.000658295 | Igf1/Egfr/Hgf | 3 |
| 29 | Biological Process | GO:0090199 | regulation of release of cytochrome c from mitochondria | 0.5 | 2.08E-05 | 0.000658295 | Igf1/Hgf | 2 |
| 30 | Biological Process | GO:0071900 | regulation of protein serine/threonine kinase activity | 0.75 | 2.11E-05 | 0.000658295 | Igf1/Egfr/Hgf | 3 |
| 31 | Biological Process | GO:0045860 | positive regulation of protein kinase activity | 0.75 | 2.40E-05 | 0.000722754 | Igf1/Egfr/Hgf | 3 |
| 32 | Biological Process | GO:0010823 | negative regulation of mitochondrion organization | 0.5 | 2.68E-05 | 0.000784257 | Igf1/Hgf | 2 |
| 33 | Biological Process | GO:1905207 | regulation of cardiocyte differentiation | 0.5 | 2.79E-05 | 0.000790867 | Igf1/Egfr | 2 |
| 34 | Biological Process | GO:0045740 | positive regulation of DNA replication | 0.5 | 3.48E-05 | 0.000956582 | Igf1/Egfr | 2 |
| 35 | Biological Process | GO:0001836 | release of cytochrome c from mitochondria | 0.5 | 3.98E-05 | 0.001062807 | Igf1/Hgf | 2 |
| 36 | Biological Process | GO:0014068 | positive regulation of phosphatidylinositol 3-kinase signaling | 0.5 | 4.51E-05 | 0.001171813 | Igf1/Hgf | 2 |
| 37 | Biological Process | GO:0048146 | positive regulation of fibroblast proliferation | 0.5 | 4.65E-05 | 0.001175158 | Igf1/Egfr | 2 |
| 38 | Biological Process | GO:0043154 | negative regulation of cysteine-type endopeptidase activity involved in apoptotic process | 0.5 | 7.68E-05 | 0.001889517 | Igf1/Hgf | 2 |
| 39 | Biological Process | GO:0051897 | positive regulation of protein kinase B signaling | 0.5 | 8.41E-05 | 0.002016914 | Igf1/Egfr | 2 |
| 40 | Biological Process | GO:0048661 | positive regulation of smooth muscle cell proliferation | 0.5 | 9.58E-05 | 0.002229504 | Igf1/Egfr | 2 |
| 41 | Biological Process | GO:2001237 | negative regulation of extrinsic apoptotic signaling pathway | 0.5 | 9.78E-05 | 0.002229504 | Igf1/Hgf | 2 |
| 42 | Biological Process | GO:0048145 | regulation of fibroblast proliferation | 0.5 | 0.000110263 | 0.002397574 | Igf1/Egfr | 2 |
| 43 | Biological Process | GO:2000117 | negative regulation of cysteine-type endopeptidase activity | 0.5 | 0.000110263 | 0.002397574 | Igf1/Hgf | 2 |
| 44 | Biological Process | GO:0048144 | fibroblast proliferation | 0.5 | 0.000114595 | 0.002435142 | Igf1/Egfr | 2 |
| 45 | Biological Process | GO:0000187 | activation of MAPK activity | 0.5 | 0.000123508 | 0.002566225 | Igf1/Hgf | 2 |
| 46 | Biological Process | GO:0008637 | apoptotic mitochondrial changes | 0.5 | 0.000132753 | 0.002698349 | Igf1/Hgf | 2 |
| 47 | Biological Process | GO:0006275 | regulation of DNA replication | 0.5 | 0.000144775 | 0.002880101 | Igf1/Egfr | 2 |
| 48 | Biological Process | GO:0031099 | regeneration | 0.5 | 0.000162475 | 0.003164883 | Igf1/Hgf | 2 |
| 49 | Biological Process | GO:0055123 | digestive system development | 0.5 | 0.000195174 | 0.003724245 | Igf1/Egfr | 2 |
| 50 | Biological Process | GO:0051896 | regulation of protein kinase B signaling | 0.5 | 0.000203814 | 0.003790132 | Igf1/Egfr | 2 |
| 51 | Biological Process | GO:0034614 | cellular response to reactive oxygen species | 0.5 | 0.000206734 | 0.003790132 | Egfr/Hgf | 2 |
| 52 | Biological Process | GO:0048660 | regulation of smooth muscle cell proliferation | 0.5 | 0.000230844 | 0.004150754 | Igf1/Egfr | 2 |
| 53 | Biological Process | GO:0033135 | regulation of peptidyl-serine phosphorylation | 0.5 | 0.000240225 | 0.004237931 | Egfr/Hgf | 2 |
| 54 | Biological Process | GO:0048659 | smooth muscle cell proliferation | 0.5 | 0.000246582 | 0.004269518 | Igf1/Egfr | 2 |
| 55 | Biological Process | GO:0030879 | mammary gland development | 0.5 | 0.000272832 | 0.004555314 | Igf1/Ccl11 | 2 |
| 56 | Biological Process | GO:2001236 | regulation of extrinsic apoptotic signaling pathway | 0.5 | 0.000272832 | 0.004555314 | Igf1/Hgf | 2 |
| 57 | Biological Process | GO:0035051 | cardiocyte differentiation | 0.5 | 0.0002796 | 0.004586414 | Igf1/Egfr | 2 |
| 58 | Biological Process | GO:0043491 | protein kinase B signaling | 0.5 | 0.000293382 | 0.004729518 | Igf1/Egfr | 2 |
| 59 | Biological Process | GO:0045766 | positive regulation of angiogenesis | 0.5 | 0.000314671 | 0.004986733 | Ccl11/Hgf | 2 |
| 60 | Biological Process | GO:0050731 | positive regulation of peptidyl-tyrosine phosphorylation | 0.5 | 0.000336698 | 0.005083885 | Igf1/Hgf | 2 |
| 61 | Biological Process | GO:0048771 | tissue remodeling | 0.5 | 0.000340441 | 0.005083885 | Igf1/Egfr | 2 |
| 62 | Biological Process | GO:0048754 | branching morphogenesis of an epithelial tube | 0.5 | 0.000344204 | 0.005083885 | Igf1/Ccl11 | 2 |
| 63 | Biological Process | GO:2000377 | regulation of reactive oxygen species metabolic process | 0.5 | 0.000344204 | 0.005083885 | Igf1/Egfr | 2 |
| 64 | Biological Process | GO:0030307 | positive regulation of cell growth | 0.5 | 0.000347988 | 0.005083885 | Igf1/Egfr | 2 |
| 65 | Biological Process | GO:1904018 | positive regulation of vasculature development | 0.5 | 0.000378995 | 0.005451697 | Ccl11/Hgf | 2 |
| 66 | Biological Process | GO:0000302 | response to reactive oxygen species | 0.5 | 0.00039096 | 0.005538599 | Egfr/Hgf | 2 |
| 67 | Biological Process | GO:0010821 | regulation of mitochondrion organization | 0.5 | 0.000399039 | 0.005568676 | Igf1/Hgf | 2 |
| 68 | Biological Process | GO:0010951 | negative regulation of endopeptidase activity | 0.5 | 0.000419593 | 0.005769407 | Igf1/Hgf | 2 |
| 69 | Biological Process | GO:0043281 | regulation of cysteine-type endopeptidase activity involved in apoptotic process | 0.5 | 0.000432171 | 0.005856227 | Igf1/Hgf | 2 |
| 70 | Biological Process | GO:0032872 | regulation of stress-activated MAPK cascade | 0.5 | 0.000453541 | 0.006029781 | Egfr/Hgf | 2 |
| 71 | Biological Process | GO:0070302 | regulation of stress-activated protein kinase signaling cascade | 0.5 | 0.000457876 | 0.006029781 | Egfr/Hgf | 2 |
| 72 | Biological Process | GO:0033002 | muscle cell proliferation | 0.5 | 0.000484316 | 0.006289376 | Igf1/Egfr | 2 |
| 73 | Biological Process | GO:0010001 | glial cell differentiation | 0.5 | 0.00049781 | 0.006347245 | Igf1/Egfr | 2 |
| 74 | Biological Process | GO:2001234 | negative regulation of apoptotic signaling pathway | 0.5 | 0.000502349 | 0.006347245 | Igf1/Hgf | 2 |
| 75 | Biological Process | GO:0097191 | extrinsic apoptotic signaling pathway | 0.5 | 0.000539391 | 0.006724411 | Igf1/Hgf | 2 |
| 76 | Biological Process | GO:2000116 | regulation of cysteine-type endopeptidase activity | 0.5 | 0.000568026 | 0.006988218 | Igf1/Hgf | 2 |
| 77 | Biological Process | GO:0051403 | stress-activated MAPK cascade | 0.5 | 0.000582618 | 0.007074644 | Egfr/Hgf | 2 |
| 78 | Biological Process | GO:0070374 | positive regulation of ERK1 and ERK2 cascade | 0.5 | 0.000607342 | 0.007280322 | Ccl11/Egfr | 2 |
| 79 | Biological Process | GO:0034599 | cellular response to oxidative stress | 0.5 | 0.000642808 | 0.007607916 | Egfr/Hgf | 2 |
| 80 | Biological Process | GO:0010466 | negative regulation of peptidase activity | 0.5 | 0.000653123 | 0.007633376 | Igf1/Hgf | 2 |
| 81 | Biological Process | GO:0072593 | reactive oxygen species metabolic process | 0.5 | 0.000679265 | 0.007840898 | Igf1/Egfr | 2 |
| 82 | Biological Process | GO:0006260 | DNA replication | 0.5 | 0.000700542 | 0.007987892 | Igf1/Egfr | 2 |
| 83 | Biological Process | GO:0048511 | rhythmic process | 0.5 | 0.000749598 | 0.008356504 | Igf1/Egfr | 2 |
| 84 | Biological Process | GO:0031098 | stress-activated protein kinase signaling cascade | 0.5 | 0.000755149 | 0.008356504 | Egfr/Hgf | 2 |
| 85 | Biological Process | GO:0007611 | learning or memory | 0.5 | 0.000766313 | 0.008356504 | Igf1/Egfr | 2 |
| 86 | Biological Process | GO:0050714 | positive regulation of protein secretion | 0.5 | 0.000771925 | 0.008356504 | Igf1/Egfr | 2 |
| 87 | Biological Process | GO:0060326 | cell chemotaxis | 0.5 | 0.000777557 | 0.008356504 | Ccl11/Hgf | 2 |
| 88 | Biological Process | GO:0014834 | skeletal muscle satellite cell maintenance involved in skeletal muscle regeneration | 0.25 | 0.00083861 | 0.008810111 | Igf1 | 1 |
| 89 | Biological Process | GO:0060665 | regulation of branching involved in salivary gland morphogenesis by mesenchymal-epithelial signaling | 0.25 | 0.00083861 | 0.008810111 | Hgf | 1 |
| 90 | Biological Process | GO:0045765 | regulation of angiogenesis | 0.5 | 0.000870409 | 0.009028771 | Ccl11/Hgf | 2 |
| 91 | Biological Process | GO:0042063 | gliogenesis | 0.5 | 0.000888392 | 0.009028771 | Igf1/Egfr | 2 |
| 92 | Biological Process | GO:0045927 | positive regulation of growth | 0.5 | 0.000888392 | 0.009028771 | Igf1/Egfr | 2 |
| 93 | Biological Process | GO:0046677 | response to antibiotic | 0.5 | 0.000931055 | 0.009346463 | Igf1/Hgf | 2 |
| 94 | Biological Process | GO:0002793 | positive regulation of peptide secretion | 0.5 | 0.000943426 | 0.009346463 | Igf1/Egfr | 2 |
| 95 | Biological Process | GO:0050890 | cognition | 0.5 | 0.000949641 | 0.009346463 | Igf1/Egfr | 2 |
| 96 | Biological Process | GO:0010749 | regulation of nitric oxide mediated signal transduction | 0.25 | 0.001006268 | 0.009462084 | Egfr | 1 |
| 97 | Biological Process | GO:1902947 | regulation of tau-protein kinase activity | 0.25 | 0.001006268 | 0.009462084 | Hgf | 1 |
| 98 | Biological Process | GO:1905288 | vascular associated smooth muscle cell apoptotic process | 0.25 | 0.001006268 | 0.009462084 | Igf1 | 1 |
| 99 | Biological Process | GO:1905459 | regulation of vascular associated smooth muscle cell apoptotic process | 0.25 | 0.001006268 | 0.009462084 | Igf1 | 1 |
| 100 | Biological Process | GO:0050727 | regulation of inflammatory response | 0.5 | 0.001019329 | 0.009462084 | Egfr/Hgf | 2 |
| 101 | Biological Process | GO:1901342 | regulation of vasculature development | 0.5 | 0.00103226 | 0.009462084 | Ccl11/Hgf | 2 |
| 102 | Biological Process | GO:0018105 | peptidyl-serine phosphorylation | 0.5 | 0.001091439 | 0.009462084 | Egfr/Hgf | 2 |
| 103 | Biological Process | GO:0019932 | second-messenger-mediated signaling | 0.5 | 0.001138586 | 0.009462084 | Igf1/Egfr | 2 |
| 104 | Biological Process | GO:0009636 | response to toxic substance | 0.5 | 0.001172861 | 0.009462084 | Igf1/Hgf | 2 |
| 105 | Biological Process | GO:0042060 | wound healing | 0.5 | 0.001172861 | 0.009462084 | Igf1/Egfr | 2 |
| 106 | Biological Process | GO:0060283 | negative regulation of oocyte development | 0.25 | 0.001173906 | 0.009462084 | Igf1 | 1 |
| 107 | Biological Process | GO:0060509 | Type I pneumocyte differentiation | 0.25 | 0.001173906 | 0.009462084 | Igf1 | 1 |
| 108 | Biological Process | GO:0060510 | Type II pneumocyte differentiation | 0.25 | 0.001173906 | 0.009462084 | Igf1 | 1 |
| 109 | Biological Process | GO:0060526 | prostate glandular acinus morphogenesis | 0.25 | 0.001173906 | 0.009462084 | Igf1 | 1 |
| 110 | Biological Process | GO:0060527 | prostate epithelial cord arborization involved in prostate glandular acinus morphogenesis | 0.25 | 0.001173906 | 0.009462084 | Igf1 | 1 |
| 111 | Biological Process | GO:0060763 | mammary duct terminal end bud growth | 0.25 | 0.001173906 | 0.009462084 | Ccl11 | 1 |
| 112 | Biological Process | GO:0070141 | response to UV-A | 0.25 | 0.001173906 | 0.009462084 | Egfr | 1 |
| 113 | Biological Process | GO:0070459 | prolactin secretion | 0.25 | 0.001173906 | 0.009462084 | Egfr | 1 |
| 114 | Biological Process | GO:1900019 | regulation of protein kinase C activity | 0.25 | 0.001173906 | 0.009462084 | Egfr | 1 |
| 115 | Biological Process | GO:1900020 | positive regulation of protein kinase C activity | 0.25 | 0.001173906 | 0.009462084 | Egfr | 1 |
| 116 | Biological Process | GO:1905880 | negative regulation of oogenesis | 0.25 | 0.001173906 | 0.009462084 | Igf1 | 1 |
| 117 | Biological Process | GO:1903829 | positive regulation of cellular protein localization | 0.5 | 0.001193665 | 0.00953912 | Igf1/Egfr | 2 |
| 118 | Biological Process | GO:0045787 | positive regulation of cell cycle | 0.5 | 0.001214649 | 0.009624547 | Igf1/Egfr | 2 |
| 119 | Biological Process | GO:0045861 | negative regulation of proteolysis | 0.5 | 0.001235811 | 0.009709947 | Igf1/Hgf | 2 |
| 120 | Biological Process | GO:0052548 | regulation of endopeptidase activity | 0.5 | 0.001257153 | 0.009795318 | Igf1/Hgf | 2 |
| 121 | Biological Process | GO:0018209 | peptidyl-serine modification | 0.5 | 0.001336937 | 0.010034585 | Egfr/Hgf | 2 |
| 122 | Biological Process | GO:0031659 | positive regulation of cyclin-dependent protein serine/threonine kinase activity involved in G1/S transition of mitotic cell cycle | 0.25 | 0.001341522 | 0.010034585 | Egfr | 1 |
| 123 | Biological Process | GO:1901299 | negative regulation of hydrogen peroxide-mediated programmed cell death | 0.25 | 0.001341522 | 0.010034585 | Hgf | 1 |
| 124 | Biological Process | GO:1903800 | positive regulation of production of miRNAs involved in gene silencing by miRNA | 0.25 | 0.001341522 | 0.010034585 | Egfr | 1 |
| 125 | Biological Process | GO:1990314 | cellular response to insulin-like growth factor stimulus | 0.25 | 0.001341522 | 0.010034585 | Igf1 | 1 |
| 126 | Biological Process | GO:0010639 | negative regulation of organelle organization | 0.5 | 0.001404002 | 0.010418585 | Igf1/Hgf | 2 |
| 127 | Biological Process | GO:0060562 | epithelial tube morphogenesis | 0.5 | 0.001419123 | 0.010447875 | Igf1/Ccl11 | 2 |
| 128 | Biological Process | GO:0031657 | regulation of cyclin-dependent protein serine/threonine kinase activity involved in G1/S transition of mitotic cell cycle | 0.25 | 0.001509117 | 0.010452035 | Egfr | 1 |
| 129 | Biological Process | GO:0034351 | negative regulation of glial cell apoptotic process | 0.25 | 0.001509117 | 0.010452035 | Igf1 | 1 |
| 130 | Biological Process | GO:0034392 | negative regulation of smooth muscle cell apoptotic process | 0.25 | 0.001509117 | 0.010452035 | Igf1 | 1 |
| 131 | Biological Process | GO:0060426 | lung vasculature development | 0.25 | 0.001509117 | 0.010452035 | Igf1 | 1 |
| 132 | Biological Process | GO:0060462 | lung lobe development | 0.25 | 0.001509117 | 0.010452035 | Igf1 | 1 |
| 133 | Biological Process | GO:0060463 | lung lobe morphogenesis | 0.25 | 0.001509117 | 0.010452035 | Igf1 | 1 |
| 134 | Biological Process | GO:0060638 | mesenchymal-epithelial cell signaling | 0.25 | 0.001509117 | 0.010452035 | Hgf | 1 |
| 135 | Biological Process | GO:0090031 | positive regulation of steroid hormone biosynthetic process | 0.25 | 0.001509117 | 0.010452035 | Igf1 | 1 |
| 136 | Biological Process | GO:0006979 | response to oxidative stress | 0.5 | 0.001527191 | 0.010499435 | Egfr/Hgf | 2 |
| 137 | Biological Process | GO:2001233 | regulation of apoptotic signaling pathway | 0.5 | 0.001614819 | 0.010995759 | Igf1/Hgf | 2 |
| 138 | Biological Process | GO:0051346 | negative regulation of hydrolase activity | 0.5 | 0.001622903 | 0.010995759 | Igf1/Hgf | 2 |
| 139 | Biological Process | GO:0006417 | regulation of translation | 0.5 | 0.001655439 | 0.011040187 | Igf1/Egfr | 2 |
| 140 | Biological Process | GO:0021940 | positive regulation of cerebellar granule cell precursor proliferation | 0.25 | 0.001676692 | 0.011040187 | Igf1 | 1 |
| 141 | Biological Process | GO:0031017 | exocrine pancreas development | 0.25 | 0.001676692 | 0.011040187 | Igf1 | 1 |
| 142 | Biological Process | GO:0034350 | regulation of glial cell apoptotic process | 0.25 | 0.001676692 | 0.011040187 | Igf1 | 1 |
| 143 | Biological Process | GO:0052547 | regulation of peptidase activity | 0.5 | 0.001704834 | 0.011146993 | Igf1/Hgf | 2 |
| 144 | Biological Process | GO:0001933 | negative regulation of protein phosphorylation | 0.5 | 0.001754938 | 0.01139491 | Igf1/Hgf | 2 |
| 145 | Biological Process | GO:0001558 | regulation of cell growth | 0.5 | 0.001771797 | 0.011425034 | Igf1/Egfr | 2 |
| 146 | Biological Process | GO:0035630 | bone mineralization involved in bone maturation | 0.25 | 0.001844245 | 0.011572944 | Igf1 | 1 |
| 147 | Biological Process | GO:0043568 | positive regulation of insulin-like growth factor receptor signaling pathway | 0.25 | 0.001844245 | 0.011572944 | Igf1 | 1 |
| 148 | Biological Process | GO:0060693 | regulation of branching involved in salivary gland morphogenesis | 0.25 | 0.001844245 | 0.011572944 | Hgf | 1 |
| 149 | Biological Process | GO:2000288 | positive regulation of myoblast proliferation | 0.25 | 0.001844245 | 0.011572944 | Igf1 | 1 |
| 150 | Biological Process | GO:0010035 | response to inorganic substance | 0.5 | 0.001980227 | 0.011907967 | Egfr/Hgf | 2 |
| 151 | Biological Process | GO:1903532 | positive regulation of secretion by cell | 0.5 | 0.001980227 | 0.011907967 | Igf1/Egfr | 2 |
| 152 | Biological Process | GO:0034248 | regulation of cellular amide metabolic process | 0.5 | 0.001998106 | 0.011907967 | Igf1/Egfr | 2 |
| 153 | Biological Process | GO:0048143 | astrocyte activation | 0.25 | 0.002011777 | 0.011907967 | Egfr | 1 |
| 154 | Biological Process | GO:0060525 | prostate glandular acinus development | 0.25 | 0.002011777 | 0.011907967 | Igf1 | 1 |
| 155 | Biological Process | GO:0061051 | positive regulation of cell growth involved in cardiac muscle cell development | 0.25 | 0.002011777 | 0.011907967 | Igf1 | 1 |
| 156 | Biological Process | GO:0070572 | positive regulation of neuron projection regeneration | 0.25 | 0.002011777 | 0.011907967 | Hgf | 1 |
| 157 | Biological Process | GO:1901298 | regulation of hydrogen peroxide-mediated programmed cell death | 0.25 | 0.002011777 | 0.011907967 | Hgf | 1 |
| 158 | Biological Process | GO:0048608 | reproductive structure development | 0.5 | 0.002016063 | 0.011907967 | Igf1/Egfr | 2 |
| 159 | Biological Process | GO:0050708 | regulation of protein secretion | 0.5 | 0.002025071 | 0.011907967 | Igf1/Egfr | 2 |
| 160 | Biological Process | GO:0061458 | reproductive system development | 0.5 | 0.002052213 | 0.011907967 | Igf1/Egfr | 2 |
| 161 | Biological Process | GO:0001701 | in utero embryonic development | 0.5 | 0.00207953 | 0.011907967 | Igf1/Egfr | 2 |
| 162 | Biological Process | GO:0042326 | negative regulation of phosphorylation | 0.5 | 0.002125449 | 0.011907967 | Igf1/Hgf | 2 |
| 163 | Biological Process | GO:0002551 | mast cell chemotaxis | 0.25 | 0.002179288 | 0.011907967 | Ccl11 | 1 |
| 164 | Biological Process | GO:0021924 | cell proliferation in external granule layer | 0.25 | 0.002179288 | 0.011907967 | Igf1 | 1 |
| 165 | Biological Process | GO:0021930 | cerebellar granule cell precursor proliferation | 0.25 | 0.002179288 | 0.011907967 | Igf1 | 1 |
| 166 | Biological Process | GO:0021936 | regulation of cerebellar granule cell precursor proliferation | 0.25 | 0.002179288 | 0.011907967 | Igf1 | 1 |
| 167 | Biological Process | GO:0048012 | hepatocyte growth factor receptor signaling pathway | 0.25 | 0.002179288 | 0.011907967 | Hgf | 1 |
| 168 | Biological Process | GO:0060281 | regulation of oocyte development | 0.25 | 0.002179288 | 0.011907967 | Igf1 | 1 |
| 169 | Biological Process | GO:0060766 | negative regulation of androgen receptor signaling pathway | 0.25 | 0.002179288 | 0.011907967 | Igf1 | 1 |
| 170 | Biological Process | GO:0061140 | lung secretory cell differentiation | 0.25 | 0.002179288 | 0.011907967 | Igf1 | 1 |
| 171 | Biological Process | GO:1905879 | regulation of oogenesis | 0.25 | 0.002179288 | 0.011907967 | Igf1 | 1 |
| 172 | Biological Process | GO:0009611 | response to wounding | 0.5 | 0.002190557 | 0.011907967 | Igf1/Egfr | 2 |
| 173 | Biological Process | GO:0051222 | positive regulation of protein transport | 0.5 | 0.002209334 | 0.0119252 | Igf1/Egfr | 2 |
| 174 | Biological Process | GO:0010608 | posttranscriptional regulation of gene expression | 0.5 | 0.00227567 | 0.0119252 | Igf1/Egfr | 2 |
| 175 | Biological Process | GO:1904951 | positive regulation of establishment of protein localization | 0.5 | 0.002323636 | 0.0119252 | Igf1/Egfr | 2 |
| 176 | Biological Process | GO:0010421 | hydrogen peroxide-mediated programmed cell death | 0.25 | 0.002346777 | 0.0119252 | Hgf | 1 |
| 177 | Biological Process | GO:0021534 | cell proliferation in hindbrain | 0.25 | 0.002346777 | 0.0119252 | Igf1 | 1 |
| 178 | Biological Process | GO:0034349 | glial cell apoptotic process | 0.25 | 0.002346777 | 0.0119252 | Igf1 | 1 |
| 179 | Biological Process | GO:0035729 | cellular response to hepatocyte growth factor stimulus | 0.25 | 0.002346777 | 0.0119252 | Hgf | 1 |
| 180 | Biological Process | GO:0060736 | prostate gland growth | 0.25 | 0.002346777 | 0.0119252 | Igf1 | 1 |
| 181 | Biological Process | GO:0070886 | positive regulation of calcineurin-NFAT signaling cascade | 0.25 | 0.002346777 | 0.0119252 | Igf1 | 1 |
| 182 | Biological Process | GO:0097468 | programmed cell death in response to reactive oxygen species | 0.25 | 0.002346777 | 0.0119252 | Hgf | 1 |
| 183 | Biological Process | GO:0097531 | mast cell migration | 0.25 | 0.002346777 | 0.0119252 | Ccl11 | 1 |
| 184 | Biological Process | GO:0106058 | positive regulation of calcineurin-mediated signaling | 0.25 | 0.002346777 | 0.0119252 | Igf1 | 1 |
| 185 | Biological Process | GO:0002791 | regulation of peptide secretion | 0.5 | 0.002391607 | 0.012087309 | Igf1/Egfr | 2 |
| 186 | Biological Process | GO:0001525 | angiogenesis | 0.5 | 0.002480396 | 0.012180415 | Ccl11/Hgf | 2 |
| 187 | Biological Process | GO:0051047 | positive regulation of secretion | 0.5 | 0.002480396 | 0.012180415 | Igf1/Egfr | 2 |
| 188 | Biological Process | GO:0010960 | magnesium ion homeostasis | 0.25 | 0.002514246 | 0.012180415 | Egfr | 1 |
| 189 | Biological Process | GO:0045821 | positive regulation of glycolytic process | 0.25 | 0.002514246 | 0.012180415 | Igf1 | 1 |
| 190 | Biological Process | GO:0046628 | positive regulation of insulin receptor signaling pathway | 0.25 | 0.002514246 | 0.012180415 | Igf1 | 1 |
| 191 | Biological Process | GO:0060252 | positive regulation of glial cell proliferation | 0.25 | 0.002514246 | 0.012180415 | Igf1 | 1 |
| 192 | Biological Process | GO:0060442 | branching involved in prostate gland morphogenesis | 0.25 | 0.002514246 | 0.012180415 | Igf1 | 1 |
| 193 | Biological Process | GO:0061029 | eyelid development in camera-type eye | 0.25 | 0.002514246 | 0.012180415 | Egfr | 1 |
| 194 | Biological Process | GO:0016049 | cell growth | 0.5 | 0.00255054 | 0.012292551 | Igf1/Egfr | 2 |
| 195 | Biological Process | GO:0035728 | response to hepatocyte growth factor | 0.25 | 0.002681694 | 0.012599918 | Hgf | 1 |
| 196 | Biological Process | GO:0045725 | positive regulation of glycogen biosynthetic process | 0.25 | 0.002681694 | 0.012599918 | Igf1 | 1 |
| 197 | Biological Process | GO:0051197 | positive regulation of coenzyme metabolic process | 0.25 | 0.002681694 | 0.012599918 | Igf1 | 1 |
| 198 | Biological Process | GO:1903798 | regulation of production of miRNAs involved in gene silencing by miRNA | 0.25 | 0.002681694 | 0.012599918 | Egfr | 1 |
| 199 | Biological Process | GO:2000291 | regulation of myoblast proliferation | 0.25 | 0.002681694 | 0.012599918 | Igf1 | 1 |
| 200 | Biological Process | GO:0030813 | positive regulation of nucleotide catabolic process | 0.25 | 0.00284912 | 0.013058469 | Igf1 | 1 |
| 201 | Biological Process | GO:0031643 | positive regulation of myelination | 0.25 | 0.00284912 | 0.013058469 | Hgf | 1 |
| 202 | Biological Process | GO:0032930 | positive regulation of superoxide anion generation | 0.25 | 0.00284912 | 0.013058469 | Egfr | 1 |
| 203 | Biological Process | GO:0050650 | chondroitin sulfate proteoglycan biosynthetic process | 0.25 | 0.00284912 | 0.013058469 | Igf1 | 1 |
| 204 | Biological Process | GO:0070920 | regulation of production of small RNA involved in gene silencing by RNA | 0.25 | 0.00284912 | 0.013058469 | Egfr | 1 |
| 205 | Biological Process | GO:0033160 | positive regulation of protein import into nucleus, translocation | 0.25 | 0.003016526 | 0.013559864 | Igf1 | 1 |
| 206 | Biological Process | GO:0043931 | ossification involved in bone maturation | 0.25 | 0.003016526 | 0.013559864 | Igf1 | 1 |
| 207 | Biological Process | GO:0070875 | positive regulation of glycogen metabolic process | 0.25 | 0.003016526 | 0.013559864 | Igf1 | 1 |
| 208 | Biological Process | GO:0090030 | regulation of steroid hormone biosynthetic process | 0.25 | 0.003016526 | 0.013559864 | Igf1 | 1 |
| 209 | Biological Process | GO:0046886 | positive regulation of hormone biosynthetic process | 0.25 | 0.00318391 | 0.014175982 | Igf1 | 1 |
| 210 | Biological Process | GO:0070977 | bone maturation | 0.25 | 0.00318391 | 0.014175982 | Igf1 | 1 |
| 211 | Biological Process | GO:0010560 | positive regulation of glycoprotein biosynthetic process | 0.25 | 0.003351274 | 0.014642247 | Igf1 | 1 |
| 212 | Biological Process | GO:0032148 | activation of protein kinase B activity | 0.25 | 0.003351274 | 0.014642247 | Igf1 | 1 |
| 213 | Biological Process | GO:0051194 | positive regulation of cofactor metabolic process | 0.25 | 0.003351274 | 0.014642247 | Igf1 | 1 |
| 214 | Biological Process | GO:1905208 | negative regulation of cardiocyte differentiation | 0.25 | 0.003351274 | 0.014642247 | Egfr | 1 |
| 215 | Biological Process | GO:0032928 | regulation of superoxide anion generation | 0.25 | 0.003518616 | 0.015160857 | Egfr | 1 |
| 216 | Biological Process | GO:0048245 | eosinophil chemotaxis | 0.25 | 0.003518616 | 0.015160857 | Ccl11 | 1 |
| 217 | Biological Process | GO:0061050 | regulation of cell growth involved in cardiac muscle cell development | 0.25 | 0.003518616 | 0.015160857 | Igf1 | 1 |
| 218 | Biological Process | GO:0007263 | nitric oxide mediated signal transduction | 0.25 | 0.003685937 | 0.015665233 | Egfr | 1 |
| 219 | Biological Process | GO:1903020 | positive regulation of glycoprotein metabolic process | 0.25 | 0.003685937 | 0.015665233 | Igf1 | 1 |
| 220 | Biological Process | GO:2000637 | positive regulation of gene silencing by miRNA | 0.25 | 0.003685937 | 0.015665233 | Egfr | 1 |
| 221 | Biological Process | GO:0032352 | positive regulation of hormone metabolic process | 0.25 | 0.003853237 | 0.015801653 | Igf1 | 1 |
| 222 | Biological Process | GO:0034390 | smooth muscle cell apoptotic process | 0.25 | 0.003853237 | 0.015801653 | Igf1 | 1 |
| 223 | Biological Process | GO:0034391 | regulation of smooth muscle cell apoptotic process | 0.25 | 0.003853237 | 0.015801653 | Igf1 | 1 |
| 224 | Biological Process | GO:0048799 | animal organ maturation | 0.25 | 0.003853237 | 0.015801653 | Igf1 | 1 |
| 225 | Biological Process | GO:0060148 | positive regulation of posttranscriptional gene silencing | 0.25 | 0.003853237 | 0.015801653 | Egfr | 1 |
| 226 | Biological Process | GO:0071276 | cellular response to cadmium ion | 0.25 | 0.003853237 | 0.015801653 | Egfr | 1 |
| 227 | Biological Process | GO:1904707 | positive regulation of vascular smooth muscle cell proliferation | 0.25 | 0.003853237 | 0.015801653 | Igf1 | 1 |
| 228 | Biological Process | GO:2000679 | positive regulation of transcription regulatory region DNA binding | 0.25 | 0.003853237 | 0.015801653 | Igf1 | 1 |
| 229 | Biological Process | GO:0043567 | regulation of insulin-like growth factor receptor signaling pathway | 0.25 | 0.004020516 | 0.016133832 | Igf1 | 1 |
| 230 | Biological Process | GO:0071392 | cellular response to estradiol stimulus | 0.25 | 0.004020516 | 0.016133832 | Egfr | 1 |
| 231 | Biological Process | GO:1904646 | cellular response to amyloid-beta | 0.25 | 0.004020516 | 0.016133832 | Igf1 | 1 |
| 232 | Biological Process | GO:2000727 | positive regulation of cardiac muscle cell differentiation | 0.25 | 0.004020516 | 0.016133832 | Igf1 | 1 |
| 233 | Biological Process | GO:2001171 | positive regulation of ATP biosynthetic process | 0.25 | 0.004020516 | 0.016133832 | Igf1 | 1 |
| 234 | Biological Process | GO:0010893 | positive regulation of steroid biosynthetic process | 0.25 | 0.004187774 | 0.016247174 | Igf1 | 1 |
| 235 | Biological Process | GO:0032878 | regulation of establishment or maintenance of cell polarity | 0.25 | 0.004187774 | 0.016247174 | Igf1 | 1 |
| 236 | Biological Process | GO:0042104 | positive regulation of activated T cell proliferation | 0.25 | 0.004187774 | 0.016247174 | Igf1 | 1 |
| 237 | Biological Process | GO:0060765 | regulation of androgen receptor signaling pathway | 0.25 | 0.004187774 | 0.016247174 | Igf1 | 1 |
| 238 | Biological Process | GO:0072677 | eosinophil migration | 0.25 | 0.004187774 | 0.016247174 | Ccl11 | 1 |
| 239 | Biological Process | GO:1901032 | negative regulation of response to reactive oxygen species | 0.25 | 0.004187774 | 0.016247174 | Hgf | 1 |
| 240 | Biological Process | GO:1903206 | negative regulation of hydrogen peroxide-induced cell death | 0.25 | 0.004187774 | 0.016247174 | Hgf | 1 |
| 241 | Biological Process | GO:2001039 | negative regulation of cellular response to drug | 0.25 | 0.004187774 | 0.016247174 | Hgf | 1 |
| 242 | Biological Process | GO:0045780 | positive regulation of bone resorption | 0.25 | 0.004355011 | 0.01668826 | Egfr | 1 |
| 243 | Biological Process | GO:0046852 | positive regulation of bone remodeling | 0.25 | 0.004355011 | 0.01668826 | Egfr | 1 |
| 244 | Biological Process | GO:1904645 | response to amyloid-beta | 0.25 | 0.004355011 | 0.01668826 | Igf1 | 1 |
| 245 | Biological Process | GO:0033137 | negative regulation of peptidyl-serine phosphorylation | 0.25 | 0.004522227 | 0.017258295 | Hgf | 1 |
| 246 | Biological Process | GO:0001832 | blastocyst growth | 0.25 | 0.004689422 | 0.017330472 | Igf1 | 1 |
| 247 | Biological Process | GO:0005979 | regulation of glycogen biosynthetic process | 0.25 | 0.004689422 | 0.017330472 | Igf1 | 1 |
| 248 | Biological Process | GO:0010962 | regulation of glucan biosynthetic process | 0.25 | 0.004689422 | 0.017330472 | Igf1 | 1 |
| 249 | Biological Process | GO:0033158 | regulation of protein import into nucleus, translocation | 0.25 | 0.004689422 | 0.017330472 | Igf1 | 1 |
| 250 | Biological Process | GO:0050654 | chondroitin sulfate proteoglycan metabolic process | 0.25 | 0.004689422 | 0.017330472 | Igf1 | 1 |
| 251 | Biological Process | GO:0060444 | branching involved in mammary gland duct morphogenesis | 0.25 | 0.004689422 | 0.017330472 | Ccl11 | 1 |
| 252 | Biological Process | GO:0060445 | branching involved in salivary gland morphogenesis | 0.25 | 0.004689422 | 0.017330472 | Hgf | 1 |
| 253 | Biological Process | GO:1902042 | negative regulation of extrinsic apoptotic signaling pathway via death domain receptors | 0.25 | 0.004689422 | 0.017330472 | Hgf | 1 |
| 254 | Biological Process | GO:0060571 | morphogenesis of an epithelial fold | 0.25 | 0.004856596 | 0.017877626 | Egfr | 1 |
| 255 | Biological Process | GO:0014002 | astrocyte development | 0.25 | 0.005023748 | 0.018277061 | Egfr | 1 |
| 256 | Biological Process | GO:0042554 | superoxide anion generation | 0.25 | 0.005023748 | 0.018277061 | Egfr | 1 |
| 257 | Biological Process | GO:1900078 | positive regulation of cellular response to insulin stimulus | 0.25 | 0.005023748 | 0.018277061 | Igf1 | 1 |
| 258 | Biological Process | GO:0050999 | regulation of nitric-oxide synthase activity | 0.25 | 0.00519088 | 0.018595681 | Egfr | 1 |
| 259 | Biological Process | GO:0070884 | regulation of calcineurin-NFAT signaling cascade | 0.25 | 0.00519088 | 0.018595681 | Igf1 | 1 |
| 260 | Biological Process | GO:0090322 | regulation of superoxide metabolic process | 0.25 | 0.00519088 | 0.018595681 | Egfr | 1 |
| 261 | Biological Process | GO:0106056 | regulation of calcineurin-mediated signaling | 0.25 | 0.00519088 | 0.018595681 | Igf1 | 1 |
| 262 | Biological Process | GO:0003298 | physiological muscle hypertrophy | 0.25 | 0.00535799 | 0.018692989 | Igf1 | 1 |
| 263 | Biological Process | GO:0003301 | physiological cardiac muscle hypertrophy | 0.25 | 0.00535799 | 0.018692989 | Igf1 | 1 |
| 264 | Biological Process | GO:0032733 | positive regulation of interleukin-10 production | 0.25 | 0.00535799 | 0.018692989 | Hgf | 1 |
| 265 | Biological Process | GO:0060251 | regulation of glial cell proliferation | 0.25 | 0.00535799 | 0.018692989 | Igf1 | 1 |
| 266 | Biological Process | GO:0060487 | lung epithelial cell differentiation | 0.25 | 0.00535799 | 0.018692989 | Igf1 | 1 |
| 267 | Biological Process | GO:0061049 | cell growth involved in cardiac muscle cell development | 0.25 | 0.00535799 | 0.018692989 | Igf1 | 1 |
| 268 | Biological Process | GO:1905209 | positive regulation of cardiocyte differentiation | 0.25 | 0.00535799 | 0.018692989 | Igf1 | 1 |
| 269 | Biological Process | GO:0043403 | skeletal muscle tissue regeneration | 0.25 | 0.00552508 | 0.018785272 | Igf1 | 1 |
| 270 | Biological Process | GO:0045940 | positive regulation of steroid metabolic process | 0.25 | 0.00552508 | 0.018785272 | Igf1 | 1 |
| 271 | Biological Process | GO:0060479 | lung cell differentiation | 0.25 | 0.00552508 | 0.018785272 | Igf1 | 1 |
| 272 | Biological Process | GO:0060740 | prostate gland epithelium morphogenesis | 0.25 | 0.00552508 | 0.018785272 | Igf1 | 1 |
| 273 | Biological Process | GO:1900744 | regulation of p38MAPK cascade | 0.25 | 0.00552508 | 0.018785272 | Hgf | 1 |
| 274 | Biological Process | GO:1903205 | regulation of hydrogen peroxide-induced cell death | 0.25 | 0.00552508 | 0.018785272 | Hgf | 1 |
| 275 | Biological Process | GO:1903580 | positive regulation of ATP metabolic process | 0.25 | 0.00552508 | 0.018785272 | Igf1 | 1 |
| 276 | Biological Process | GO:0010613 | positive regulation of cardiac muscle hypertrophy | 0.25 | 0.005692148 | 0.018872903 | Igf1 | 1 |
| 277 | Biological Process | GO:0014742 | positive regulation of muscle hypertrophy | 0.25 | 0.005692148 | 0.018872903 | Igf1 | 1 |
| 278 | Biological Process | GO:0031646 | positive regulation of neurological system process | 0.25 | 0.005692148 | 0.018872903 | Hgf | 1 |
| 279 | Biological Process | GO:0033144 | negative regulation of intracellular steroid hormone receptor signaling pathway | 0.25 | 0.005692148 | 0.018872903 | Igf1 | 1 |
| 280 | Biological Process | GO:0060512 | prostate gland morphogenesis | 0.25 | 0.005692148 | 0.018872903 | Igf1 | 1 |
| 281 | Biological Process | GO:0070570 | regulation of neuron projection regeneration | 0.25 | 0.005692148 | 0.018872903 | Hgf | 1 |
| 282 | Biological Process | GO:0070873 | regulation of glycogen metabolic process | 0.25 | 0.005692148 | 0.018872903 | Igf1 | 1 |
| 283 | Biological Process | GO:0036474 | cell death in response to hydrogen peroxide | 0.25 | 0.005859196 | 0.019022042 | Hgf | 1 |
| 284 | Biological Process | GO:0046686 | response to cadmium ion | 0.25 | 0.005859196 | 0.019022042 | Egfr | 1 |
| 285 | Biological Process | GO:0048009 | insulin-like growth factor receptor signaling pathway | 0.25 | 0.005859196 | 0.019022042 | Igf1 | 1 |
| 286 | Biological Process | GO:0051968 | positive regulation of synaptic transmission, glutamatergic | 0.25 | 0.005859196 | 0.019022042 | Egfr | 1 |
| 287 | Biological Process | GO:0071364 | cellular response to epidermal growth factor stimulus | 0.25 | 0.005859196 | 0.019022042 | Egfr | 1 |
| 288 | Biological Process | GO:2001038 | regulation of cellular response to drug | 0.25 | 0.005859196 | 0.019022042 | Hgf | 1 |
| 289 | Biological Process | GO:0006110 | regulation of glycolytic process | 0.25 | 0.006026222 | 0.01910006 | Igf1 | 1 |
| 290 | Biological Process | GO:0032885 | regulation of polysaccharide biosynthetic process | 0.25 | 0.006026222 | 0.01910006 | Igf1 | 1 |
| 291 | Biological Process | GO:0034105 | positive regulation of tissue remodeling | 0.25 | 0.006026222 | 0.01910006 | Egfr | 1 |
| 292 | Biological Process | GO:0040019 | positive regulation of embryonic development | 0.25 | 0.006026222 | 0.01910006 | Igf1 | 1 |
| 293 | Biological Process | GO:0061900 | glial cell activation | 0.25 | 0.006026222 | 0.01910006 | Egfr | 1 |
| 294 | Biological Process | GO:1904705 | regulation of vascular smooth muscle cell proliferation | 0.25 | 0.006026222 | 0.01910006 | Igf1 | 1 |
| 295 | Biological Process | GO:1990874 | vascular smooth muscle cell proliferation | 0.25 | 0.006026222 | 0.01910006 | Igf1 | 1 |
| 296 | Biological Process | GO:0045737 | positive regulation of cyclin-dependent protein serine/threonine kinase activity | 0.25 | 0.006193227 | 0.019366781 | Egfr | 1 |
| 297 | Biological Process | GO:0046006 | regulation of activated T cell proliferation | 0.25 | 0.006193227 | 0.019366781 | Igf1 | 1 |
| 298 | Biological Process | GO:0055023 | positive regulation of cardiac muscle tissue growth | 0.25 | 0.006193227 | 0.019366781 | Igf1 | 1 |
| 299 | Biological Process | GO:2001024 | negative regulation of response to drug | 0.25 | 0.006193227 | 0.019366781 | Hgf | 1 |
| 300 | Biological Process | GO:0030811 | regulation of nucleotide catabolic process | 0.25 | 0.006360212 | 0.019691384 | Igf1 | 1 |
| 301 | Biological Process | GO:0046885 | regulation of hormone biosynthetic process | 0.25 | 0.006360212 | 0.019691384 | Igf1 | 1 |
| 302 | Biological Process | GO:0070849 | response to epidermal growth factor | 0.25 | 0.006360212 | 0.019691384 | Egfr | 1 |
| 303 | Biological Process | GO:0033173 | calcineurin-NFAT signaling cascade | 0.25 | 0.006527175 | 0.020075357 | Igf1 | 1 |
| 304 | Biological Process | GO:0060964 | regulation of gene silencing by miRNA | 0.25 | 0.006527175 | 0.020075357 | Egfr | 1 |
| 305 | Biological Process | GO:0010559 | regulation of glycoprotein biosynthetic process | 0.25 | 0.006694117 | 0.020190321 | Igf1 | 1 |
| 306 | Biological Process | GO:0038066 | p38MAPK cascade | 0.25 | 0.006694117 | 0.020190321 | Hgf | 1 |
| 307 | Biological Process | GO:0060421 | positive regulation of heart growth | 0.25 | 0.006694117 | 0.020190321 | Igf1 | 1 |
| 308 | Biological Process | GO:0060603 | mammary gland duct morphogenesis | 0.25 | 0.006694117 | 0.020190321 | Ccl11 | 1 |
| 309 | Biological Process | GO:1904031 | positive regulation of cyclin-dependent protein kinase activity | 0.25 | 0.006694117 | 0.020190321 | Egfr | 1 |
| 310 | Biological Process | GO:2000725 | regulation of cardiac muscle cell differentiation | 0.25 | 0.006694117 | 0.020190321 | Igf1 | 1 |
| 311 | Biological Process | GO:0000186 | activation of MAPKK activity | 0.25 | 0.006861038 | 0.020218838 | Egfr | 1 |
| 312 | Biological Process | GO:0010907 | positive regulation of glucose metabolic process | 0.25 | 0.006861038 | 0.020218838 | Igf1 | 1 |
| 313 | Biological Process | GO:0014009 | glial cell proliferation | 0.25 | 0.006861038 | 0.020218838 | Igf1 | 1 |
| 314 | Biological Process | GO:0035196 | production of miRNAs involved in gene silencing by miRNA | 0.25 | 0.006861038 | 0.020218838 | Egfr | 1 |
| 315 | Biological Process | GO:0050974 | detection of mechanical stimulus involved in sensory perception | 0.25 | 0.006861038 | 0.020218838 | Igf1 | 1 |
| 316 | Biological Process | GO:0005978 | glycogen biosynthetic process | 0.25 | 0.007027938 | 0.020218838 | Igf1 | 1 |
| 317 | Biological Process | GO:0009250 | glucan biosynthetic process | 0.25 | 0.007027938 | 0.020218838 | Igf1 | 1 |
| 318 | Biological Process | GO:0031050 | dsRNA fragmentation | 0.25 | 0.007027938 | 0.020218838 | Egfr | 1 |
| 319 | Biological Process | GO:0032768 | regulation of monooxygenase activity | 0.25 | 0.007027938 | 0.020218838 | Egfr | 1 |
| 320 | Biological Process | GO:0032881 | regulation of polysaccharide metabolic process | 0.25 | 0.007027938 | 0.020218838 | Igf1 | 1 |
| 321 | Biological Process | GO:0042307 | positive regulation of protein import into nucleus | 0.25 | 0.007027938 | 0.020218838 | Igf1 | 1 |
| 322 | Biological Process | GO:0060147 | regulation of posttranscriptional gene silencing | 0.25 | 0.007027938 | 0.020218838 | Egfr | 1 |
| 323 | Biological Process | GO:0060966 | regulation of gene silencing by RNA | 0.25 | 0.007027938 | 0.020218838 | Egfr | 1 |
| 324 | Biological Process | GO:0070918 | production of small RNA involved in gene silencing by RNA | 0.25 | 0.007027938 | 0.020218838 | Egfr | 1 |
| 325 | Biological Process | GO:0097720 | calcineurin-mediated signaling | 0.25 | 0.007027938 | 0.020218838 | Igf1 | 1 |
| 326 | Biological Process | GO:0014904 | myotube cell development | 0.25 | 0.007194817 | 0.020509616 | Igf1 | 1 |
| 327 | Biological Process | GO:0030166 | proteoglycan biosynthetic process | 0.25 | 0.007194817 | 0.020509616 | Igf1 | 1 |
| 328 | Biological Process | GO:1901031 | regulation of response to reactive oxygen species | 0.25 | 0.007194817 | 0.020509616 | Hgf | 1 |
| 329 | Biological Process | GO:0050850 | positive regulation of calcium-mediated signaling | 0.25 | 0.007361675 | 0.020921478 | Igf1 | 1 |
| 330 | Biological Process | GO:0030521 | androgen receptor signaling pathway | 0.25 | 0.007528512 | 0.021012415 | Igf1 | 1 |
| 331 | Biological Process | GO:0032715 | negative regulation of interleukin-6 production | 0.25 | 0.007528512 | 0.021012415 | Hgf | 1 |
| 332 | Biological Process | GO:0045907 | positive regulation of vasoconstriction | 0.25 | 0.007528512 | 0.021012415 | Egfr | 1 |
| 333 | Biological Process | GO:0046326 | positive regulation of glucose import | 0.25 | 0.007528512 | 0.021012415 | Igf1 | 1 |
| 334 | Biological Process | GO:0050798 | activated T cell proliferation | 0.25 | 0.007528512 | 0.021012415 | Igf1 | 1 |
| 335 | Biological Process | GO:1903018 | regulation of glycoprotein metabolic process | 0.25 | 0.007528512 | 0.021012415 | Igf1 | 1 |
| 336 | Biological Process | GO:0043470 | regulation of carbohydrate catabolic process | 0.25 | 0.007695328 | 0.021287372 | Igf1 | 1 |
| 337 | Biological Process | GO:0048599 | oocyte development | 0.25 | 0.007695328 | 0.021287372 | Igf1 | 1 |
| 338 | Biological Process | GO:2001169 | regulation of ATP biosynthetic process | 0.25 | 0.007695328 | 0.021287372 | Igf1 | 1 |
| 339 | Biological Process | GO:0042246 | tissue regeneration | 0.25 | 0.007862123 | 0.021431735 | Igf1 | 1 |
| 340 | Biological Process | GO:0048016 | inositol phosphate-mediated signaling | 0.25 | 0.007862123 | 0.021431735 | Igf1 | 1 |
| 341 | Biological Process | GO:0051196 | regulation of coenzyme metabolic process | 0.25 | 0.007862123 | 0.021431735 | Igf1 | 1 |
| 342 | Biological Process | GO:1904036 | negative regulation of epithelial cell apoptotic process | 0.25 | 0.007862123 | 0.021431735 | Igf1 | 1 |
| 343 | Biological Process | GO:1904591 | positive regulation of protein import | 0.25 | 0.007862123 | 0.021431735 | Igf1 | 1 |
| 344 | Biological Process | GO:0031641 | regulation of myelination | 0.25 | 0.008028897 | 0.021696586 | Hgf | 1 |
| 345 | Biological Process | GO:0032653 | regulation of interleukin-10 production | 0.25 | 0.008028897 | 0.021696586 | Hgf | 1 |
| 346 | Biological Process | GO:0045124 | regulation of bone resorption | 0.25 | 0.008028897 | 0.021696586 | Egfr | 1 |
| 347 | Biological Process | GO:0032355 | response to estradiol | 0.25 | 0.00819565 | 0.021769695 | Egfr | 1 |
| 348 | Biological Process | GO:0055025 | positive regulation of cardiac muscle tissue development | 0.25 | 0.00819565 | 0.021769695 | Igf1 | 1 |
| 349 | Biological Process | GO:0060428 | lung epithelium development | 0.25 | 0.00819565 | 0.021769695 | Igf1 | 1 |
| 350 | Biological Process | GO:1902041 | regulation of extrinsic apoptotic signaling pathway via death domain receptors | 0.25 | 0.00819565 | 0.021769695 | Hgf | 1 |
| 351 | Biological Process | GO:1903202 | negative regulation of oxidative stress-induced cell death | 0.25 | 0.00819565 | 0.021769695 | Hgf | 1 |
| 352 | Biological Process | GO:2000677 | regulation of transcription regulatory region DNA binding | 0.25 | 0.00819565 | 0.021769695 | Igf1 | 1 |
| 353 | Biological Process | GO:0009994 | oocyte differentiation | 0.25 | 0.008362382 | 0.021962997 | Igf1 | 1 |
| 354 | Biological Process | GO:0030104 | water homeostasis | 0.25 | 0.008362382 | 0.021962997 | Igf1 | 1 |
| 355 | Biological Process | GO:0032350 | regulation of hormone metabolic process | 0.25 | 0.008362382 | 0.021962997 | Igf1 | 1 |
| 356 | Biological Process | GO:1900408 | negative regulation of cellular response to oxidative stress | 0.25 | 0.008362382 | 0.021962997 | Hgf | 1 |
| 357 | Biological Process | GO:0001974 | blood vessel remodeling | 0.25 | 0.008529093 | 0.022151949 | Igf1 | 1 |
| 358 | Biological Process | GO:0032613 | interleukin-10 production | 0.25 | 0.008529093 | 0.022151949 | Hgf | 1 |
| 359 | Biological Process | GO:0045739 | positive regulation of DNA repair | 0.25 | 0.008529093 | 0.022151949 | Egfr | 1 |
| 360 | Biological Process | GO:0048247 | lymphocyte chemotaxis | 0.25 | 0.008529093 | 0.022151949 | Ccl11 | 1 |
| 361 | Biological Process | GO:0010656 | negative regulation of muscle cell apoptotic process | 0.25 | 0.008695782 | 0.022336694 | Igf1 | 1 |
| 362 | Biological Process | GO:0030850 | prostate gland development | 0.25 | 0.008695782 | 0.022336694 | Igf1 | 1 |
| 363 | Biological Process | GO:0050732 | negative regulation of peptidyl-tyrosine phosphorylation | 0.25 | 0.008695782 | 0.022336694 | Igf1 | 1 |
| 364 | Biological Process | GO:0070098 | chemokine-mediated signaling pathway | 0.25 | 0.008695782 | 0.022336694 | Ccl11 | 1 |
| 365 | Biological Process | GO:0002548 | monocyte chemotaxis | 0.25 | 0.008862451 | 0.022456346 | Ccl11 | 1 |
| 366 | Biological Process | GO:0042531 | positive regulation of tyrosine phosphorylation of STAT protein | 0.25 | 0.008862451 | 0.022456346 | Igf1 | 1 |
| 367 | Biological Process | GO:0046622 | positive regulation of organ growth | 0.25 | 0.008862451 | 0.022456346 | Igf1 | 1 |
| 368 | Biological Process | GO:0048546 | digestive tract morphogenesis | 0.25 | 0.008862451 | 0.022456346 | Egfr | 1 |
| 369 | Biological Process | GO:1902883 | negative regulation of response to oxidative stress | 0.25 | 0.008862451 | 0.022456346 | Hgf | 1 |
| 370 | Biological Process | GO:0010828 | positive regulation of glucose transmembrane transport | 0.25 | 0.009029099 | 0.022755276 | Igf1 | 1 |
| 371 | Biological Process | GO:0021795 | cerebral cortex cell migration | 0.25 | 0.009029099 | 0.022755276 | Egfr | 1 |
| 372 | Biological Process | GO:0038083 | peptidyl-tyrosine autophosphorylation | 0.25 | 0.009195726 | 0.022928009 | Egfr | 1 |
| 373 | Biological Process | GO:0042698 | ovulation cycle | 0.25 | 0.009195726 | 0.022928009 | Egfr | 1 |
| 374 | Biological Process | GO:0046579 | positive regulation of Ras protein signal transduction | 0.25 | 0.009195726 | 0.022928009 | Igf1 | 1 |
| 375 | Biological Process | GO:1903078 | positive regulation of protein localization to plasma membrane | 0.25 | 0.009195726 | 0.022928009 | Egfr | 1 |
| 376 | Biological Process | GO:0000060 | protein import into nucleus, translocation | 0.25 | 0.009362331 | 0.023158148 | Igf1 | 1 |
| 377 | Biological Process | GO:0014911 | positive regulation of smooth muscle cell migration | 0.25 | 0.009362331 | 0.023158148 | Igf1 | 1 |
| 378 | Biological Process | GO:0031102 | neuron projection regeneration | 0.25 | 0.009362331 | 0.023158148 | Hgf | 1 |
| 379 | Biological Process | GO:0045600 | positive regulation of fat cell differentiation | 0.25 | 0.009528916 | 0.023508012 | Igf1 | 1 |
| 380 | Biological Process | GO:0046850 | regulation of bone remodeling | 0.25 | 0.00969548 | 0.023731083 | Egfr | 1 |
| 381 | Biological Process | GO:0048286 | lung alveolus development | 0.25 | 0.00969548 | 0.023731083 | Igf1 | 1 |
| 382 | Biological Process | GO:0060443 | mammary gland morphogenesis | 0.25 | 0.00969548 | 0.023731083 | Ccl11 | 1 |
| 383 | Biological Process | GO:0006801 | superoxide metabolic process | 0.25 | 0.009862022 | 0.023950626 | Egfr | 1 |
| 384 | Biological Process | GO:0045840 | positive regulation of mitotic nuclear division | 0.25 | 0.009862022 | 0.023950626 | Igf1 | 1 |
| 385 | Biological Process | GO:0050982 | detection of mechanical stimulus | 0.25 | 0.009862022 | 0.023950626 | Igf1 | 1 |
| 386 | Biological Process | GO:0010611 | regulation of cardiac muscle hypertrophy | 0.25 | 0.010028544 | 0.024166724 | Igf1 | 1 |
| 387 | Biological Process | GO:0033692 | cellular polysaccharide biosynthetic process | 0.25 | 0.010028544 | 0.024166724 | Igf1 | 1 |
| 388 | Biological Process | GO:0051193 | regulation of cofactor metabolic process | 0.25 | 0.010028544 | 0.024166724 | Igf1 | 1 |
| 389 | Biological Process | GO:0010676 | positive regulation of cellular carbohydrate metabolic process | 0.25 | 0.010195045 | 0.024317263 | Igf1 | 1 |
| 390 | Biological Process | GO:0043388 | positive regulation of DNA binding | 0.25 | 0.010195045 | 0.024317263 | Igf1 | 1 |
| 391 | Biological Process | GO:0046626 | regulation of insulin receptor signaling pathway | 0.25 | 0.010195045 | 0.024317263 | Igf1 | 1 |
| 392 | Biological Process | GO:1904377 | positive regulation of protein localization to cell periphery | 0.25 | 0.010195045 | 0.024317263 | Egfr | 1 |
| 393 | Biological Process | GO:0014743 | regulation of muscle hypertrophy | 0.25 | 0.010361525 | 0.024341773 | Igf1 | 1 |
| 394 | Biological Process | GO:0045428 | regulation of nitric oxide biosynthetic process | 0.25 | 0.010361525 | 0.024341773 | Igf1 | 1 |
| 395 | Biological Process | GO:0051057 | positive regulation of small GTPase mediated signal transduction | 0.25 | 0.010361525 | 0.024341773 | Igf1 | 1 |
| 396 | Biological Process | GO:1901224 | positive regulation of NIK/NF-kappaB signaling | 0.25 | 0.010361525 | 0.024341773 | Egfr | 1 |
| 397 | Biological Process | GO:2000179 | positive regulation of neural precursor cell proliferation | 0.25 | 0.010361525 | 0.024341773 | Igf1 | 1 |
| 398 | Biological Process | GO:2000242 | negative regulation of reproductive process | 0.25 | 0.010361525 | 0.024341773 | Igf1 | 1 |
| 399 | Biological Process | GO:0030810 | positive regulation of nucleotide biosynthetic process | 0.25 | 0.010694421 | 0.024812118 | Igf1 | 1 |
| 400 | Biological Process | GO:0046824 | positive regulation of nucleocytoplasmic transport | 0.25 | 0.010694421 | 0.024812118 | Igf1 | 1 |
| 401 | Biological Process | GO:0060425 | lung morphogenesis | 0.25 | 0.010694421 | 0.024812118 | Igf1 | 1 |
| 402 | Biological Process | GO:0071359 | cellular response to dsRNA | 0.25 | 0.010694421 | 0.024812118 | Egfr | 1 |
| 403 | Biological Process | GO:1900373 | positive regulation of purine nucleotide biosynthetic process | 0.25 | 0.010694421 | 0.024812118 | Igf1 | 1 |
| 404 | Biological Process | GO:0055021 | regulation of cardiac muscle tissue growth | 0.25 | 0.011027233 | 0.025395229 | Igf1 | 1 |
| 405 | Biological Process | GO:0060688 | regulation of morphogenesis of a branching structure | 0.25 | 0.011027233 | 0.025395229 | Hgf | 1 |
| 406 | Biological Process | GO:1903578 | regulation of ATP metabolic process | 0.25 | 0.011027233 | 0.025395229 | Igf1 | 1 |
| 407 | Biological Process | GO:0006029 | proteoglycan metabolic process | 0.25 | 0.011193608 | 0.025652018 | Igf1 | 1 |
| 408 | Biological Process | GO:0045669 | positive regulation of osteoblast differentiation | 0.25 | 0.011193608 | 0.025652018 | Igf1 | 1 |
| 409 | Biological Process | GO:0000079 | regulation of cyclin-dependent protein serine/threonine kinase activity | 0.25 | 0.011359962 | 0.025780496 | Egfr | 1 |
| 410 | Biological Process | GO:0042306 | regulation of protein import into nucleus | 0.25 | 0.011359962 | 0.025780496 | Igf1 | 1 |
| 411 | Biological Process | GO:0042509 | regulation of tyrosine phosphorylation of STAT protein | 0.25 | 0.011359962 | 0.025780496 | Igf1 | 1 |
| 412 | Biological Process | GO:0070373 | negative regulation of ERK1 and ERK2 cascade | 0.25 | 0.011359962 | 0.025780496 | Igf1 | 1 |
| 413 | Biological Process | GO:0000271 | polysaccharide biosynthetic process | 0.25 | 0.011526294 | 0.025844329 | Igf1 | 1 |
| 414 | Biological Process | GO:0022029 | telencephalon cell migration | 0.25 | 0.011526294 | 0.025844329 | Egfr | 1 |
| 415 | Biological Process | GO:0050810 | regulation of steroid biosynthetic process | 0.25 | 0.011526294 | 0.025844329 | Igf1 | 1 |
| 416 | Biological Process | GO:0051155 | positive regulation of striated muscle cell differentiation | 0.25 | 0.011526294 | 0.025844329 | Igf1 | 1 |
| 417 | Biological Process | GO:2000573 | positive regulation of DNA biosynthetic process | 0.25 | 0.011526294 | 0.025844329 | Hgf | 1 |
| 418 | Biological Process | GO:0006096 | glycolytic process | 0.25 | 0.011692606 | 0.026029968 | Igf1 | 1 |
| 419 | Biological Process | GO:0006809 | nitric oxide biosynthetic process | 0.25 | 0.011692606 | 0.026029968 | Igf1 | 1 |
| 420 | Biological Process | GO:0045453 | bone resorption | 0.25 | 0.011692606 | 0.026029968 | Egfr | 1 |
| 421 | Biological Process | GO:0006757 | ATP generation from ADP | 0.25 | 0.011858897 | 0.026212928 | Igf1 | 1 |
| 422 | Biological Process | GO:0007260 | tyrosine phosphorylation of STAT protein | 0.25 | 0.011858897 | 0.026212928 | Igf1 | 1 |
| 423 | Biological Process | GO:0034637 | cellular carbohydrate biosynthetic process | 0.25 | 0.011858897 | 0.026212928 | Igf1 | 1 |
| 424 | Biological Process | GO:0021885 | forebrain cell migration | 0.25 | 0.012025167 | 0.026386512 | Egfr | 1 |
| 425 | Biological Process | GO:0042446 | hormone biosynthetic process | 0.25 | 0.012025167 | 0.026386512 | Igf1 | 1 |
| 426 | Biological Process | GO:1903201 | regulation of oxidative stress-induced cell death | 0.25 | 0.012025167 | 0.026386512 | Hgf | 1 |
| 427 | Biological Process | GO:0046209 | nitric oxide metabolic process | 0.25 | 0.012191415 | 0.026386512 | Igf1 | 1 |
| 428 | Biological Process | GO:0046324 | regulation of glucose import | 0.25 | 0.012191415 | 0.026386512 | Igf1 | 1 |
| 429 | Biological Process | GO:0060420 | regulation of heart growth | 0.25 | 0.012191415 | 0.026386512 | Igf1 | 1 |
| 430 | Biological Process | GO:0060968 | regulation of gene silencing | 0.25 | 0.012191415 | 0.026386512 | Egfr | 1 |
| 431 | Biological Process | GO:1904029 | regulation of cyclin-dependent protein kinase activity | 0.25 | 0.012191415 | 0.026386512 | Egfr | 1 |
| 432 | Biological Process | GO:1904589 | regulation of protein import | 0.25 | 0.012191415 | 0.026386512 | Igf1 | 1 |
| 433 | Biological Process | GO:0008625 | extrinsic apoptotic signaling pathway via death domain receptors | 0.25 | 0.012357643 | 0.026440266 | Hgf | 1 |
| 434 | Biological Process | GO:0014015 | positive regulation of gliogenesis | 0.25 | 0.012357643 | 0.026440266 | Igf1 | 1 |
| 435 | Biological Process | GO:0042866 | pyruvate biosynthetic process | 0.25 | 0.012357643 | 0.026440266 | Igf1 | 1 |
| 436 | Biological Process | GO:0051785 | positive regulation of nuclear division | 0.25 | 0.012357643 | 0.026440266 | Igf1 | 1 |
| 437 | Biological Process | GO:0071674 | mononuclear cell migration | 0.25 | 0.012357643 | 0.026440266 | Ccl11 | 1 |
| 438 | Biological Process | GO:0019229 | regulation of vasoconstriction | 0.25 | 0.01252385 | 0.026552834 | Egfr | 1 |
| 439 | Biological Process | GO:0033143 | regulation of intracellular steroid hormone receptor signaling pathway | 0.25 | 0.01252385 | 0.026552834 | Igf1 | 1 |
| 440 | Biological Process | GO:0071347 | cellular response to interleukin-1 | 0.25 | 0.01252385 | 0.026552834 | Ccl11 | 1 |
| 441 | Biological Process | GO:0097755 | positive regulation of blood vessel diameter | 0.25 | 0.01252385 | 0.026552834 | Egfr | 1 |
| 442 | Biological Process | GO:0005977 | glycogen metabolic process | 0.25 | 0.012690036 | 0.02660355 | Igf1 | 1 |
| 443 | Biological Process | GO:0006073 | cellular glucan metabolic process | 0.25 | 0.012690036 | 0.02660355 | Igf1 | 1 |
| 444 | Biological Process | GO:0044042 | glucan metabolic process | 0.25 | 0.012690036 | 0.02660355 | Igf1 | 1 |
| 445 | Biological Process | GO:0045913 | positive regulation of carbohydrate metabolic process | 0.25 | 0.012690036 | 0.02660355 | Igf1 | 1 |
| 446 | Biological Process | GO:0061180 | mammary gland epithelium development | 0.25 | 0.012690036 | 0.02660355 | Ccl11 | 1 |
| 447 | Biological Process | GO:0051341 | regulation of oxidoreductase activity | 0.25 | 0.012856201 | 0.026878349 | Egfr | 1 |
| 448 | Biological Process | GO:0045844 | positive regulation of striated muscle tissue development | 0.25 | 0.013022344 | 0.026878349 | Igf1 | 1 |
| 449 | Biological Process | GO:0046031 | ADP metabolic process | 0.25 | 0.013022344 | 0.026878349 | Igf1 | 1 |
| 450 | Biological Process | GO:0048636 | positive regulation of muscle organ development | 0.25 | 0.013022344 | 0.026878349 | Igf1 | 1 |
| 451 | Biological Process | GO:0048708 | astrocyte differentiation | 0.25 | 0.013022344 | 0.026878349 | Egfr | 1 |
| 452 | Biological Process | GO:0051966 | regulation of synaptic transmission, glutamatergic | 0.25 | 0.013022344 | 0.026878349 | Egfr | 1 |
| 453 | Biological Process | GO:2001057 | reactive nitrogen species metabolic process | 0.25 | 0.013022344 | 0.026878349 | Igf1 | 1 |
| 454 | Biological Process | GO:0034103 | regulation of tissue remodeling | 0.25 | 0.013188467 | 0.026924055 | Egfr | 1 |
| 455 | Biological Process | GO:0038034 | signal transduction in absence of ligand | 0.25 | 0.013188467 | 0.026924055 | Igf1 | 1 |
| 456 | Biological Process | GO:0097192 | extrinsic apoptotic signaling pathway in absence of ligand | 0.25 | 0.013188467 | 0.026924055 | Igf1 | 1 |
| 457 | Biological Process | GO:1901863 | positive regulation of muscle tissue development | 0.25 | 0.013188467 | 0.026924055 | Igf1 | 1 |
| 458 | Biological Process | GO:1904035 | regulation of epithelial cell apoptotic process | 0.25 | 0.013188467 | 0.026924055 | Igf1 | 1 |
| 459 | Biological Process | GO:0014910 | regulation of smooth muscle cell migration | 0.25 | 0.013354569 | 0.027085732 | Igf1 | 1 |
| 460 | Biological Process | GO:0070301 | cellular response to hydrogen peroxide | 0.25 | 0.013354569 | 0.027085732 | Hgf | 1 |
| 461 | Biological Process | GO:1900182 | positive regulation of protein localization to nucleus | 0.25 | 0.013354569 | 0.027085732 | Igf1 | 1 |
| 462 | Biological Process | GO:0001938 | positive regulation of endothelial cell proliferation | 0.25 | 0.01352065 | 0.027304121 | Ccl11 | 1 |
| 463 | Biological Process | GO:1900407 | regulation of cellular response to oxidative stress | 0.25 | 0.01352065 | 0.027304121 | Hgf | 1 |
| 464 | Biological Process | GO:0009135 | purine nucleoside diphosphate metabolic process | 0.25 | 0.013852749 | 0.027616888 | Igf1 | 1 |
| 465 | Biological Process | GO:0009179 | purine ribonucleoside diphosphate metabolic process | 0.25 | 0.013852749 | 0.027616888 | Igf1 | 1 |
| 466 | Biological Process | GO:0046889 | positive regulation of lipid biosynthetic process | 0.25 | 0.013852749 | 0.027616888 | Igf1 | 1 |
| 467 | Biological Process | GO:0051149 | positive regulation of muscle cell differentiation | 0.25 | 0.013852749 | 0.027616888 | Igf1 | 1 |
| 468 | Biological Process | GO:0055013 | cardiac muscle cell development | 0.25 | 0.013852749 | 0.027616888 | Igf1 | 1 |
| 469 | Biological Process | GO:1900076 | regulation of cellular response to insulin stimulus | 0.25 | 0.013852749 | 0.027616888 | Igf1 | 1 |
| 470 | Biological Process | GO:0010660 | regulation of muscle cell apoptotic process | 0.25 | 0.014018767 | 0.027770228 | Igf1 | 1 |
| 471 | Biological Process | GO:0019359 | nicotinamide nucleotide biosynthetic process | 0.25 | 0.014018767 | 0.027770228 | Igf1 | 1 |
| 472 | Biological Process | GO:1903524 | positive regulation of blood circulation | 0.25 | 0.014018767 | 0.027770228 | Egfr | 1 |
| 473 | Biological Process | GO:0043502 | regulation of muscle adaptation | 0.25 | 0.014184764 | 0.027804517 | Igf1 | 1 |
| 474 | Biological Process | GO:0045471 | response to ethanol | 0.25 | 0.014184764 | 0.027804517 | Igf1 | 1 |
| 475 | Biological Process | GO:0045981 | positive regulation of nucleotide metabolic process | 0.25 | 0.014184764 | 0.027804517 | Igf1 | 1 |
| 476 | Biological Process | GO:1900544 | positive regulation of purine nucleotide metabolic process | 0.25 | 0.014184764 | 0.027804517 | Igf1 | 1 |
| 477 | Biological Process | GO:2001022 | positive regulation of response to DNA damage stimulus | 0.25 | 0.014184764 | 0.027804517 | Egfr | 1 |
| 478 | Biological Process | GO:0006112 | energy reserve metabolic process | 0.25 | 0.014350741 | 0.027990701 | Igf1 | 1 |
| 479 | Biological Process | GO:0006165 | nucleoside diphosphate phosphorylation | 0.25 | 0.014350741 | 0.027990701 | Igf1 | 1 |
| 480 | Biological Process | GO:0009185 | ribonucleoside diphosphate metabolic process | 0.25 | 0.014516696 | 0.027990701 | Igf1 | 1 |
| 481 | Biological Process | GO:0014909 | smooth muscle cell migration | 0.25 | 0.014516696 | 0.027990701 | Igf1 | 1 |
| 482 | Biological Process | GO:0019363 | pyridine nucleotide biosynthetic process | 0.25 | 0.014516696 | 0.027990701 | Igf1 | 1 |
| 483 | Biological Process | GO:0031016 | pancreas development | 0.25 | 0.014516696 | 0.027990701 | Igf1 | 1 |
| 484 | Biological Process | GO:0046323 | glucose import | 0.25 | 0.014516696 | 0.027990701 | Igf1 | 1 |
| 485 | Biological Process | GO:0030593 | neutrophil chemotaxis | 0.25 | 0.01468263 | 0.027990701 | Ccl11 | 1 |
| 486 | Biological Process | GO:0046939 | nucleotide phosphorylation | 0.25 | 0.01468263 | 0.027990701 | Igf1 | 1 |
| 487 | Biological Process | GO:0048477 | oogenesis | 0.25 | 0.01468263 | 0.027990701 | Igf1 | 1 |
| 488 | Biological Process | GO:0072676 | lymphocyte migration | 0.25 | 0.01468263 | 0.027990701 | Ccl11 | 1 |
| 489 | Biological Process | GO:1901222 | regulation of NIK/NF-kappaB signaling | 0.25 | 0.01468263 | 0.027990701 | Egfr | 1 |
| 490 | Biological Process | GO:1903426 | regulation of reactive oxygen species biosynthetic process | 0.25 | 0.01468263 | 0.027990701 | Igf1 | 1 |
| 491 | Biological Process | GO:0003300 | cardiac muscle hypertrophy | 0.25 | 0.014848543 | 0.027990701 | Igf1 | 1 |
| 492 | Biological Process | GO:0010657 | muscle cell apoptotic process | 0.25 | 0.014848543 | 0.027990701 | Igf1 | 1 |
| 493 | Biological Process | GO:0036473 | cell death in response to oxidative stress | 0.25 | 0.014848543 | 0.027990701 | Hgf | 1 |
| 494 | Biological Process | GO:0055006 | cardiac cell development | 0.25 | 0.014848543 | 0.027990701 | Igf1 | 1 |
| 495 | Biological Process | GO:0055024 | regulation of cardiac muscle tissue development | 0.25 | 0.014848543 | 0.027990701 | Igf1 | 1 |
| 496 | Biological Process | GO:0070555 | response to interleukin-1 | 0.25 | 0.014848543 | 0.027990701 | Ccl11 | 1 |
| 497 | Biological Process | GO:0050848 | regulation of calcium-mediated signaling | 0.25 | 0.015014435 | 0.028189753 | Igf1 | 1 |
| 498 | Biological Process | GO:1902882 | regulation of response to oxidative stress | 0.25 | 0.015014435 | 0.028189753 | Hgf | 1 |
| 499 | Biological Process | GO:0045445 | myoblast differentiation | 0.25 | 0.015180307 | 0.028387174 | Igf1 | 1 |
| 500 | Biological Process | GO:0072525 | pyridine-containing compound biosynthetic process | 0.25 | 0.015180307 | 0.028387174 | Igf1 | 1 |
| 501 | Biological Process | GO:0014897 | striated muscle hypertrophy | 0.25 | 0.015346157 | 0.028582982 | Igf1 | 1 |
| 502 | Biological Process | GO:0055017 | cardiac muscle tissue growth | 0.25 | 0.015346157 | 0.028582982 | Igf1 | 1 |
| 503 | Biological Process | GO:0010827 | regulation of glucose transmembrane transport | 0.25 | 0.015511987 | 0.028663454 | Igf1 | 1 |
| 504 | Biological Process | GO:0021782 | glial cell development | 0.25 | 0.015511987 | 0.028663454 | Egfr | 1 |
| 505 | Biological Process | GO:0030838 | positive regulation of actin filament polymerization | 0.25 | 0.015511987 | 0.028663454 | Ccl11 | 1 |
| 506 | Biological Process | GO:1905477 | positive regulation of protein localization to membrane | 0.25 | 0.015511987 | 0.028663454 | Egfr | 1 |
| 507 | Biological Process | GO:0014896 | muscle hypertrophy | 0.25 | 0.015677795 | 0.028855784 | Igf1 | 1 |
| 508 | Biological Process | GO:0038061 | NIK/NF-kappaB signaling | 0.25 | 0.015677795 | 0.028855784 | Egfr | 1 |
| 509 | Biological Process | GO:0031644 | regulation of neurological system process | 0.25 | 0.015843583 | 0.028953079 | Hgf | 1 |
| 510 | Biological Process | GO:0043255 | regulation of carbohydrate biosynthetic process | 0.25 | 0.015843583 | 0.028953079 | Igf1 | 1 |
| 511 | Biological Process | GO:0046849 | bone remodeling | 0.25 | 0.015843583 | 0.028953079 | Egfr | 1 |
| 512 | Biological Process | GO:0006282 | regulation of DNA repair | 0.25 | 0.016009349 | 0.028953079 | Egfr | 1 |
| 513 | Biological Process | GO:0019218 | regulation of steroid metabolic process | 0.25 | 0.016009349 | 0.028953079 | Igf1 | 1 |
| 514 | Biological Process | GO:0042102 | positive regulation of T cell proliferation | 0.25 | 0.016009349 | 0.028953079 | Igf1 | 1 |
| 515 | Biological Process | GO:0044264 | cellular polysaccharide metabolic process | 0.25 | 0.016009349 | 0.028953079 | Igf1 | 1 |
| 516 | Biological Process | GO:0045778 | positive regulation of ossification | 0.25 | 0.016009349 | 0.028953079 | Igf1 | 1 |
| 517 | Biological Process | GO:0090263 | positive regulation of canonical Wnt signaling pathway | 0.25 | 0.016009349 | 0.028953079 | Egfr | 1 |
| 518 | Biological Process | GO:1903076 | regulation of protein localization to plasma membrane | 0.25 | 0.016175095 | 0.029196359 | Egfr | 1 |
| 519 | Biological Process | GO:0001824 | blastocyst development | 0.25 | 0.01634082 | 0.029269476 | Igf1 | 1 |
| 520 | Biological Process | GO:0006090 | pyruvate metabolic process | 0.25 | 0.01634082 | 0.029269476 | Igf1 | 1 |
| 521 | Biological Process | GO:0042310 | vasoconstriction | 0.25 | 0.01634082 | 0.029269476 | Egfr | 1 |
| 522 | Biological Process | GO:0046427 | positive regulation of JAK-STAT cascade | 0.25 | 0.01634082 | 0.029269476 | Igf1 | 1 |
| 523 | Biological Process | GO:0014812 | muscle cell migration | 0.25 | 0.016506524 | 0.029453434 | Igf1 | 1 |
| 524 | Biological Process | GO:0042136 | neurotransmitter biosynthetic process | 0.25 | 0.016506524 | 0.029453434 | Igf1 | 1 |
| 525 | Biological Process | GO:0007173 | epidermal growth factor receptor signaling pathway | 0.25 | 0.016672206 | 0.029523699 | Egfr | 1 |
| 526 | Biological Process | GO:0009408 | response to heat | 0.25 | 0.016672206 | 0.029523699 | Igf1 | 1 |
| 527 | Biological Process | GO:0043467 | regulation of generation of precursor metabolites and energy | 0.25 | 0.016672206 | 0.029523699 | Igf1 | 1 |
| 528 | Biological Process | GO:1904894 | positive regulation of STAT cascade | 0.25 | 0.016672206 | 0.029523699 | Igf1 | 1 |
| 529 | Biological Process | GO:0030282 | bone mineralization | 0.25 | 0.016837868 | 0.029648601 | Igf1 | 1 |
| 530 | Biological Process | GO:0060419 | heart growth | 0.25 | 0.016837868 | 0.029648601 | Igf1 | 1 |
| 531 | Biological Process | GO:2000379 | positive regulation of reactive oxygen species metabolic process | 0.25 | 0.016837868 | 0.029648601 | Egfr | 1 |
| 532 | Biological Process | GO:1904019 | epithelial cell apoptotic process | 0.25 | 0.017003509 | 0.02982792 | Igf1 | 1 |
| 533 | Biological Process | GO:2001023 | regulation of response to drug | 0.25 | 0.017003509 | 0.02982792 | Hgf | 1 |
| 534 | Biological Process | GO:0043500 | muscle adaptation | 0.25 | 0.017169129 | 0.030005862 | Igf1 | 1 |
| 535 | Biological Process | GO:0051592 | response to calcium ion | 0.25 | 0.017169129 | 0.030005862 | Egfr | 1 |
| 536 | Biological Process | GO:0009132 | nucleoside diphosphate metabolic process | 0.25 | 0.017334729 | 0.030126341 | Igf1 | 1 |
| 537 | Biological Process | GO:0097756 | negative regulation of blood vessel diameter | 0.25 | 0.017334729 | 0.030126341 | Egfr | 1 |
| 538 | Biological Process | GO:1903409 | reactive oxygen species biosynthetic process | 0.25 | 0.017334729 | 0.030126341 | Igf1 | 1 |
| 539 | Biological Process | GO:0021987 | cerebral cortex development | 0.25 | 0.017500307 | 0.030245447 | Egfr | 1 |
| 540 | Biological Process | GO:1904375 | regulation of protein localization to cell periphery | 0.25 | 0.017500307 | 0.030245447 | Egfr | 1 |
| 541 | Biological Process | GO:2000177 | regulation of neural precursor cell proliferation | 0.25 | 0.017500307 | 0.030245447 | Igf1 | 1 |
| 542 | Biological Process | GO:1990266 | neutrophil migration | 0.25 | 0.017665864 | 0.030475245 | Ccl11 | 1 |
| 543 | Biological Process | GO:0008286 | insulin receptor signaling pathway | 0.25 | 0.0178314 | 0.03064772 | Igf1 | 1 |
| 544 | Biological Process | GO:0046620 | regulation of organ growth | 0.25 | 0.0178314 | 0.03064772 | Igf1 | 1 |
| 545 | Biological Process | GO:0005976 | polysaccharide metabolic process | 0.25 | 0.017996916 | 0.030706417 | Igf1 | 1 |
| 546 | Biological Process | GO:0035249 | synaptic transmission, glutamatergic | 0.25 | 0.017996916 | 0.030706417 | Egfr | 1 |
| 547 | Biological Process | GO:0071346 | cellular response to interferon-gamma | 0.25 | 0.017996916 | 0.030706417 | Ccl11 | 1 |
| 548 | Biological Process | GO:2000278 | regulation of DNA biosynthetic process | 0.25 | 0.017996916 | 0.030706417 | Hgf | 1 |
| 549 | Biological Process | GO:0038127 | ERBB signaling pathway | 0.25 | 0.01816241 | 0.030820061 | Egfr | 1 |
| 550 | Biological Process | GO:0071621 | granulocyte chemotaxis | 0.25 | 0.01816241 | 0.030820061 | Ccl11 | 1 |
| 551 | Biological Process | GO:1905330 | regulation of morphogenesis of an epithelium | 0.25 | 0.01816241 | 0.030820061 | Hgf | 1 |
| 552 | Biological Process | GO:0030518 | intracellular steroid hormone receptor signaling pathway | 0.25 | 0.018327884 | 0.030988375 | Igf1 | 1 |
| 553 | Biological Process | GO:2000045 | regulation of G1/S transition of mitotic cell cycle | 0.25 | 0.018327884 | 0.030988375 | Egfr | 1 |
| 554 | Biological Process | GO:0001942 | hair follicle development | 0.25 | 0.018493337 | 0.031155441 | Egfr | 1 |
| 555 | Biological Process | GO:0042542 | response to hydrogen peroxide | 0.25 | 0.018493337 | 0.031155441 | Hgf | 1 |
| 556 | Biological Process | GO:0043331 | response to dsRNA | 0.25 | 0.018658768 | 0.031377605 | Egfr | 1 |
| 557 | Biological Process | GO:0001892 | embryonic placenta development | 0.25 | 0.018824179 | 0.031429656 | Egfr | 1 |
| 558 | Biological Process | GO:0010906 | regulation of glucose metabolic process | 0.25 | 0.018824179 | 0.031429656 | Igf1 | 1 |
| 559 | Biological Process | GO:0090277 | positive regulation of peptide hormone secretion | 0.25 | 0.018824179 | 0.031429656 | Egfr | 1 |
| 560 | Biological Process | GO:0098773 | skin epidermis development | 0.25 | 0.018824179 | 0.031429656 | Egfr | 1 |
| 561 | Biological Process | GO:0022404 | molting cycle process | 0.25 | 0.018989569 | 0.031480935 | Egfr | 1 |
| 562 | Biological Process | GO:0022405 | hair cycle process | 0.25 | 0.018989569 | 0.031480935 | Egfr | 1 |
| 563 | Biological Process | GO:0042177 | negative regulation of protein catabolic process | 0.25 | 0.018989569 | 0.031480935 | Egfr | 1 |
| 564 | Biological Process | GO:0051153 | regulation of striated muscle cell differentiation | 0.25 | 0.018989569 | 0.031480935 | Igf1 | 1 |
| 565 | Biological Process | GO:0001889 | liver development | 0.25 | 0.019154938 | 0.03169888 | Hgf | 1 |
| 566 | Biological Process | GO:0014902 | myotube differentiation | 0.25 | 0.019320286 | 0.031916021 | Igf1 | 1 |
| 567 | Biological Process | GO:0006754 | ATP biosynthetic process | 0.25 | 0.019485614 | 0.032019418 | Igf1 | 1 |
| 568 | Biological Process | GO:0016052 | carbohydrate catabolic process | 0.25 | 0.019485614 | 0.032019418 | Igf1 | 1 |
| 569 | Biological Process | GO:1900371 | regulation of purine nucleotide biosynthetic process | 0.25 | 0.019485614 | 0.032019418 | Igf1 | 1 |
| 570 | Biological Process | GO:0001936 | regulation of endothelial cell proliferation | 0.25 | 0.01965092 | 0.032065637 | Ccl11 | 1 |
| 571 | Biological Process | GO:0030808 | regulation of nucleotide biosynthetic process | 0.25 | 0.01965092 | 0.032065637 | Igf1 | 1 |
| 572 | Biological Process | GO:0050729 | positive regulation of inflammatory response | 0.25 | 0.01965092 | 0.032065637 | Egfr | 1 |
| 573 | Biological Process | GO:0061008 | hepaticobiliary system development | 0.25 | 0.01965092 | 0.032065637 | Hgf | 1 |
| 574 | Biological Process | GO:0046822 | regulation of nucleocytoplasmic transport | 0.25 | 0.019816205 | 0.03227901 | Igf1 | 1 |
| 575 | Biological Process | GO:0033138 | positive regulation of peptidyl-serine phosphorylation | 0.25 | 0.01998147 | 0.032491607 | Egfr | 1 |
| 576 | Biological Process | GO:0071248 | cellular response to metal ion | 0.25 | 0.020146713 | 0.032703432 | Egfr | 1 |
| 577 | Biological Process | GO:0009166 | nucleotide catabolic process | 0.25 | 0.020311936 | 0.032728417 | Igf1 | 1 |
| 578 | Biological Process | GO:0009582 | detection of abiotic stimulus | 0.25 | 0.020311936 | 0.032728417 | Igf1 | 1 |
| 579 | Biological Process | GO:0051101 | regulation of DNA binding | 0.25 | 0.020311936 | 0.032728417 | Igf1 | 1 |
| 580 | Biological Process | GO:0009581 | detection of external stimulus | 0.25 | 0.020477138 | 0.032728417 | Igf1 | 1 |
| 581 | Biological Process | GO:0045995 | regulation of embryonic development | 0.25 | 0.020477138 | 0.032728417 | Igf1 | 1 |
| 582 | Biological Process | GO:0048565 | digestive tract development | 0.25 | 0.020477138 | 0.032728417 | Egfr | 1 |
| 583 | Biological Process | GO:1900180 | regulation of protein localization to nucleus | 0.25 | 0.020477138 | 0.032728417 | Igf1 | 1 |
| 584 | Biological Process | GO:1902806 | regulation of cell cycle G1/S phase transition | 0.25 | 0.020477138 | 0.032728417 | Egfr | 1 |
| 585 | Biological Process | GO:1904659 | glucose transmembrane transport | 0.25 | 0.020477138 | 0.032728417 | Igf1 | 1 |
| 586 | Biological Process | GO:0035195 | gene silencing by miRNA | 0.25 | 0.020642319 | 0.032880014 | Egfr | 1 |
| 587 | Biological Process | GO:0071333 | cellular response to glucose stimulus | 0.25 | 0.020642319 | 0.032880014 | Igf1 | 1 |
| 588 | Biological Process | GO:0008645 | hexose transmembrane transport | 0.25 | 0.020807479 | 0.033030548 | Igf1 | 1 |
| 589 | Biological Process | GO:0032273 | positive regulation of protein polymerization | 0.25 | 0.020807479 | 0.033030548 | Ccl11 | 1 |
| 590 | Biological Process | GO:0030177 | positive regulation of Wnt signaling pathway | 0.25 | 0.020972618 | 0.03318003 | Egfr | 1 |
| 591 | Biological Process | GO:0071331 | cellular response to hexose stimulus | 0.25 | 0.020972618 | 0.03318003 | Igf1 | 1 |
| 592 | Biological Process | GO:0015749 | monosaccharide transmembrane transport | 0.25 | 0.021137736 | 0.033272363 | Igf1 | 1 |
| 593 | Biological Process | GO:0035194 | posttranscriptional gene silencing by RNA | 0.25 | 0.021137736 | 0.033272363 | Egfr | 1 |
| 594 | Biological Process | GO:0071326 | cellular response to monosaccharide stimulus | 0.25 | 0.021137736 | 0.033272363 | Igf1 | 1 |
| 595 | Biological Process | GO:0009206 | purine ribonucleoside triphosphate biosynthetic process | 0.25 | 0.021302834 | 0.033363734 | Igf1 | 1 |
| 596 | Biological Process | GO:0045598 | regulation of fat cell differentiation | 0.25 | 0.021302834 | 0.033363734 | Igf1 | 1 |
| 597 | Biological Process | GO:0055007 | cardiac muscle cell differentiation | 0.25 | 0.021302834 | 0.033363734 | Igf1 | 1 |
| 598 | Biological Process | GO:0009145 | purine nucleoside triphosphate biosynthetic process | 0.25 | 0.02146791 | 0.03351001 | Igf1 | 1 |
| 599 | Biological Process | GO:0097530 | granulocyte migration | 0.25 | 0.02146791 | 0.03351001 | Ccl11 | 1 |
| 600 | Biological Process | GO:0016441 | posttranscriptional gene silencing | 0.25 | 0.021632966 | 0.033599374 | Egfr | 1 |
| 601 | Biological Process | GO:0032675 | regulation of interleukin-6 production | 0.25 | 0.021632966 | 0.033599374 | Hgf | 1 |
| 602 | Biological Process | GO:0034219 | carbohydrate transmembrane transport | 0.25 | 0.021632966 | 0.033599374 | Igf1 | 1 |
| 603 | Biological Process | GO:0009201 | ribonucleoside triphosphate biosynthetic process | 0.25 | 0.021798 | 0.033632228 | Igf1 | 1 |
| 604 | Biological Process | GO:0034341 | response to interferon-gamma | 0.25 | 0.021798 | 0.033632228 | Ccl11 | 1 |
| 605 | Biological Process | GO:0042303 | molting cycle | 0.25 | 0.021798 | 0.033632228 | Egfr | 1 |
| 606 | Biological Process | GO:0042633 | hair cycle | 0.25 | 0.021798 | 0.033632228 | Egfr | 1 |
| 607 | Biological Process | GO:0001935 | endothelial cell proliferation | 0.25 | 0.021963014 | 0.033719899 | Ccl11 | 1 |
| 608 | Biological Process | GO:0007613 | memory | 0.25 | 0.021963014 | 0.033719899 | Igf1 | 1 |
| 609 | Biological Process | GO:0031214 | biomineral tissue development | 0.25 | 0.021963014 | 0.033719899 | Igf1 | 1 |
| 610 | Biological Process | GO:0009411 | response to UV | 0.25 | 0.022292979 | 0.034003158 | Egfr | 1 |
| 611 | Biological Process | GO:0014013 | regulation of gliogenesis | 0.25 | 0.022292979 | 0.034003158 | Igf1 | 1 |
| 612 | Biological Process | GO:0071322 | cellular response to carbohydrate stimulus | 0.25 | 0.022292979 | 0.034003158 | Igf1 | 1 |
| 613 | Biological Process | GO:1901292 | nucleoside phosphate catabolic process | 0.25 | 0.022292979 | 0.034003158 | Igf1 | 1 |
| 614 | Biological Process | GO:0046496 | nicotinamide nucleotide metabolic process | 0.25 | 0.022457931 | 0.034143358 | Igf1 | 1 |
| 615 | Biological Process | GO:0097305 | response to alcohol | 0.25 | 0.022457931 | 0.034143358 | Igf1 | 1 |
| 616 | Biological Process | GO:0045667 | regulation of osteoblast differentiation | 0.25 | 0.022622861 | 0.034310089 | Igf1 | 1 |
| 617 | Biological Process | GO:0009127 | purine nucleoside monophosphate biosynthetic process | 0.25 | 0.02278777 | 0.034310089 | Igf1 | 1 |
| 618 | Biological Process | GO:0009168 | purine ribonucleoside monophosphate biosynthetic process | 0.25 | 0.02278777 | 0.034310089 | Igf1 | 1 |
| 619 | Biological Process | GO:0019362 | pyridine nucleotide metabolic process | 0.25 | 0.02278777 | 0.034310089 | Igf1 | 1 |
| 620 | Biological Process | GO:0032635 | interleukin-6 production | 0.25 | 0.02278777 | 0.034310089 | Hgf | 1 |
| 621 | Biological Process | GO:0050671 | positive regulation of lymphocyte proliferation | 0.25 | 0.02278777 | 0.034310089 | Igf1 | 1 |
| 622 | Biological Process | GO:0001678 | cellular glucose homeostasis | 0.25 | 0.022952659 | 0.034447409 | Igf1 | 1 |
| 623 | Biological Process | GO:0007292 | female gamete generation | 0.25 | 0.022952659 | 0.034447409 | Igf1 | 1 |
| 624 | Biological Process | GO:0032946 | positive regulation of mononuclear cell proliferation | 0.25 | 0.023117527 | 0.03458382 | Igf1 | 1 |
| 625 | Biological Process | GO:0042552 | myelination | 0.25 | 0.023117527 | 0.03458382 | Hgf | 1 |
| 626 | Biological Process | GO:0050728 | negative regulation of inflammatory response | 0.25 | 0.023282373 | 0.034774791 | Hgf | 1 |
| 627 | Biological Process | GO:0009156 | ribonucleoside monophosphate biosynthetic process | 0.25 | 0.023447199 | 0.034909445 | Igf1 | 1 |
| 628 | Biological Process | GO:0042133 | neurotransmitter metabolic process | 0.25 | 0.023447199 | 0.034909445 | Igf1 | 1 |
| 629 | Biological Process | GO:0007272 | ensheathment of neurons | 0.25 | 0.023612005 | 0.034932317 | Hgf | 1 |
| 630 | Biological Process | GO:0008366 | axon ensheathment | 0.25 | 0.023612005 | 0.034932317 | Hgf | 1 |
| 631 | Biological Process | GO:0009142 | nucleoside triphosphate biosynthetic process | 0.25 | 0.023612005 | 0.034932317 | Igf1 | 1 |
| 632 | Biological Process | GO:0072524 | pyridine-containing compound metabolic process | 0.25 | 0.023612005 | 0.034932317 | Igf1 | 1 |
| 633 | Biological Process | GO:0010675 | regulation of cellular carbohydrate metabolic process | 0.25 | 0.023776789 | 0.035120533 | Igf1 | 1 |
| 634 | Biological Process | GO:0045834 | positive regulation of lipid metabolic process | 0.25 | 0.023941552 | 0.035308125 | Igf1 | 1 |
| 635 | Biological Process | GO:0070665 | positive regulation of leukocyte proliferation | 0.25 | 0.024106295 | 0.035495096 | Igf1 | 1 |
| 636 | Biological Process | GO:0009124 | nucleoside monophosphate biosynthetic process | 0.25 | 0.024435717 | 0.035867183 | Igf1 | 1 |
| 637 | Biological Process | GO:0021543 | pallium development | 0.25 | 0.024435717 | 0.035867183 | Egfr | 1 |
| 638 | Biological Process | GO:0051147 | regulation of muscle cell differentiation | 0.25 | 0.024600397 | 0.036052306 | Igf1 | 1 |
| 639 | Biological Process | GO:0006694 | steroid biosynthetic process | 0.25 | 0.024765057 | 0.0361802 | Igf1 | 1 |
| 640 | Biological Process | GO:1905475 | regulation of protein localization to membrane | 0.25 | 0.024765057 | 0.0361802 | Egfr | 1 |
| 641 | Biological Process | GO:0006733 | oxidoreduction coenzyme metabolic process | 0.25 | 0.025423485 | 0.036854199 | Igf1 | 1 |
| 642 | Biological Process | GO:0045931 | positive regulation of mitotic cell cycle | 0.25 | 0.025423485 | 0.036854199 | Igf1 | 1 |
| 643 | Biological Process | GO:0046890 | regulation of lipid biosynthetic process | 0.25 | 0.025423485 | 0.036854199 | Igf1 | 1 |
| 644 | Biological Process | GO:0071897 | DNA biosynthetic process | 0.25 | 0.025423485 | 0.036854199 | Hgf | 1 |
| 645 | Biological Process | GO:0097237 | cellular response to toxic substance | 0.25 | 0.025423485 | 0.036854199 | Hgf | 1 |
| 646 | Biological Process | GO:0043409 | negative regulation of MAPK cascade | 0.25 | 0.02558804 | 0.03703532 | Igf1 | 1 |
| 647 | Biological Process | GO:0031047 | gene silencing by RNA | 0.25 | 0.025752574 | 0.03715842 | Egfr | 1 |
| 648 | Biological Process | GO:0050821 | protein stabilization | 0.25 | 0.025752574 | 0.03715842 | Igf1 | 1 |
| 649 | Biological Process | GO:0046887 | positive regulation of hormone secretion | 0.25 | 0.025917087 | 0.037338176 | Egfr | 1 |
| 650 | Biological Process | GO:0043401 | steroid hormone mediated signaling pathway | 0.25 | 0.026081579 | 0.037517349 | Igf1 | 1 |
| 651 | Biological Process | GO:0009266 | response to temperature stimulus | 0.25 | 0.026246051 | 0.037585721 | Igf1 | 1 |
| 652 | Biological Process | GO:0007088 | regulation of mitotic nuclear division | 0.25 | 0.026410502 | 0.037585721 | Igf1 | 1 |
| 653 | Biological Process | GO:0008360 | regulation of cell shape | 0.25 | 0.026410502 | 0.037585721 | Ccl11 | 1 |
| 654 | Biological Process | GO:0008643 | carbohydrate transport | 0.25 | 0.026410502 | 0.037585721 | Igf1 | 1 |
| 655 | Biological Process | GO:0030833 | regulation of actin filament polymerization | 0.25 | 0.026410502 | 0.037585721 | Ccl11 | 1 |
| 656 | Biological Process | GO:0035296 | regulation of tube diameter | 0.25 | 0.026410502 | 0.037585721 | Egfr | 1 |
| 657 | Biological Process | GO:0097746 | regulation of blood vessel diameter | 0.25 | 0.026410502 | 0.037585721 | Egfr | 1 |
| 658 | Biological Process | GO:0006606 | protein import into nucleus | 0.25 | 0.026574931 | 0.037762251 | Igf1 | 1 |
| 659 | Biological Process | GO:0016202 | regulation of striated muscle tissue development | 0.25 | 0.026739341 | 0.037880732 | Igf1 | 1 |
| 660 | Biological Process | GO:0030902 | hindbrain development | 0.25 | 0.026739341 | 0.037880732 | Igf1 | 1 |
| 661 | Biological Process | GO:1900542 | regulation of purine nucleotide metabolic process | 0.25 | 0.027068096 | 0.038288457 | Igf1 | 1 |
| 662 | Biological Process | GO:0042129 | regulation of T cell proliferation | 0.25 | 0.027232443 | 0.038346889 | Igf1 | 1 |
| 663 | Biological Process | GO:0051170 | import into nucleus | 0.25 | 0.027232443 | 0.038346889 | Igf1 | 1 |
| 664 | Biological Process | GO:1901861 | regulation of muscle tissue development | 0.25 | 0.027232443 | 0.038346889 | Igf1 | 1 |
| 665 | Biological Process | GO:0048634 | regulation of muscle organ development | 0.25 | 0.027396769 | 0.038520269 | Igf1 | 1 |
| 666 | Biological Process | GO:0098727 | maintenance of cell number | 0.25 | 0.027561074 | 0.038576205 | Igf1 | 1 |
| 667 | Biological Process | GO:2000241 | regulation of reproductive process | 0.25 | 0.027561074 | 0.038576205 | Igf1 | 1 |
| 668 | Biological Process | GO:0006109 | regulation of carbohydrate metabolic process | 0.25 | 0.027725358 | 0.038576205 | Igf1 | 1 |
| 669 | Biological Process | GO:0009749 | response to glucose | 0.25 | 0.027725358 | 0.038576205 | Igf1 | 1 |
| 670 | Biological Process | GO:0019722 | calcium-mediated signaling | 0.25 | 0.027725358 | 0.038576205 | Igf1 | 1 |
| 671 | Biological Process | GO:0034404 | nucleobase-containing small molecule biosynthetic process | 0.25 | 0.027725358 | 0.038576205 | Igf1 | 1 |
| 672 | Biological Process | GO:0071230 | cellular response to amino acid stimulus | 0.25 | 0.027725358 | 0.038576205 | Egfr | 1 |
| 673 | Biological Process | GO:0009108 | coenzyme biosynthetic process | 0.25 | 0.027889621 | 0.038747096 | Igf1 | 1 |
| 674 | Biological Process | GO:0001890 | placenta development | 0.25 | 0.028053864 | 0.03885701 | Egfr | 1 |
| 675 | Biological Process | GO:0006140 | regulation of nucleotide metabolic process | 0.25 | 0.028218085 | 0.03885701 | Igf1 | 1 |
| 676 | Biological Process | GO:0009612 | response to mechanical stimulus | 0.25 | 0.028218085 | 0.03885701 | Igf1 | 1 |
| 677 | Biological Process | GO:0009746 | response to hexose | 0.25 | 0.028218085 | 0.03885701 | Igf1 | 1 |
| 678 | Biological Process | GO:0030041 | actin filament polymerization | 0.25 | 0.028218085 | 0.03885701 | Ccl11 | 1 |
| 679 | Biological Process | GO:0050880 | regulation of blood vessel size | 0.25 | 0.028218085 | 0.03885701 | Egfr | 1 |
| 680 | Biological Process | GO:0034284 | response to monosaccharide | 0.25 | 0.028382286 | 0.038911199 | Igf1 | 1 |
| 681 | Biological Process | GO:0035150 | regulation of tube size | 0.25 | 0.028382286 | 0.038911199 | Egfr | 1 |
| 682 | Biological Process | GO:0046328 | regulation of JNK cascade | 0.25 | 0.028382286 | 0.038911199 | Egfr | 1 |
| 683 | Biological Process | GO:0071356 | cellular response to tumor necrosis factor | 0.25 | 0.028546466 | 0.039078984 | Ccl11 | 1 |
| 684 | Biological Process | GO:0061351 | neural precursor cell proliferation | 0.25 | 0.028710626 | 0.03924625 | Igf1 | 1 |
| 685 | Biological Process | GO:0071695 | anatomical structure maturation | 0.25 | 0.028874764 | 0.039412999 | Igf1 | 1 |
| 686 | Biological Process | GO:0055002 | striated muscle cell development | 0.25 | 0.029202979 | 0.039802893 | Igf1 | 1 |
| 687 | Biological Process | GO:0000082 | G1/S transition of mitotic cell cycle | 0.25 | 0.029367055 | 0.039968262 | Egfr | 1 |
| 688 | Biological Process | GO:0008064 | regulation of actin polymerization or depolymerization | 0.25 | 0.02953111 | 0.040122774 | Ccl11 | 1 |
| 689 | Biological Process | GO:0030832 | regulation of actin filament length | 0.25 | 0.029695144 | 0.040122774 | Ccl11 | 1 |
| 690 | Biological Process | GO:0043200 | response to amino acid | 0.25 | 0.029695144 | 0.040122774 | Egfr | 1 |
| 691 | Biological Process | GO:0050870 | positive regulation of T cell activation | 0.25 | 0.029695144 | 0.040122774 | Igf1 | 1 |
| 692 | Biological Process | GO:0097529 | myeloid leukocyte migration | 0.25 | 0.029695144 | 0.040122774 | Ccl11 | 1 |
| 693 | Biological Process | GO:0032869 | cellular response to insulin stimulus | 0.25 | 0.029859158 | 0.040286166 | Igf1 | 1 |
| 694 | Biological Process | GO:0035265 | organ growth | 0.25 | 0.030187123 | 0.040611453 | Igf1 | 1 |
| 695 | Biological Process | GO:0043901 | negative regulation of multi-organism process | 0.25 | 0.030187123 | 0.040611453 | Igf1 | 1 |
| 696 | Biological Process | GO:0062013 | positive regulation of small molecule metabolic process | 0.25 | 0.030351074 | 0.040714856 | Igf1 | 1 |
| 697 | Biological Process | GO:2001020 | regulation of response to DNA damage stimulus | 0.25 | 0.030351074 | 0.040714856 | Egfr | 1 |
| 698 | Biological Process | GO:0009743 | response to carbohydrate | 0.25 | 0.030515005 | 0.040876117 | Igf1 | 1 |
| 699 | Biological Process | GO:0006006 | glucose metabolic process | 0.25 | 0.031006672 | 0.041322444 | Igf1 | 1 |
| 700 | Biological Process | GO:0001649 | osteoblast differentiation | 0.25 | 0.031170519 | 0.041322444 | Igf1 | 1 |
| 701 | Biological Process | GO:0007254 | JNK cascade | 0.25 | 0.031170519 | 0.041322444 | Egfr | 1 |
| 702 | Biological Process | GO:0034612 | response to tumor necrosis factor | 0.25 | 0.031170519 | 0.041322444 | Ccl11 | 1 |
| 703 | Biological Process | GO:0046434 | organophosphate catabolic process | 0.25 | 0.031170519 | 0.041322444 | Igf1 | 1 |
| 704 | Biological Process | GO:0051783 | regulation of nuclear division | 0.25 | 0.031170519 | 0.041322444 | Igf1 | 1 |
| 705 | Biological Process | GO:0071241 | cellular response to inorganic substance | 0.25 | 0.031170519 | 0.041322444 | Egfr | 1 |
| 706 | Biological Process | GO:0009755 | hormone-mediated signaling pathway | 0.25 | 0.031334345 | 0.041322444 | Igf1 | 1 |
| 707 | Biological Process | GO:0071236 | cellular response to antibiotic | 0.25 | 0.031334345 | 0.041322444 | Hgf | 1 |
| 708 | Biological Process | GO:1902905 | positive regulation of supramolecular fiber organization | 0.25 | 0.031334345 | 0.041322444 | Ccl11 | 1 |
| 709 | Biological Process | GO:1903039 | positive regulation of leukocyte cell-cell adhesion | 0.25 | 0.031334345 | 0.041322444 | Igf1 | 1 |
| 710 | Biological Process | GO:0044843 | cell cycle G1/S phase transition | 0.25 | 0.031498151 | 0.04147996 | Egfr | 1 |
| 711 | Biological Process | GO:0007623 | circadian rhythm | 0.25 | 0.031825701 | 0.041852363 | Igf1 | 1 |
| 712 | Biological Process | GO:0030595 | leukocyte chemotaxis | 0.25 | 0.031989444 | 0.041949692 | Ccl11 | 1 |
| 713 | Biological Process | GO:0055001 | muscle cell development | 0.25 | 0.031989444 | 0.041949692 | Igf1 | 1 |
| 714 | Biological Process | GO:0003018 | vascular process in circulatory system | 0.25 | 0.032153167 | 0.041987725 | Egfr | 1 |
| 715 | Biological Process | GO:0051099 | positive regulation of binding | 0.25 | 0.032153167 | 0.041987725 | Igf1 | 1 |
| 716 | Biological Process | GO:0090316 | positive regulation of intracellular protein transport | 0.25 | 0.032153167 | 0.041987725 | Igf1 | 1 |
| 717 | Biological Process | GO:0006898 | receptor-mediated endocytosis | 0.25 | 0.032316869 | 0.042083945 | Egfr | 1 |
| 718 | Biological Process | GO:0046425 | regulation of JAK-STAT cascade | 0.25 | 0.032316869 | 0.042083945 | Igf1 | 1 |
| 719 | Biological Process | GO:0060348 | bone development | 0.25 | 0.03248055 | 0.042238267 | Igf1 | 1 |
| 720 | Biological Process | GO:1904892 | regulation of STAT cascade | 0.25 | 0.03264421 | 0.042392134 | Igf1 | 1 |
| 721 | Biological Process | GO:0042098 | T cell proliferation | 0.25 | 0.03280785 | 0.042545547 | Igf1 | 1 |
| 722 | Biological Process | GO:0008154 | actin polymerization or depolymerization | 0.25 | 0.032971469 | 0.042580557 | Ccl11 | 1 |
| 723 | Biological Process | GO:0030522 | intracellular receptor signaling pathway | 0.25 | 0.032971469 | 0.042580557 | Igf1 | 1 |
| 724 | Biological Process | GO:2000027 | regulation of organ morphogenesis | 0.25 | 0.032971469 | 0.042580557 | Hgf | 1 |
| 725 | Biological Process | GO:0031348 | negative regulation of defense response | 0.25 | 0.033135067 | 0.042732811 | Hgf | 1 |
| 726 | Biological Process | GO:0048639 | positive regulation of developmental growth | 0.25 | 0.033298644 | 0.042825629 | Igf1 | 1 |
| 727 | Biological Process | GO:0071383 | cellular response to steroid hormone stimulus | 0.25 | 0.033298644 | 0.042825629 | Igf1 | 1 |
| 728 | Biological Process | GO:0042445 | hormone metabolic process | 0.25 | 0.033462201 | 0.042917912 | Igf1 | 1 |
| 729 | Biological Process | GO:0048839 | inner ear development | 0.25 | 0.033462201 | 0.042917912 | Igf1 | 1 |
| 730 | Biological Process | GO:0016051 | carbohydrate biosynthetic process | 0.25 | 0.033789252 | 0.043278014 | Igf1 | 1 |
| 731 | Biological Process | GO:0035264 | multicellular organism growth | 0.25 | 0.03411622 | 0.043637025 | Igf1 | 1 |
| 732 | Biological Process | GO:0007259 | JAK-STAT cascade | 0.25 | 0.034279673 | 0.043786194 | Igf1 | 1 |
| 733 | Biological Process | GO:0007163 | establishment or maintenance of cell polarity | 0.25 | 0.034606516 | 0.043963441 | Igf1 | 1 |
| 734 | Biological Process | GO:0032868 | response to insulin | 0.25 | 0.034606516 | 0.043963441 | Igf1 | 1 |
| 735 | Biological Process | GO:0034764 | positive regulation of transmembrane transport | 0.25 | 0.034606516 | 0.043963441 | Igf1 | 1 |
| 736 | Biological Process | GO:0097696 | STAT cascade | 0.25 | 0.034606516 | 0.043963441 | Igf1 | 1 |
| 737 | Biological Process | GO:0032271 | regulation of protein polymerization | 0.25 | 0.034933277 | 0.044258284 | Ccl11 | 1 |
| 738 | Biological Process | GO:0060828 | regulation of canonical Wnt signaling pathway | 0.25 | 0.034933277 | 0.044258284 | Egfr | 1 |
| 739 | Biological Process | GO:0051495 | positive regulation of cytoskeleton organization | 0.25 | 0.035096626 | 0.044405068 | Ccl11 | 1 |
| 740 | Biological Process | GO:0043547 | positive regulation of GTPase activity | 0.25 | 0.035259954 | 0.044551429 | Ccl11 | 1 |
| 741 | Biological Process | GO:0030324 | lung development | 0.25 | 0.035423262 | 0.044697368 | Igf1 | 1 |
| 742 | Biological Process | GO:0017038 | protein import | 0.25 | 0.035586549 | 0.044842888 | Igf1 | 1 |
| 743 | Biological Process | GO:0019318 | hexose metabolic process | 0.25 | 0.035749815 | 0.044927523 | Igf1 | 1 |
| 744 | Biological Process | GO:0051188 | cofactor biosynthetic process | 0.25 | 0.035749815 | 0.044927523 | Igf1 | 1 |
| 745 | Biological Process | GO:0030323 | respiratory tube development | 0.25 | 0.035913061 | 0.045011678 | Igf1 | 1 |
| 746 | Biological Process | GO:0090276 | regulation of peptide hormone secretion | 0.25 | 0.035913061 | 0.045011678 | Egfr | 1 |
| 747 | Biological Process | GO:0001894 | tissue homeostasis | 0.25 | 0.036076285 | 0.045155725 | Egfr | 1 |
| 748 | Biological Process | GO:0030278 | regulation of ossification | 0.25 | 0.036402673 | 0.045442589 | Igf1 | 1 |
| 749 | Biological Process | GO:0050670 | regulation of lymphocyte proliferation | 0.25 | 0.036402673 | 0.045442589 | Igf1 | 1 |
| 750 | Biological Process | GO:0046034 | ATP metabolic process | 0.25 | 0.036565835 | 0.045585408 | Igf1 | 1 |
| 751 | Biological Process | GO:0032944 | regulation of mononuclear cell proliferation | 0.25 | 0.036728977 | 0.045606366 | Igf1 | 1 |
| 752 | Biological Process | GO:0045930 | negative regulation of mitotic cell cycle | 0.25 | 0.036728977 | 0.045606366 | Egfr | 1 |
| 753 | Biological Process | GO:0090257 | regulation of muscle system process | 0.25 | 0.036728977 | 0.045606366 | Igf1 | 1 |
| 754 | Biological Process | GO:0022409 | positive regulation of cell-cell adhesion | 0.25 | 0.037055199 | 0.04588955 | Igf1 | 1 |
| 755 | Biological Process | GO:1901215 | negative regulation of neuron death | 0.25 | 0.037055199 | 0.04588955 | Igf1 | 1 |
| 756 | Biological Process | GO:0017148 | negative regulation of translation | 0.25 | 0.037218278 | 0.046030542 | Egfr | 1 |
| 757 | Biological Process | GO:0071375 | cellular response to peptide hormone stimulus | 0.25 | 0.037544375 | 0.046311334 | Igf1 | 1 |
| 758 | Biological Process | GO:1903522 | regulation of blood circulation | 0.25 | 0.037544375 | 0.046311334 | Egfr | 1 |
| 759 | Biological Process | GO:0045444 | fat cell differentiation | 0.25 | 0.037707393 | 0.046451136 | Igf1 | 1 |
| 760 | Biological Process | GO:0070663 | regulation of leukocyte proliferation | 0.25 | 0.038033366 | 0.046729563 | Igf1 | 1 |
| 761 | Biological Process | GO:0090068 | positive regulation of cell cycle process | 0.25 | 0.038033366 | 0.046729563 | Igf1 | 1 |
| 762 | Biological Process | GO:0001818 | negative regulation of cytokine production | 0.25 | 0.038196321 | 0.04686819 | Hgf | 1 |
| 763 | Biological Process | GO:0010038 | response to metal ion | 0.25 | 0.038359256 | 0.047006428 | Egfr | 1 |
| 764 | Biological Process | GO:0043583 | ear development | 0.25 | 0.03852217 | 0.047144279 | Igf1 | 1 |
| 765 | Biological Process | GO:0021537 | telencephalon development | 0.25 | 0.039010788 | 0.047617607 | Egfr | 1 |
| 766 | Biological Process | GO:0072330 | monocarboxylic acid biosynthetic process | 0.25 | 0.039010788 | 0.047617607 | Igf1 | 1 |
| 767 | Biological Process | GO:0031334 | positive regulation of protein complex assembly | 0.25 | 0.039173619 | 0.047691841 | Ccl11 | 1 |
| 768 | Biological Process | GO:0046578 | regulation of Ras protein signal transduction | 0.25 | 0.039173619 | 0.047691841 | Igf1 | 1 |
| 769 | Biological Process | GO:0048588 | developmental cell growth | 0.25 | 0.03933643 | 0.047827779 | Igf1 | 1 |
| 770 | Biological Process | GO:0033500 | carbohydrate homeostasis | 0.25 | 0.039824737 | 0.048233328 | Igf1 | 1 |
| 771 | Biological Process | GO:0034249 | negative regulation of cellular amide metabolic process | 0.25 | 0.039824737 | 0.048233328 | Egfr | 1 |
| 772 | Biological Process | GO:0042593 | glucose homeostasis | 0.25 | 0.039824737 | 0.048233328 | Igf1 | 1 |
| 773 | Biological Process | GO:0048738 | cardiac muscle tissue development | 0.25 | 0.039987465 | 0.048367762 | Igf1 | 1 |
| 774 | Biological Process | GO:0046777 | protein autophosphorylation | 0.25 | 0.040150172 | 0.048501823 | Egfr | 1 |
| 775 | Biological Process | GO:0005996 | monosaccharide metabolic process | 0.25 | 0.040638169 | 0.048971428 | Igf1 | 1 |
| 776 | Biological Process | GO:0009167 | purine ribonucleoside monophosphate metabolic process | 0.25 | 0.040800794 | 0.048971428 | Igf1 | 1 |
| 777 | Biological Process | GO:0009205 | purine ribonucleoside triphosphate metabolic process | 0.25 | 0.040800794 | 0.048971428 | Igf1 | 1 |
| 778 | Biological Process | GO:0015980 | energy derivation by oxidation of organic compounds | 0.25 | 0.040800794 | 0.048971428 | Igf1 | 1 |
| 779 | Biological Process | GO:0060541 | respiratory system development | 0.25 | 0.040800794 | 0.048971428 | Igf1 | 1 |
| 780 | Biological Process | GO:0009126 | purine nucleoside monophosphate metabolic process | 0.25 | 0.040963397 | 0.04910356 | Igf1 | 1 |
| 781 | Biological Process | GO:0031647 | regulation of protein stability | 0.25 | 0.04112598 | 0.049172368 | Igf1 | 1 |
| 782 | Biological Process | GO:0072659 | protein localization to plasma membrane | 0.25 | 0.04112598 | 0.049172368 | Egfr | 1 |
| 783 | Biological Process | GO:0110053 | regulation of actin filament organization | 0.25 | 0.041288543 | 0.049303688 | Ccl11 | 1 |
| 784 | Biological Process | GO:0009161 | ribonucleoside monophosphate metabolic process | 0.25 | 0.041451084 | 0.04930886 | Igf1 | 1 |
| 785 | Biological Process | GO:0009199 | ribonucleoside triphosphate metabolic process | 0.25 | 0.041451084 | 0.04930886 | Igf1 | 1 |
| 786 | Biological Process | GO:0061448 | connective tissue development | 0.25 | 0.041451084 | 0.04930886 | Igf1 | 1 |
| 787 | Biological Process | GO:0032388 | positive regulation of intracellular transport | 0.25 | 0.041776106 | 0.04951083 | Igf1 | 1 |
| 788 | Biological Process | GO:1901990 | regulation of mitotic cell cycle phase transition | 0.25 | 0.041776106 | 0.04951083 | Egfr | 1 |
| 789 | Biological Process | GO:0009152 | purine ribonucleotide biosynthetic process | 0.25 | 0.041938585 | 0.04951083 | Igf1 | 1 |
| 790 | Biological Process | GO:0048545 | response to steroid hormone | 0.25 | 0.041938585 | 0.04951083 | Igf1 | 1 |
| 791 | Biological Process | GO:0051924 | regulation of calcium ion transport | 0.25 | 0.041938585 | 0.04951083 | Igf1 | 1 |
| 792 | Biological Process | GO:0060560 | developmental growth involved in morphogenesis | 0.25 | 0.041938585 | 0.04951083 | Ccl11 | 1 |
| 793 | Biological Process | GO:0009144 | purine nucleoside triphosphate metabolic process | 0.25 | 0.042263483 | 0.049831471 | Igf1 | 1 |
| 794 | Biological Process | GO:1901653 | cellular response to peptide | 0.25 | 0.042425901 | 0.049959971 | Igf1 | 1 |
| 1 | Molecular Function | GO:0048018 | receptor ligand activity | 0.75 | 3.67E-05 | 0.00165088 | Igf1/Ccl11/Hgf | 3 |
| 2 | Molecular Function | GO:0005178 | integrin binding | 0.5 | 0.000139521 | 0.00313922 | Igf1/Egfr | 2 |
| 3 | Molecular Function | GO:0008083 | growth factor activity | 0.5 | 0.000237117 | 0.00355675 | Igf1/Hgf | 2 |
| 4 | Molecular Function | GO:0050839 | cell adhesion molecule binding | 0.5 | 0.002525571 | 0.014629381 | Igf1/Egfr | 2 |
| 5 | Molecular Function | GO:0004716 | signal transducer, downstream of receptor, with protein tyrosine kinase activity | 0.25 | 0.001214329 | 0.012489428 | Egfr | 1 |
| 6 | Molecular Function | GO:0030235 | nitric-oxide synthase regulator activity | 0.25 | 0.001387714 | 0.012489428 | Egfr | 1 |
| 7 | Molecular Function | GO:0030546 | receptor activator activity | 0.25 | 0.002081029 | 0.014629381 | Igf1 | 1 |
| 8 | Molecular Function | GO:0005159 | insulin-like growth factor receptor binding | 0.25 | 0.002600779 | 0.014629381 | Igf1 | 1 |
| 9 | Molecular Function | GO:0005158 | insulin receptor binding | 0.25 | 0.00450479 | 0.021828064 | Igf1 | 1 |
| 10 | Molecular Function | GO:0043539 | protein serine/threonine kinase activator activity | 0.25 | 0.004850681 | 0.021828064 | Igf1 | 1 |
| 11 | Molecular Function | GO:0042056 | chemoattractant activity | 0.25 | 0.006578783 | 0.026913202 | Hgf | 1 |
| 12 | Molecular Function | GO:0008009 | chemokine activity | 0.25 | 0.007441989 | 0.02755261 | Ccl11 | 1 |
| 13 | Molecular Function | GO:0048020 | CCR chemokine receptor binding | 0.25 | 0.007959643 | 0.02755261 | Ccl11 | 1 |
| 14 | Molecular Function | GO:0004714 | transmembrane receptor protein tyrosine kinase activity | 0.25 | 0.010544875 | 0.031077552 | Egfr | 1 |
| 15 | Molecular Function | GO:0042379 | chemokine receptor binding | 0.25 | 0.011405496 | 0.031077552 | Ccl11 | 1 |
| 16 | Molecular Function | GO:0030295 | protein kinase activator activity | 0.25 | 0.011749586 | 0.031077552 | Igf1 | 1 |
| 17 | Molecular Function | GO:0031434 | mitogen-activated protein kinase kinase binding | 0.25 | 0.01278132 | 0.031077552 | Egfr | 1 |
| 18 | Molecular Function | GO:0019209 | kinase activator activity | 0.25 | 0.012953197 | 0.031077552 | Igf1 | 1 |
| 19 | Molecular Function | GO:0019199 | transmembrane receptor protein kinase activity | 0.25 | 0.013468693 | 0.031077552 | Egfr | 1 |
| 20 | Molecular Function | GO:0004709 | MAP kinase kinase kinase activity | 0.25 | 0.013812246 | 0.031077552 | Egfr | 1 |
| 21 | Molecular Function | GO:0042562 | hormone binding | 0.25 | 0.018441422 | 0.038421152 | Egfr | 1 |
| 22 | Molecular Function | GO:0005496 | steroid binding | 0.25 | 0.018783674 | 0.038421152 | Igf1 | 1 |
| 23 | Molecular Function | GO:0004702 | signal transducer, downstream of receptor, with serine/threonine kinase activity | 0.25 | 0.021176936 | 0.041433136 | Egfr | 1 |
| 24 | Molecular Function | GO:0005179 | hormone activity | 0.25 | 0.022371924 | 0.041947357 | Igf1 | 1 |
| 25 | Molecular Function | GO:0019838 | growth factor binding | 0.25 | 0.025269481 | 0.041979338 | Egfr | 1 |
| 26 | Molecular Function | GO:0005057 | signal transducer activity, downstream of receptor | 0.25 | 0.025950322 | 0.041979338 | Egfr | 1 |
| 27 | Molecular Function | GO:0019903 | protein phosphatase binding | 0.25 | 0.025950322 | 0.041979338 | Egfr | 1 |
| 28 | Molecular Function | GO:0004713 | protein tyrosine kinase activity | 0.25 | 0.026120477 | 0.041979338 | Egfr | 1 |
| 29 | Molecular Function | GO:0051015 | actin filament binding | 0.25 | 0.0303671 | 0.046017582 | Egfr | 1 |
| 30 | Molecular Function | GO:0019887 | protein kinase regulator activity | 0.25 | 0.031045269 | 0.046017582 | Igf1 | 1 |
| 31 | Molecular Function | GO:0005516 | calmodulin binding | 0.25 | 0.032231208 | 0.046017582 | Egfr | 1 |
| 32 | Molecular Function | GO:0004252 | serine-type endopeptidase activity | 0.25 | 0.032739134 | 0.046017582 | Hgf | 1 |
| 33 | Molecular Function | GO:0019207 | kinase regulator activity | 0.25 | 0.03476884 | 0.046017582 | Igf1 | 1 |
| 34 | Molecular Function | GO:0019902 | phosphatase binding | 0.25 | 0.03476884 | 0.046017582 | Egfr | 1 |
| 35 | Molecular Function | GO:0008236 | serine-type peptidase activity | 0.25 | 0.037470143 | 0.046597058 | Hgf | 1 |
| 36 | Molecular Function | GO:0005125 | cytokine activity | 0.25 | 0.037976006 | 0.046597058 | Ccl11 | 1 |
| 37 | Molecular Function | GO:0017171 | serine hydrolase activity | 0.25 | 0.038313137 | 0.046597058 | Hgf | 1 |
| 1 | Cellular Component | GO:0016942 | insulin-like growth factor binding protein complex | 0.00 | 0.000847853 | 0.011446012 | Igf1 | 1 |
| 2 | Cellular Component | GO:0036454 | growth factor complex | 0.00 | 0.000847853 | 0.011446012 | Igf1 | 1 |
| 3 | Cellular Component | GO:0031983 | vesicle lumen | 0.00 | 0.001695166 | 0.015256497 | Egfr | 1 |
| 4 | Cellular Component | GO:0005614 | interstitial matrix | 0.00 | 0.0028805 | 0.017382451 | Igf1 | 1 |
| 5 | Cellular Component | GO:0031091 | platelet alpha granule | 0.00 | 0.003218972 | 0.017382451 | Igf1 | 1 |
| 6 | Cellular Component | GO:0005771 | multivesicular body | 0.00 | 0.005923653 | 0.024549505 | Egfr | 1 |
| 7 | Cellular Component | GO:0009925 | basal plasma membrane | 0.00 | 0.007273927 | 0.024549505 | Egfr | 1 |
| 8 | Cellular Component | GO:0031901 | early endosome membrane | 0.00 | 0.007273927 | 0.024549505 | Egfr | 1 |
| 9 | Cellular Component | GO:0045178 | basal part of cell | 0.00 | 0.011148304 | 0.033444913 | Egfr | 1 |
