# Supplementary material for: Huiyang Shengji decoction promotes wound healing in diabetic mice by activating the EGFR/PI3K/ATK pathway
Source: Chin Med. 2021 Nov 2;16:111. doi: 10.1186/s13020-021-00497-0 (PMC8565039; doi:10.1186/s13020-021-00497-0)
Supplement: Supplementary file 4 — Additional file 4: Table S4. KEGG analysis of the differentially-expressed proteins between the control and model group. [file 13020_2021_497_MOESM4_ESM.doc]

|  | **Table S4: KEGG analysis of the differentially-expressed proteins between the control and model group** | | | | | | |
| --- | --- | --- | --- | --- | --- | --- | --- |
|  | ID | Description | GeneRatio | pvalue | p.adjust | geneID | Count |
| 1 | mmu01521 | EGFR tyrosine kinase inhibitor resistance | 0.5 | 1.70E-05 | 0.001209686 | 16000/13649/22339 | 3 |
| 2 | mmu04066 | HIF-1 signaling pathway | 0.5 | 3.86E-05 | 0.001370619 | 16000/13649/22339 | 3 |
| 3 | mmu04510 | Focal adhesion | 0.5 | 0.000259644 | 0.004196869 | 16000/13649/22339 | 3 |
| 4 | mmu05205 | Proteoglycans in cancer | 0.5 | 0.000279429 | 0.004196869 | 16000/13649/22339 | 3 |
| 5 | mmu04015 | Rap1 signaling pathway | 0.5 | 0.000300174 | 0.004196869 | 16000/13649/22339 | 3 |
| 6 | mmu04014 | Ras signaling pathway | 0.5 | 0.000413776 | 0.004196869 | 16000/13649/22339 | 3 |
| 7 | mmu04010 | MAPK signaling pathway | 0.5 | 0.000819505 | 0.007024814 | 16000/13649/22339 | 3 |
| 8 | mmu04151 | PI3K-Akt signaling pathway | 0.5 | 0.001456127 | 0.007952691 | 16000/13649/22339 | 3 |
| 9 | mmu05219 | Bladder cancer | 0.333333333 | 0.000354717 | 0.004196869 | 13649/22339 | 2 |
| 10 | mmu04115 | p53 signaling pathway | 0.333333333 | 0.001064592 | 0.007024814 | 16000/16009 | 2 |
| 11 | mmu05214 | Glioma | 0.333333333 | 0.001064592 | 0.007024814 | 16000/13649 | 2 |
| 12 | mmu05218 | Melanoma | 0.333333333 | 0.001094655 | 0.007024814 | 16000/13649 | 2 |
| 13 | mmu05212 | Pancreatic cancer | 0.333333333 | 0.001187293 | 0.007024814 | 13649/22339 | 2 |
| 14 | mmu01522 | Endocrine resistance | 0.333333333 | 0.001819723 | 0.009228596 | 16000/13649 | 2 |
| 15 | mmu05215 | Prostate cancer | 0.333333333 | 0.001977948 | 0.009362289 | 16000/13649 | 2 |
| 16 | mmu04926 | Relaxin signaling pathway | 0.333333333 | 0.003577669 | 0.015166987 | 13649/22339 | 2 |
| 17 | mmu04068 | FoxO signaling pathway | 0.333333333 | 0.003631532 | 0.015166987 | 16000/13649 | 2 |
| 18 | mmu05224 | Breast cancer | 0.333333333 | 0.004485393 | 0.017692385 | 16000/13649 | 2 |
| 19 | mmu05202 | Transcriptional misregulation in cancer | 0.333333333 | 0.00687974 | 0.025708501 | 16000/16009 | 2 |
| 20 | mmu04062 | Chemokine signaling pathway | 0.333333333 | 0.008096676 | 0.0287432 | 20299/14825 | 2 |
| 21 | mmu05167 | Kaposi sarcoma-associated herpesvirus infection | 0.333333333 | 0.009575383 | 0.032373915 | 22339/14825 | 2 |
| 22 | mmu05163 | Human cytomegalovirus infection | 0.333333333 | 0.013167375 | 0.04249471 | 13649/22339 | 2 |
| 23 | mmu05206 | MicroRNAs in cancer | 0.333333333 | 0.015741172 | 0.048592314 | 13649/22339 | 2 |
