# Supplementary material for: Huiyang Shengji decoction promotes wound healing in diabetic mice by activating the EGFR/PI3K/ATK pathway
Source: Chin Med. 2021 Nov 2;16:111. doi: 10.1186/s13020-021-00497-0 (PMC8565039; doi:10.1186/s13020-021-00497-0)
Supplement: Supplementary file 5 — Additional file 5: Table S5. KEGG analysis of the differentially-expressed proteins between the HYSJD and model group. [file 13020_2021_497_MOESM5_ESM.doc]

|  | **Table S5: KEGG analysis of the differentially-expressed proteins between the HYSJD and model group** | | | | | | |
| --- | --- | --- | --- | --- | --- | --- | --- |
|  | ID | Description | GeneRatio | pvalue | p.adjust | geneID | Count |
| 1 | mmu05218 | Melanoma | 0.75 | 2.51E-06 | 0.000107124 | Igf1/Egfr/Hgf | 3 |
| 2 | mmu01521 | EGFR tyrosine kinase inhibitor resistance | 0.75 | 3.46E-06 | 0.000107124 | Igf1/Egfr/Hgf | 3 |
| 3 | mmu04510 | Focal adhesion | 0.75 | 5.38E-05 | 0.000772984 | Igf1/Egfr/Hgf | 3 |
| 4 | mmu05205 | Proteoglycans in cancer | 0.75 | 5.80E-05 | 0.000772984 | Igf1/Egfr/Hgf | 3 |
| 5 | mmu04015 | Rap1 signaling pathway | 0.75 | 6.23E-05 | 0.000772984 | Igf1/Egfr/Hgf | 3 |
| 6 | mmu04014 | Ras signaling pathway | 0.75 | 8.63E-05 | 0.000891869 | Igf1/Egfr/Hgf | 3 |
| 7 | mmu04010 | MAPK signaling pathway | 0.75 | 0.000172879 | 0.001531216 | Igf1/Egfr/Hgf | 3 |
| 8 | mmu04151 | PI3K-Akt signaling pathway | 0.75 | 0.00031085 | 0.002409085 | Igf1/Egfr/Hgf | 3 |
| 9 | mmu05214 | Glioma | 0.5 | 0.000430604 | 0.002966384 | Igf1/Egfr | 2 |
| 10 | mmu01522 | Endocrine resistance | 0.5 | 0.000738659 | 0.004528282 | Igf1/Egfr | 2 |
| 11 | mmu05215 | Prostate cancer | 0.5 | 0.000803405 | 0.004528282 | Igf1/Egfr | 2 |
| 12 | mmu04066 | HIF-1 signaling pathway | 0.5 | 0.000940915 | 0.004861393 | Igf1/Egfr | 2 |
| 13 | mmu04068 | FoxO signaling pathway | 0.5 | 0.001483432 | 0.007074828 | Igf1/Egfr | 2 |
| 14 | mmu05224 | Breast cancer | 0.5 | 0.001836676 | 0.007901837 | Igf1/Egfr | 2 |
| 15 | mmu05226 | Gastric cancer | 0.5 | 0.001911735 | 0.007901837 | Egfr/Hgf | 2 |
| 16 | mmu05225 | Hepatocellular carcinoma | 0.5 | 0.002478043 | 0.009602416 | Egfr/Hgf | 2 |
| 17 | mmu05310 | Asthma | 0.25 | 0.012030662 | 0.043876533 | Ccl11 | 1 |
